# Effects of *Aspergillus oryzae*-derived rice-*koji* protein on the sake metabolome

**DOI:** 10.1128/aem.01955-25

**Published:** 2026-02-19

**Authors:** Ryousuke Kataoka, Shinichiro Fukuhara, Shingo Kakiuchi, Minori Kono, Sharon Marie Bahena-Garrido, Yuko Komatsu-Hata, Kazuhiro Iwashita

**Affiliations:** 1 National Research Institute of Brewing 13547, Higashi-hiroshima, Hiroshima, Japan; 2 Department of Molecular Biotechnology, Graduate School of Advanced Science of Matter, Hiroshima University 316842 , Higashi-hiroshima, Hiroshima, Japan; 3 Unit of Biotechnology, Graduate School of Integrated Sciences for Life, Hiroshima University 124684 , Higashi-hiroshima, Hiroshima, Japan; Universita degli Studi di Napoli Federico II, Portici, Italy

**Keywords:** *Aspergillus oryzae*, Japanese sake, metabolome, rice-*koji*, proteome, liquid chromatography–mass spectrometry

## Abstract

Rice-*koji* is produced by cultivating *Aspergillus oryzae* on steamed rice and plays a central role in the production of traditional Japanese alcoholic beverages such as sake and shochu, as well as fermented seasonings like miso and soy sauce. While some functions of the proteins produced by *A. oryzae* during rice-*koji* fermentation have been clarified, the comprehensive protein profile and their specific roles in sake brewing are still not fully understood. We hypothesized that proteins expressed by *A. oryzae* in rice-*koji* contribute in diverse ways to shaping the metabolic characteristics of sake. In this study, we performed proteomic analyses on four types of rice-*koji* to explore rice-*koji* proteins (RKPs) and identified 159 *rkp* genes. From this set, selected targets were used to construct single-gene disruptants (Δ*rkp*), resulting in the generation of 85 distinct Δ*rkp* strains. Sake brewing experiments using these disruptants were carried out to assess the impact of individual *rkp* gene deletions. The results revealed alterations in the mycelial biomass of *A. oryzae* in rice-*koji* and showed clear associations with variations in multiple sake components. Further metabolomic analysis demonstrated that disruption of 57 *rkp* genes led to notable changes in at least one sake-derived metabolite peak. Overall, our findings indicate that RKPs identified through proteomic analysis of rice-*koji* exert a wide range of effects on both the growth of *A. oryzae* and the metabolite composition of sake. These insights are expected to advance our understanding of how RKPs influence sake brewing and the broader biological roles of *A. oryzae*.

**IMPORTANCE:**

Rice-koji plays a crucial role in sake brewing. Although some functions of *Aspergillus oryzae*–derived proteins have been elucidated, most remain poorly understood. In this study, we identified rice-koji proteins (RKPs) via proteomic analysis and investigated their effects on sake composition using gene disruption strains. Analyses of samples from rice-koji preparation and small-scale sake brewing showed that disruption of rkp genes altered *A. oryzae* growth and modified multiple sake component profiles. Notably, rkp genes that influenced fungal biomass in rice-koji were also associated with significant changes in sake composition. These findings reveal the diverse contributions of *A. oryzae* to sake brewing and enhance our understanding of the fermentation potential of filamentous fungi, with potential applications in other fermentation-based industries.

## INTRODUCTION

Sake is a traditional Japanese alcoholic beverage brewed from rice, rice-*koji*, and water. The primary microorganisms involved in its fermentation are *Aspergillus oryzae* and *Saccharomyces cerevisiae*. Sake production involves a distinctive process called parallel multiple fermentation, in which rice starch is saccharified by *A. oryzae* while the resulting sugars are simultaneously fermented into alcohol by yeast. The flavor and aroma of sake arise from a complex interplay of factors, including the quality of the raw materials, the traits of the microorganisms employed, and the specific conditions of fermentation. Controlling these sensory attributes requires a comprehensive understanding of how brewing parameters influence the composition of sake. In recent years, metabolomic approaches using liquid chromatography-quadrupole time-of-flight mass spectrometry (LC-Q/TOF-MS) have enabled comprehensive profiling of sake components (1, 2). These analytical techniques are increasingly applied to evaluate the relationships between brewing parameters and the resulting chemical composition, providing new insights into the determinants of sake flavor and aroma.

*A. oryzae* is indispensable for the production of traditional Japanese fermented foods and has been used in Japan for over a thousand years. In the brewing industry, *A. oryzae* is cultivated as *koji*, serving as a source of diverse enzymes, vitamins, and organic acids (3). In 2005, the complete genome sequence of the *A. oryzae* RIB40 strain was published (4), enabling extensive post-genomic research. Although genome analyses indicate that *A. oryzae* contains roughly 12,000 genes, the functions of over half remain uncharacterized. Genes encoding starch-degrading and proteolytic enzymes have been well studied, especially in the context of sake brewing, where these enzymes play a crucial role in breaking down rice components (5, 6). In addition to these well-studied enzymes, a few reports have examined other factors, such as browning of sake (7) and blackening of sake cakes (8), although such studies are limited. Most *A. oryzae* strains currently used in sake brewing—referred to as *tane-koji*—have been selected for a balanced production of starch- and protein-degrading enzymes. Previous studies have shown that the expression of hydrolytic enzymes considered important during rice-*koji* production differs markedly between liquid and solid-state culture conditions (9, 10). Proteomic analyses under both conditions have also revealed differences in extracellular protein secretion profiles (11), suggesting that even non-hydrolytic enzyme expression may vary substantially in solid-state culture compared with other cultivation methods. Nevertheless, our understanding of the global protein production by *A. oryzae* during rice-*koji* fermentation remains limited, and the functions of the genes encoding these proteins—as well as their contributions to sake brewing—are still largely unknown.

In this study, we aimed to identify the proteins produced by *A. oryzae* in rice-*koji* and their corresponding genes and to evaluate how disruption of these genes influences rice-*koji* and sake characteristics. We first performed a proteomic analysis to identify rice-*koji* proteins (RKPs) produced by *A. oryzae*. The genes encoding these RKPs were then identified, and gene disruptants were constructed for each target gene. Using these strains, we prepared rice-*koji* and brewed sake through small-scale fermentation. Analysis of the resulting sake revealed that the composition of multiple components varied among the samples, indicating that a substantial number of RKPs contribute to shaping sake composition. To our knowledge, this is the first study to systematically assess the impact of individual *A. oryzae*–derived proteins on sake composition using a genome-informed proteomic approach.

## RESULTS

### Analysis of proteins produced in rice-*koji*

**Fig 1:**
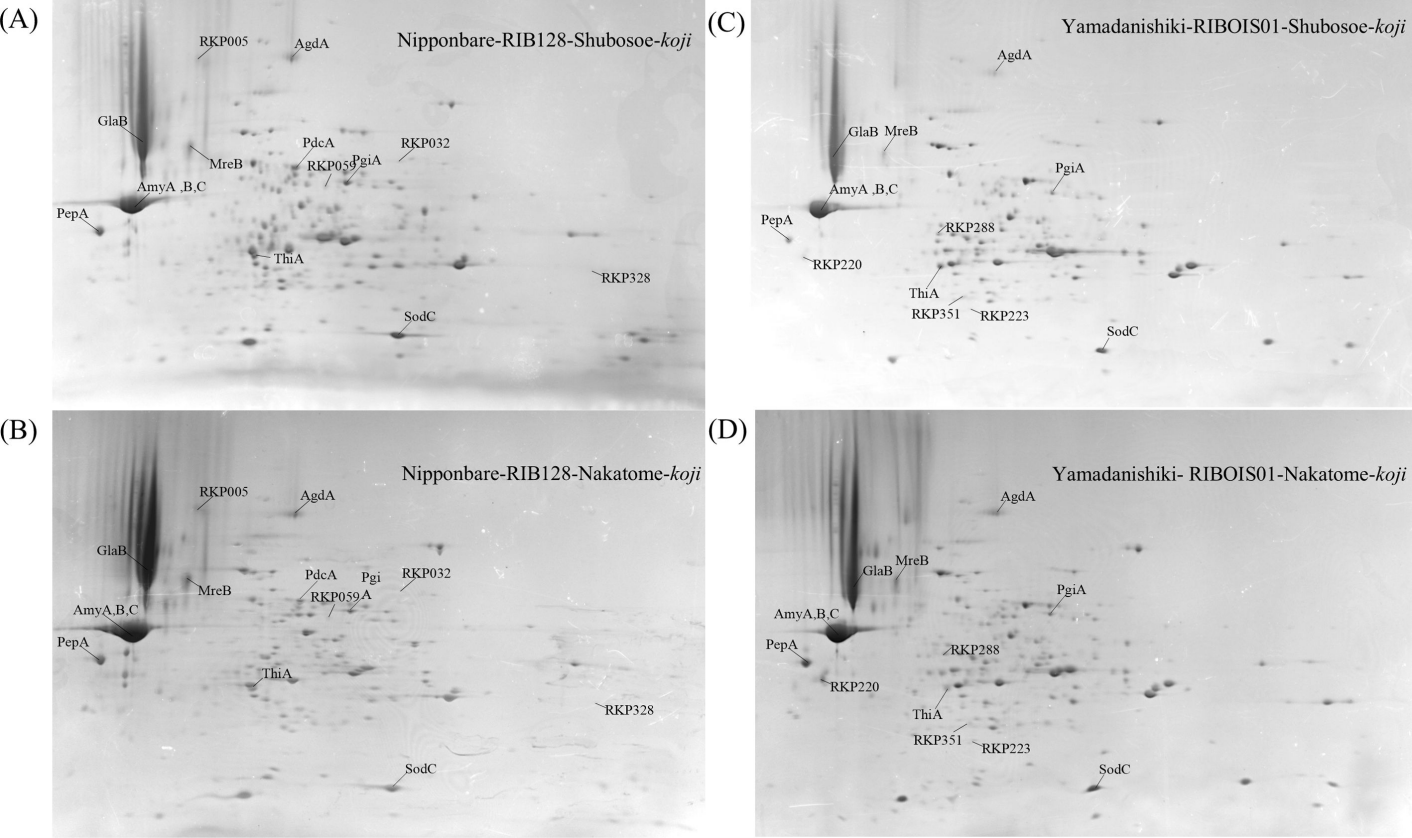
Two-dimensional electrophoresis profiles of proteins extracted from standard *koji* and Ginjo *koji*. (**A**)*Oryza sativa* cv. Nipponbare-RIB128-Shubosoe *koji*, (**B**) Nipponbare-RIB128-Nakatome *koji*, (**C**) *O. sativa* cv. Yamadanishiki-RIBOIS01-Shubosoe-*koji*, and (**D**) Yamadanishiki-RIBOIS01-Nakatome *koji*. Protein spots labeled with RKP identifiers indicate proteins specifically detected under the corresponding rice-*koji* conditions.

Proteins extracted from four types of rice-*koji*—Nipponbare-RIB128-Shubosoe, Nipponbare-RIB128-Nakatome, Yamadanishiki-RIBOIS01-Shubosoe, and Yamadanishiki-RIBOIS01-Nakatome—were analyzed using two-dimensional gel electrophoresis (2-DE) (Fig. 1). Across the 4 conditions, 238 reproducibly detected protein spots were identified by peptide mass fingerprinting (PMF) and tandem mass spectrometry (MS/MS) analysis. Because multiple spots corresponded to the same protein, these identifications were consolidated, resulting in 159 unique *A. oryzae*–derived proteins, which were defined as RKPs (Table S1).

To comprehensively characterize protein production profiles under different rice-*koji* preparation conditions used in sake brewing, we examined the distribution of RKPs across the four experimental conditions based on the 2-DE data (Fig. 1). Particular emphasis was placed on differences between *A. oryzae* strains. Four RKPs (RKP005, RKP032, RKP059, and RKP328) were detected exclusively in rice-*koji* prepared with *A. oryzae* RIB128 (Nipponbare-RIB128-Shubosoe and Nipponbare-RIB128-Nakatome), whereas another four RKPs (RKP220, RKP223, RKP288, and RKP351) were specific to rice-*koji* prepared with *A. oryzae* RIBOIS01 (Yamadanishiki-RIBOIS01-Shubosoe and Yamadanishiki-RIBOIS01-Nakatome). In addition, ten RKPs (RKP007, RKP016, RKP037, RKP046, RKP058, RKP089, RKP142, RKP145, RKP150, and RKP199) were detected at more than twice the abundance in Nipponbare-RIB128 rice-*koji* compared with Yamadanishiki-RIBOIS01 rice-*koji*. Conversely, five RKPs (RKP063, RKP204, RKP231, RKP247, and RKP306) were detected at more than twice the abundance in Yamadanishiki-RIBOIS01 rice-*koji*.

**Fig 2:**
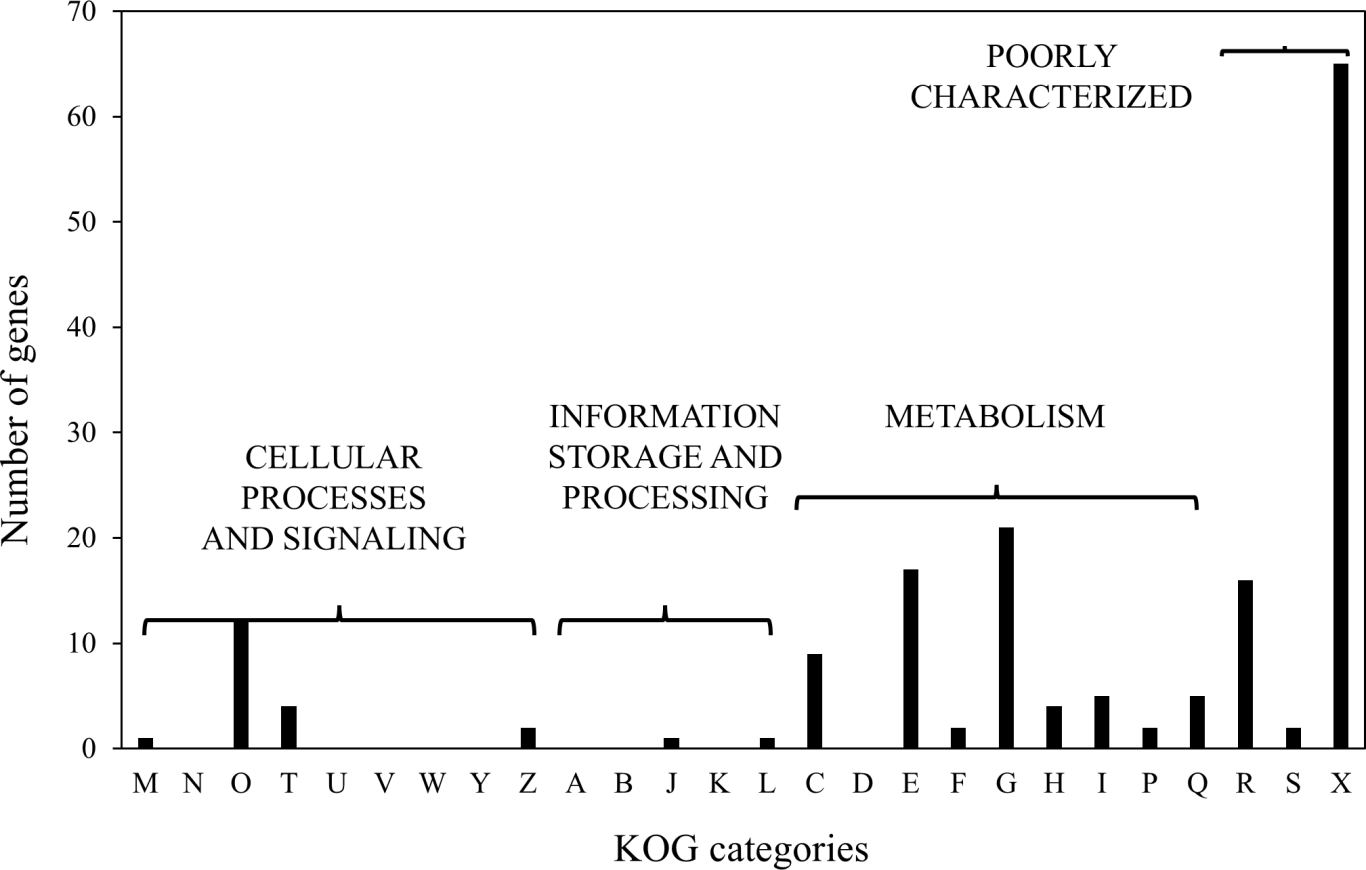
Functional classification of RKP genes based on the KOG database. Functional classification categories: A, RNA processing and modification; B, chromatin structure and dynamics; C, energy production and conversion; D, cell cycle control and mitosis; E, amino acid transport and metabolism; F, nucleotide transport and metabolism; G, carbohydrate transport and metabolism; H, coenzyme metabolism; I, lipid metabolism; J, translation; K, transcription; L, replication and repair; M, cell wall/membrane/envelope biogenesis; N, cell motility; O, post-translational modification, protein turnover, and chaperones; P, inorganic ion transport and metabolism; Q, secondary metabolite biosynthesis, transport, and catabolism; T, signal transduction; U, intracellular trafficking and secretion; Y, nuclear structure; Z, cytoskeleton; R, general function prediction only (typically based on biochemical activity); S, function unknown; X, not classifiable.

To further characterize these RKPs, BLAST searches were performed using UniProt (https://www.uniprot.org) to evaluate sequence similarity with proteins from other organisms. Based on sequence identity and published literature, the RKPs were classified into three groups: Group A, proteins showing ≥80% identity to functionally characterized proteins from other species or with experimentally validated functions in *A. oryzae*; Group B, proteins with putative functions, displaying 50%–79% identity to proteins of known function in other species; and Group C, proteins of unknown function, exhibiting <50% identity to known proteins or lacking functional annotations. In total, 38 RKPs were assigned to Group A, 51 to Group B, and 70 to Group C. Gene identifiers and functional annotations were cross-verified using the Comprehensive *A. oryzae* genome database across the clades (CAoGDX; https://nribf21.nrib.go.jp/CAoGDX/) (Tables S1 and S2). Functional classification of the identified *rkp* genes was then conducted according to Eukaryotic Orthologous Groups (KOG) categories (Fig. 2). Most genes fell into categories R (general function prediction only), S (function unknown), and X (not classified), underscoring that many *rkp* genes remain poorly characterized.

### Construction of *rkp* gene disruptants

To explore the functions of *rkp* genes in sake brewing, we constructed gene disruptants. In total, 125 *rkp* genes were chosen as disruption targets, selected mainly according to their functional annotations and classification outcomes, with special emphasis on those encoding proteins of unknown function (Groups B and C). Disruptants were successfully and reproducibly obtained for 85 *rkp* genes, as listed in Table S1 along with their corresponding gene annotations. Among these, 12 were obtained only as heterokaryotic disruptants. An *adeA*-complemented strain was also constructed as a control for subsequent analyses.

### Growth characteristics of *rkp* gene disruptants under various culture conditions

As an initial step toward clarifying the potential roles of *rkp* genes in sake brewing, we investigated how gene disruption affects growth characteristics. In *A. oryzae*, growth in rice-*koji* is closely linked to enzyme production. Thus, assessing the impact of gene disruption on growth is essential for understanding its possible influence on the brewing process. Furthermore, because *A. oryzae* displays distinct gene regulation under solid- and liquid-state cultivation, we also evaluated growth effects under submerged culture conditions.

**Fig 3:**
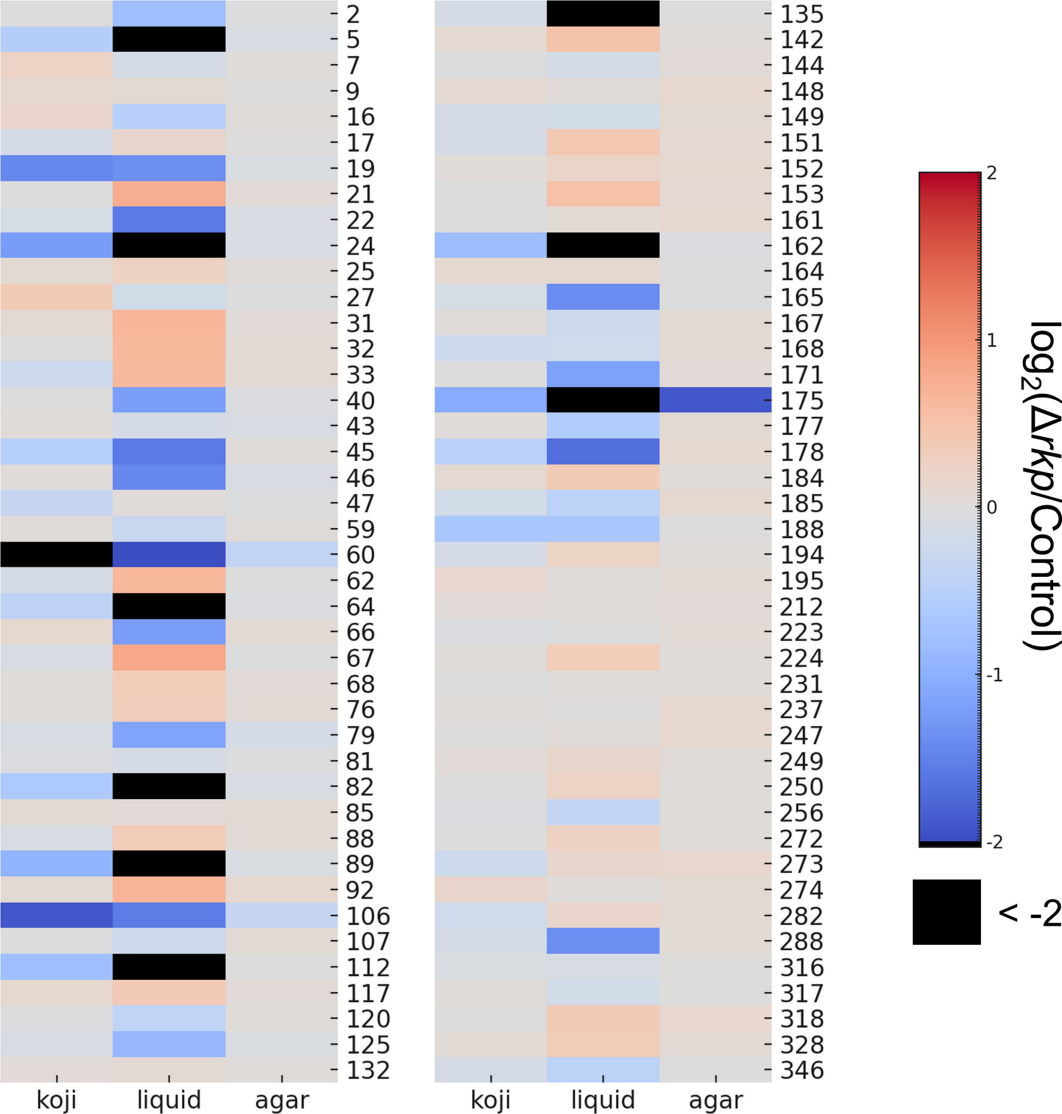
Effects of *rkp* gene disruption on fungal growth under different culture conditions. Log₂(Δ*rkp*/Control) values were calculated for mycelial content in solid-state culture (rice-*koji*), dry cell weight in liquid culture, and colony diameter on agar plates. These values are shown as a heatmap to illustrate the impact of *rkp* gene disruption on fungal growth under varying conditions.

Mycelial content in rice-*koji*, dry biomass in liquid culture, and colony size on agar medium were evaluated and compared between each Δ*rkp* strain and the control strain (Fig. 3; Fig. S1 and Table S3). Growth data for Δ*rkp*010 are not shown because this strain could not be subjected to rice-*koji* preparation and subsequent small-scale sake brewing under the experimental conditions used. In rice-*koji*, although some Δ*rkp* strains exhibited altered mycelial content, the overall variation was relatively limited, with most changes reflecting reduced growth. In liquid culture, in addition to strains showing reduced biomass, some also displayed increased biomass. Notably, Δ*rkp*005, Δ*rkp*024, Δ*rkp*064, Δ*rkp*082, Δ*rkp*089, Δ*rkp*112, Δ*rkp*135, Δ*rkp*162, and Δ*rkp*175 showed markedly reduced biomass. Compared with the effects on mycelial content observed in rice-*koji*, the impact on growth was generally more pronounced under liquid culture conditions. Many heterokaryotic disruptants exhibited reduced growth in both rice-*koji* and liquid culture. In contrast, colony size on agar medium was largely unaffected, with only one strain, Δ*rkp*175, showing a noticeable deviation from the control.

### Effect of *rkp* gene disruption on rice-*koji* and sake

Small-scale sake brewing was performed using rice-*koji* prepared from each Δ*rkp* strain. In total, 84 Δ*rkp* strains were tested, excluding one strain (Δ*rkp*010) that showed severely impaired growth on steamed rice. Analyses of the rice-*koji* included measurements of mycelial content, total protein, and the activities of four key enzymes essential for sake brewing—α-amylase, glucoamylase, acid carboxypeptidase, and acid protease. For the resulting sake, we monitored CO₂ evolution during fermentation, determined general chemical properties (ethanol concentration, acidity, amino acid content, and *nihonshu-do*), measured sake cake weight, and analyzed free amino acid profiles, organic acid composition, and major aroma components. All results were expressed as relative values to the control strain and are listed in Tables S3 and S4.

**Fig 4:**
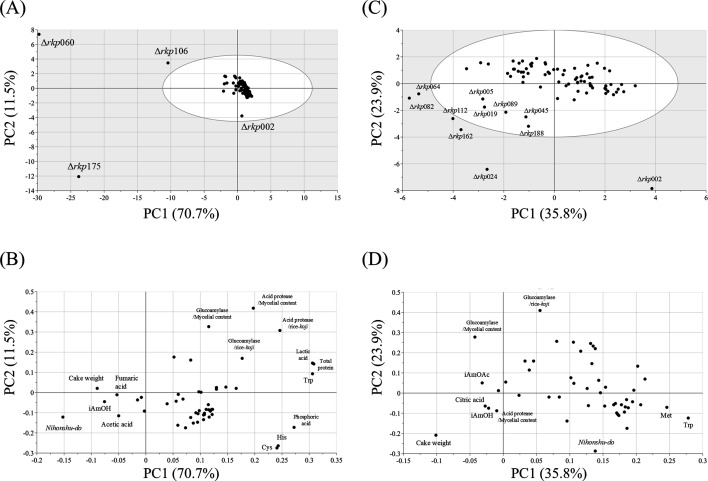
PCA of rice-*koji* and sake prepared using Δ*rkp* strains.(**A**) Score plot of PCA using general analytical results from rice-*koji* and sake. (**B**) Loading plot corresponding to panel A. (**C**) Score plot of PCA excluding Δ*rkp*060, Δ*rkp*106, and Δ*rkp*175. (**D**) Loading plot corresponding to panel C.

Among the Δ*rkp* strains with reduced mycelial content in rice-*koji*, a general trend toward lower enzymatic activities was observed. Although some exceptions were noted, these strains typically exhibited decreased CO₂ release during fermentation and reduced ethanol concentrations in the resulting sake. Given the substantial variation across Δ*rkp* strains in both rice-*koji* and sake parameters, principal component analysis (PCA) was performed to visualize the overall distribution patterns, assess similarities among strains, and identify major trends (Fig. 4A and B). Principal component 1 (PC1) accounted for 70.7% of the total variance, and together with PC2, explained 72.8% of the variance. In the PCA score plot (Fig. 4A), Δ*rkp*060, Δ*rkp*106, and Δ*rkp*175 displayed markedly different profiles compared with the other Δ*rkp* strains. Δ*rkp*060, Δ*rkp*106, and Δ*rkp*175 were separated primarily along PC1. The corresponding loading plot (Fig. 4B) indicated that *nihonshu-do*, sake cake weight, and isoamyl alcohol content contributed predominantly to the negative direction of PC1. These variables were associated with the separation of the three Δ*rkp* strains (Δ*rkp*060, Δ*rkp*106, and Δ*rkp*175), suggesting that differences in these parameters were major contributors to their distinct profiles in the PCA score plot.

Because the four Δ*rkp* strains mentioned above differed markedly from the others—due to poor fungal growth and pronounced negative effects on fermentation—we conducted a second PCA excluding these strains (Fig. 4C and D). In this analysis, PC1 and PC2 explained 35.8% and 23.9% of the total variance, respectively, accounting for 59.7% in total. Most Δ*rkp* strains formed a cluster centered around the origin and were broadly distributed along the PC1 axis (Fig. 4C). In contrast, several strains were widely distributed toward the third quadrant, and Δ*rkp*002 was plotted separately in the fourth quadrant. Many of the strains distributed in the third quadrant were obtained only as heterokaryotic disruptants. According to the corresponding loading plot (Fig. 4D), sake cake’s weight contributed strongly to the separation toward the third quadrant.

### Effect of *rkp* gene disruption on the metabolomic composition of sake

In addition to the general component analysis of sake, metabolomic profiling was performed to evaluate the effects of each Δ*rkp* strain on the composition of the resulting sake. While general component analysis can reveal changes in key brewing parameters, sake contains a highly complex mixture of compounds, and many metabolic alterations cannot be captured through routine measurements. Therefore, we applied the sake metabolome analysis method to sake brewed with each Δ*rkp* strain. This analysis yielded a peak table containing 11,386 peaks. Based on the coefficient of variation, 1,423 metabolite peaks were selected for further analysis. Relative abundances compared with the control strain, along with putative compound annotations, are provided in Table S5.

**Fig 5:**
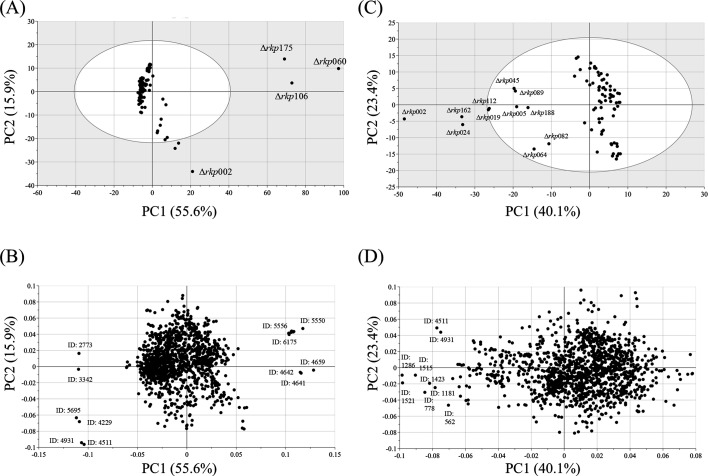
PCA of metabolomic data from sake brewed using Δ*rkp* strains. (**A**) Score plot of PCA based on metabolomic data from sake samples. (**B**) Loading plot corresponding to panel A. (**C**) Score plot of PCA excluding Δ*rkp*060, Δ*rkp*106, and Δ*rkp*175. (**D**) Loading plot corresponding to panel C.

These results further confirmed that the three Δ*rkp* strains—Δ*rkp*002, Δ*rkp*060, Δ*rkp*106, and Δ*rkp*175—exhibited markedly distinct profiles from the other strains in both general and metabolomic analyses. PCA was performed using the metabolomic data set (Fig. 5A and B). PC1 explained 55.6% of the total variance and, together with PC2, accounted for 71.5%. Consistent with the PCA results based on general sake parameters, the three Δ*rkp* strains were clearly separated from the others in the score plot (Fig. 5A). For the three Δ*rkp* strains separated along the positive direction of PC1, several metabolite peaks were suggested to contribute to this separation, including peak IDs: 5550, 5556, 6175, 4659, 4641, and 4642. Among these peaks, ID: 5550, 5556, and 6175 were detected exclusively in the three Δ*rkp* strains separated along PC1. In contrast, peak ID: 4659, 4641, and 4642 were detected only in these three strains together with Δ*rkp*002 and were not detected in the remaining Δ*rkp* strains and control strain.

To further explore the characteristics of the remaining Δ*rkp* strains, PCA was repeated using the metabolomic data with these three outlier strains excluded (Fig. 5C and D). In this analysis, PC1 and PC2 accounted for 40.1% and 23.4% of the variance, respectively, totaling 63.5%. In the score plot (Fig. 5C), 11 Δ*rkp* strains—Δ*rkp*002, Δ*rkp*005, Δ*rkp*019, Δ*rkp*024, Δ*rkp*045, Δ*rkp*064, Δ*rkp*082, Δ*rkp*089, Δ*rkp*112, Δ*rkp*162, and Δ*rkp*188—were clearly separated along the positive direction of PC1. Notably, 8 of these 11 were heterokaryotic disruptants, representing the majority of the total 12 heterokaryotic strains. These strains corresponded precisely to those previously distributed toward the third quadrant in Fig. 4C. The remaining Δ*rkp* strains were broadly distributed along the PC2 axis. The loading plot (Fig. 5D) indicated that several peaks strongly contributing to PC1 separation lacked putative compound annotations. Examination of the retention times of these peaks allowed the corresponding chromatographic features to be examined, and subsequent mass spectral analysis revealed multiple fragment ions characteristic of sugar-derived units. Based on these spectral features, these peaks were classified as sugar-related structures, such as oligosaccharide- or polysaccharide-derived components.

### Characterization of significantly altered metabolomic peaks in sake brewed with Δ*rkp* strains

**Fig 6:**
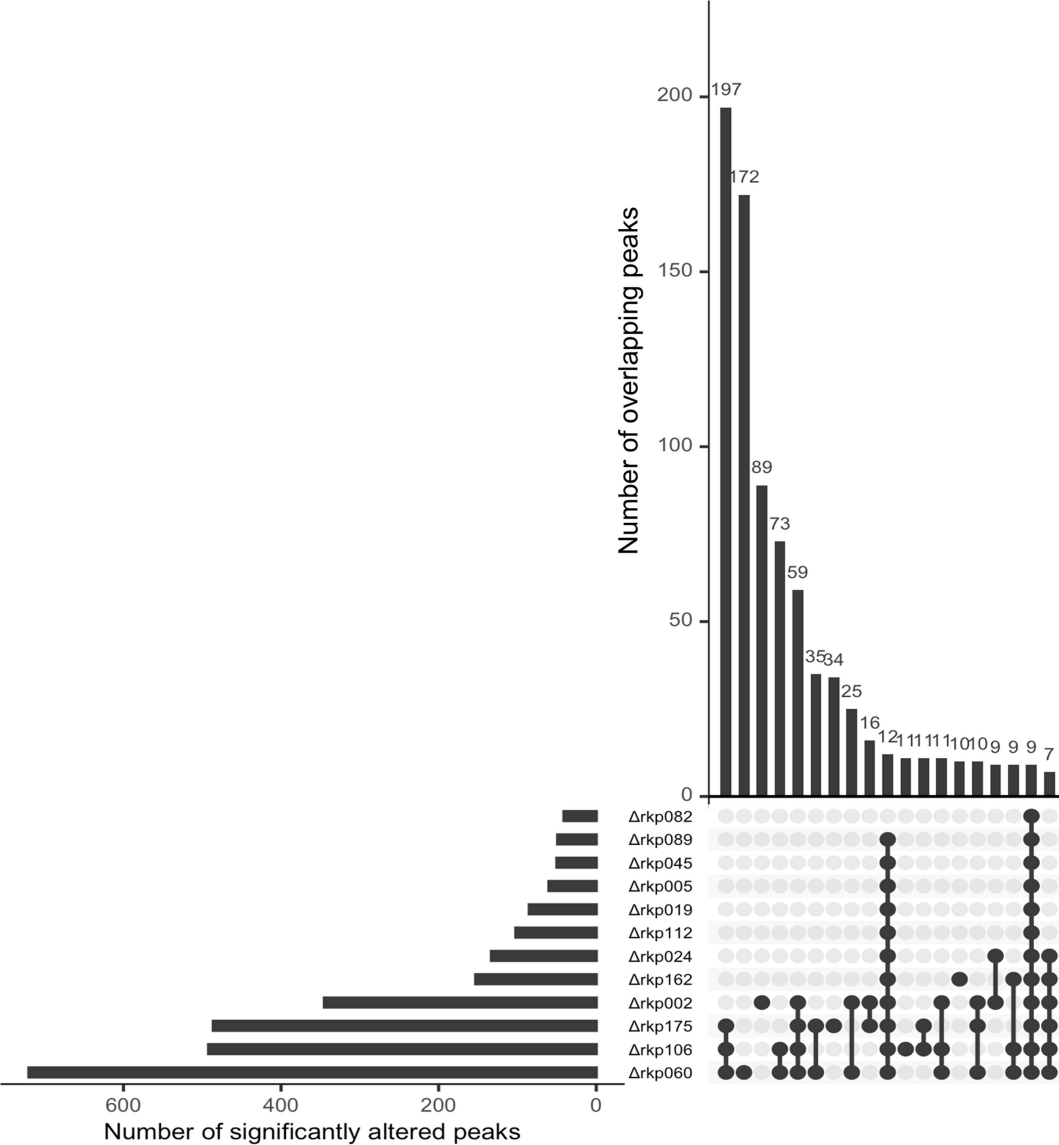
Significantly altered peak patterns in Δ*rkp* strains with elevated peak counts. An UpSet plot was used to examine the overlap of significantly altered peaks among 12 Δ*rkp* strains with the highest numbers of such peaks. The horizontal bar graph on the left indicates the number of significantly altered peaks identified in each Δ*rkp* strain. The central dot matrix represents the combinations of strains for which shared peaks were identified. The vertical bar graph above shows the number of significantly altered peaks shared among each corresponding combination of strains.

We focused on individual metabolomic peaks that exhibited significant intensity changes as a result of *rkp* gene disruption. For each sake sample, “significantly altered peaks” were defined as those showing a fold change of ≥2 compared with the control strain and a *P*-value < 0.01 by Student’s *t*-test. The number of such peaks was counted for each sample. Among the 84 Δ*rkp* strains, no significantly altered peaks were detected in sake brewed with 27 strains. In contrast, the remaining 57 strains showed between 1 and 720 significantly altered peaks, indicating substantial variability in the extent of metabolic changes depending on the disrupted gene. While most sake samples contained fewer than 25 significantly altered peaks, 12 Δ*rkp* strains produced sake with 40 or more such peaks, suggesting a particularly broad impact on metabolomic composition (Fig. 6). Notably, most of these 12 strains corresponded to those with reduced mycelial content in rice-*koji* or markedly distinct profiles in the PCA results. Specifically, they included the three Δ*rkp* strains with highly divergent characteristics—Δ*rkp*060, Δ*rkp*106, and Δ*rkp*175—as well as 9 of the 11 strains clearly separated in Fig. 5C.

An UpSet plot was used to examine the overlap of significantly altered peaks among the 12 Δ*rkp* strains with the highest numbers of such peaks (Fig. 6). The plot summarizes the number of overlapping and unique peaks across different combinations of strains, with the top 19 combinations presented. The three Δ*rkp* strains with highly divergent profiles (Δ*rkp*060, Δ*rkp*106, and Δ*rkp*175) and Δ*rkp*002 exhibited particularly large numbers of significantly altered peaks. Most of these peaks overlapped with those found in at least one of the other 12 strains. Among the nine peaks significantly altered across all 12 strains, 7 corresponded to peaks that contributed to the PC1 separation shown in Fig. 5D. Although only partial results are displayed, strain-specific altered peaks were also identified: 172 in Δ*rkp*060, 89 in Δ*rkp*002, 34 in Δ*rkp*175, 11 in Δ*rkp*106, 10 in Δ*rkp*162, 3 in Δ*rkp*082, and 1 in Δ*rkp*112. These peaks likely reflect strain-specific effects of gene disruption that are independent of general growth defects in rice-*koji* or overall fermentation performance.

By contrast, for Δ*rkp* strains in which fewer than 25 significantly altered peaks were detected, the impact of gene disruption on sake composition appeared to be relatively limited. For these strains, we therefore focused on the individual significantly altered peaks observed in each sample, with particular attention to those peaks for which putative compound annotations could be inferred. As a result, several putative compounds, including agmatine, ferulic acid, and hypoxanthine, were suggested based on the characteristics of the significantly altered peaks. Notably, among the Δ*rkp* strains examined, significant changes in peaks putatively assigned to ferulic acid were observed exclusively in the Δ*rkp*171 strain.

## DISCUSSION

In this study, we comprehensively characterized protein production by *A. oryzae* during rice-*koji* preparation was assessed, and the effects of gene disruptions on growth, enzyme activity, and overall sake composition were examined. In addition, metabolomic profiling of sake was carried out to detect metabolite peaks influenced by *rkp* gene disruptions, thereby clarifying the roles of *A. oryzae* genes in shaping sake chemistry.

Proteomic analysis was performed on four rice-*koji* types: Nipponbare-RIB128-Shubosoe, Nipponbare-RIB128-Nakatome, Yamadanishiki-RIBOIS01-Shubosoe, and Yamadanishiki-RIBOIS01-Nakatome (Fig. 1; Table S2). The Nipponbare-RIB128 rice-*koji* represented production for standard-grade sake brewing, whereas Yamadanishiki-RIBOIS01 reflected ginjo-grade brewing practices. Substantial differences in protein profiles were observed according to rice-*koji* grade (i.e., rice cultivar and *A. oryzae* strain), while comparatively minor differences occurred between samples with different intended brewing applications (Table S2). Four proteins were uniquely detected under each cultivar-strain condition. For Nipponbare-RIB128, these included RKP005 (Enoyl-CoA hydratase), RKP032 (Tyrosinase), RKP059 (homogentisate 1,2-dioxygenase), and RKP328 (aldo/keto reductase family protein). In contrast, Yamadanishiki-RIBOIS01 uniquely contained RKP220 (predicted protein), RKP223 (predicted protein), RKP288 (WD40 repeat), and RKP351 (fructose 1,6-bisphosphate aldolase [FbaA]). No functional linkage was evident among the four proteins in either group. Among these, RKP059 and RKP351 were assigned to identity groups B and A, respectively, based on predicted functions inferred from sequence homology with characterized proteins in other species. In particular, RKP351 encodes FbaA, previously reported in *A. oryzae* (12). In *Aspergillus nidulans*, FbaA is known to be essential for fungal growth (13). Although the *rkp*351 gene sequence is fully conserved in the genomes of both strains examined here, the corresponding protein was not detected in the Nipponbare-RIB128 rice-*koji* sample. Data from the CAoGDX database (https://nribf21.nrib.go.jp/CAoGDX/) indicate that *rkp*351 is constitutively and strongly expressed during rice-*koji* production in the laboratory strain *A. oryzae* RIB40. These observations suggest that, depending on the combination of fungal strain and rice cultivar, the regulation or accumulation of FbaA at the protein level may differ under rice-*koji* cultivation conditions.

Among the resulting disruptants, 12 were obtained as heterokaryons. For the 40 *rkp* genes where disruptants could not be obtained, as well as the 12 genes yielding only heterokaryotic disruptants, gene disruption may severely impair growth or viability, suggesting that these genes play important roles in fungal physiology. Among the *rkp* genes lacking disruptants, *rkp*094 was classified into group A and, based on annotation, is predicted to encode a thioredoxin reductase. In *Aspergillus fumigatus*, TrxR—which encodes thioredoxin reductase—is essential for redox homeostasis (14). Similarly, *rkp*133, for which no disruptants were obtained, is predicted to encode a subtilisin-related protease/vacuolar protease B. This enzyme has been reported in *A. fumigatus*, where its deletion caused a modest reduction in growth rate and a pronounced decrease in conidiation efficiency (15). The amino acid sequences encoded by these genes exhibited over 85% similarity to counterparts in other *Aspergillus* species, supporting the likelihood of conserved functions. These observations suggest that the absence of disruptants for certain *rkp* genes may stem from their roles in primary metabolism or conidiation. We also assessed the predicted functions of *rkp* genes for which only heterokaryotic disruptants were recovered. *rkp*019 encodes a protein with high sequence homology to orthologs in other species and is annotated as a molecular chaperone of the heat shock protein 70 (HSP70) family. HSP70 proteins, ranging from 66 to 78 kDa, are highly conserved from archaea to humans. In *S. cerevisiae*, the HSP70 family contains at least 10 members, several of which are essential for viability (16, 17). Although no studies on HSP70 function in *A. oryzae* have been reported to date, their strong conservation across diverse species suggests they play essential roles in fungal physiology. *rkp*024, also predicted to encode an HSP70 family member, was similarly recovered only in heterokaryotic form after disruption. In addition, *rkp*047 was predicted to belong to the HSP60 family, indicating that multiple HSPs are represented among the RKPs. However, research specifically addressing HSPs in *A. oryzae* remains scarce (18). Collectively, the 52 *rkp* genes for which disruptants could not be obtained or were recovered solely as heterokaryons likely include genes essential for *A. oryzae* physiology. Of these, 46 were assigned to identity groups B and C, and their functions remain largely uncharacterized. In the KOG classification, some RKPs mapped to multiple categories, whereas 83 were designated as “function unknown” (Fig. 2). Among the functionally classified RKPs, many were linked to amino acid and carbohydrate transport/metabolism, as well as protein modification and turnover, underscoring their potential physiological importance in *A. oryzae*. Further characterization is warranted to clarify their specific contributions to rice-*koji* production.

In this study, we examined various brewing parameters of sake produced using *rkp* gene disruptants to assess the impact of *rkp* genes, identified through proteomic analysis of rice-*koji*, on sake composition. Notably, metabolomic analysis offers a non-targeted approach, enabling comprehensive profiling even when the key sample characteristics are unknown, as in this work. The sake metabolome analysis method applied here has been validated for sake samples (1) and also for brown rice, the raw material for sake, where it has been used to predict brewing characteristics from rice properties (2). We evaluated multiple parameters related to rice-*koji*, fermentation, and the general composition of the resulting sake in combination with metabolomic data. This integrated analysis revealed that the overall characteristics of sake derived from the *rkp* disruptants were strongly influenced by the extent of fungal growth in rice-*koji*. PCA showed that disruptants with substantially reduced fungal biomass—caused by disruption of certain *rkp* genes—clustered separately in the PCA plot, primarily along variables such as sake cake weight and *nihonshu-do* (Fig. 4). These trends likely reflect impaired starch saccharification in rice-*koji* due to poor fungal growth, leading to incomplete rice degradation, insufficient glucose release, subsequent effects on *nihonshu-do*, and residual cake weight. Furthermore, compounds such as isoamyl acetate, isoamyl alcohol, and various amino acids contributed to the PCA separation of *rkp* disruptants. These associations may reflect interactions between amino acid release from protein degradation in rice-*koji* and the formation of aroma compounds by yeast. Although reduced fungal growth in rice-*koji* emerged as a major factor contributing to the separation of *rkp* disruptants in PCA, strains showing poor growth did not exhibit uniform changes across all enzymatic and compositional parameters. While many parameters tended to deteriorate in growth-impaired strains, the extent and pattern of these changes varied among disruptants. This indicates that, even within growth-deficient phenotypes, different factors may differentially contribute to sake fermentation characteristics. Therefore, the PCA results in this study should be interpreted as providing an integrated overview of dominant trends rather than definitive functional assignments for individual *rkp* genes. Identifying which specific parameters within growth-related phenotypes most strongly influence sake quality will require further targeted analyses.

Analysis of metabolite peaks significantly altered by *rkp* disruption also suggested links between specific gene disruptions and the corresponding metabolite changes. Among these, *rkp*171, annotated as endo-1,4-β-xylanase B, was particularly notable, with 13 significantly altered metabolite peaks detected in sake brewed using the Δ*rkp*171 strain. Two of these peaks, putatively identified as ferulic acid, showed markedly reduced intensities compared with the control strain: 0.387-fold (peak ID: 1465) and 0.420-fold (peak ID: 1647), respectively. Ferulic acid has been reported to exhibit diverse bioactivities in animal models, including antihypertensive, cholesterol-lowering, and hypoglycemic effects (19), and contributes to sourness, astringency, and freshness in sake flavor (20). In rice cell walls, ferulic acid occurs as a side chain bound via β-1,4-linkages to xylan, a hemicellulose component. Although phenolic compounds are generally more abundant in less-polished rice, previous studies have reported that phenolic compounds can still be detected in rice-*koji* extracts prepared from highly polished rice (21). While these components are present at lower levels, their modification during rice-*koji* cultivation has not been extensively discussed. The enzymatic release of ferulic acid from hemicellulose requires the combined action of xylanases and accessory enzymes such as ferulic acid esterases (22). Xylanases in *A. oryzae* have been extensively studied, with reported genes including four from glycoside hydrolase (GH) family 10 (*xynF1–4*) and two from GH family 11 (*xynG1* and *xynG2*) (23–25). The CAZy database (http://www.cazy.org) also lists four GH10 and four GH11 genes in *A. oryzae*. Additionally, 13 ferulic acid esterases have been structurally classified in this species (26, 27). *rkp*171 is predicted to encode a GH11 family xylanase, distinct from the known *xynG1* and *xynG2*. Among other *rkp* genes, *rkp*157 and *rkp*186 are predicted to encode *xynF3* and *xynG1*, respectively. In *A. niger*, expression of ferulic acid esterases is induced by both xylose and aromatic compounds such as ferulic acid (28, 29). Taken together, these findings suggest that *rkp*171 likely encodes a previously uncharacterized xylanase, and its disruption may reduce the release of both xylose and ferulic acid. Although *rkp*157 and *rkp*186 were also expressed xylanases, their contribution to ferulic acid release appears limited. In *awamori*, a traditional Japanese distilled beverage, vanillin—the characteristic aroma compound—is derived from ferulic acid via decarboxylation to 4-vinylguaiacol (4-VG), catalyzed by enzymes from black *koji* molds (30). By contrast, 4-VG is generally regarded as an off-flavor in sake and has been reported to originate from rice-*koji* (31). These observations highlight the dual significance of ferulic acid, both as a flavor component and as a precursor of other aroma compounds. Modulating ferulic acid release could therefore provide a means to adjust the flavor profile and characteristics of fermented beverages.

In this study, we identified RKPs produced in rice-*koji* used for traditional Japanese sake brewing and demonstrated their potential contributions to sake chemistry and flavor through an integrated approach combining comprehensive proteomic analysis, targeted gene disruption, and untargeted metabolomic profiling of the final product. Further functional studies of individual RKPs may enable deliberate modulation of specific sake compositions during brewing. Moreover, many RKPs include physiologically important proteins in *A. oryzae*, most of which remain functionally uncharacterized. Elucidating their roles will not only enhance our understanding of sake brewing but also provide new insights into the physiological significance of unexplored proteins in filamentous fungi.

## MATERIALS AND METHODS

### Strains and media

For proteomic analysis of rice-*koji*, two *A. oryzae* strains, RIB128 and RIBOIS01, both maintained at the National Research Institute of Brewing (NRIB) in Hiroshima, were used. To construct gene-disruption strains, *A. oryzae* NSR-*ΔlD2* (*niaD⁻*, *sC⁻*, *adeA⁻*, and *ΔligD*), derived from the genome-sequenced strain RIB40, served as the host strain (32). For experiments involving gene-disruption strains, an *adeA*-complemented derivative of *A. oryzae* NSR-ΔlD2 (*niaD⁻*, *sC⁻*, and *ΔligD*) was used as the control. Fungal strains were grown on potato dextrose agar and glucose minimal medium (Table S6) at 30°C under saturated-humidity conditions. The NSR-*ΔlD2* strain was cultured on glucose minimal medium supplemented with adenine, L-glutamic acid as the nitrogen source, and L-methionine as the sulfur source, without nitrate. For the cultivation of gene-disruption strains and the *adeA*-complemented control, the same medium lacking adenine was employed. Detailed procedures for rice-*koji* cultivation and assessment of the effects of gene disruption on fungal growth are provided in a separate section.

### Analysis of proteome in rice-*koji*

#### Rice-*koji* preparation

To examine proteomic differences under contrasting rice-*koji* conditions relevant to sake brewing, proteomic analysis was performed on four rice-*koji* types selected as representative model conditions. Two rice cultivars and *A. oryzae* strains were employed: standard-*koji* was prepared from the Nipponbare cultivar (polishing ratio: 70%) inoculated with *A. oryzae* RIB128, while ginjo-*koji* was produced from the Yamadanishiki cultivar (polishing ratio: 40%) inoculated with *A. oryzae* RIBOIS01. For each combination, two inoculation levels were applied to simulate conditions for Shubosoe-*koji* (used in yeast starter preparation) and Nakatome-*koji* (used in main mash fermentation), yielding four distinct rice-*koji* samples.

Rice-*koji* was produced at a pilot-scale brewery facility using approximately 30–40 kg of polished rice. Polished rice was soaked overnight in water to ensure adequate hydration and then steamed for 50 min in a *koshiki* steamer. The steamed rice was cooled to 35°C before inoculation. Conidia were distributed evenly over the rice to promote uniform fungal growth. After 12 h of incubation, the first mixing (*kirikaeshi*) was performed, adjusting the temperature to 32°C. Another 10-12 h later, when the temperature reached 34°C–36°C, the second mixing and reshaping (*mori*) were conducted to bring it back to 32°C. When the temperature rose to 36°C–37°C, a third mixing (*nakashigoto*) was carried out. Three to four hours afterward, at 39°C–40°C, the final mixing (*shimaishigoto*) was performed. Approximately 12 h later, once the temperature reached 42°C, the rice-*koji* was harvested (*dekouji*). The harvested rice-*koji* was immediately frozen in liquid nitrogen and stored at −80°C until analysis.

#### Proteome analysis by 2-DE and mass spectrometry

Proteome analysis of rice-*koji* was conducted following the method of Oda et al. (11). Crude protein extracts were prepared by homogenizing rice-*koji* samples with a tissue homogenizer, followed by trichloroacetic acid (TCA)/acetone precipitation to obtain the final extracts. A total of 250 µg of protein was separated by two-2-DE. The resulting gels were imaged using a ProXPRESS Proteomic Imaging System (PerkinElmer), and protein spot patterns were analyzed with ProFinder v2005 software (COGNEX). Selected protein spots were excised and analyzed by PMF and MS/MS on an Ultraflex TOF/TOF mass spectrometer (Bruker Daltonics). Peptide data were searched against protein databases using Mascot (http://www.matrixscience.com) for identification. Functional annotation of RKPs was carried out using the KOG database to classify putative functions, and these annotations were cross-referenced with CAoGDX (https://nribf21.nrib.go.jp/CAoGDX/).

### *rkp* gene disruption

Targeted disruption of *rkp* genes was performed using the protoplast-polyethylene glycol-mediated transformation method (33). *A. oryzae* NSR-ΔlD2 (*niaD*⁻, *sC*⁻, *adeA*⁻, and Δ*ligD*) served as the host strain, and the *adeA* gene was used as the selectable marker. Disruption cassettes were assembled by fusion PCR, combining three DNA fragments: the 5′ and 3′ flanking regions of the target gene and the *adeA* marker fragment (34). Primer sequences used for construction of the gene disruption fragments are listed in Table S7. Gene replacement was achieved via homologous recombination using the assembled cassette. For each *rkp* gene, two or three independent disruptant strains were obtained and used as biological replicates in subsequent analyses.

#### Rice-*koji* preparation with *rkp* disruptants

For small-scale cultivation, gelatinized rice prepared from 70% polished *Akihikari* was used. The rice was dried at 90°C for 2 h, portioned into 15 g samples in 90 mm plastic Petri dishes, and dried again at 60°C for 1 h. Each strain was inoculated with a conidial suspension to reach an initial spore concentration of 1 × 10⁶ conidia/g gelatinized rice and a starting moisture content of 30%. Plates were incubated at 35°C under saturated humidity for 42 h. Following cultivation, 5 g of rice-*koji* was collected into tubes and stored at −30°C until further use. For each *rkp* gene, rice-*koji* preparation was performed using two or three independently obtained disruptant strains under identical cultivation conditions.

### Growth phenotype analysis of *rkp* disruptants

Colony diameter and conidiation rate were evaluated on agar plates, while dry cell weight was measured in liquid culture, and mycelial content was assessed in solid-state culture using steamed rice. For colony and conidiation assays, 1 μL of a conidial suspension (1 × 10⁵ conidia in 30% glycerol) was inoculated onto glucose minimal agar medium (Table S6) supplemented with L-glutamic acid as the nitrogen source and L-methionine as the sulfur source, without nitrate. Cultures were incubated at 30°C under saturated humidity for 5 days. Conidiations were harvested from colonies grown on agar plates using a solution of 0.025% (vol/vol) Tween 80 and 0.5% (wt/vol) NaCl, and the number of conidia was determined using a Thoma hemocytometer. For measurement of dry cell weight in liquid culture, 1 × 10⁸ conidia were inoculated into 100 mL of glucose minimal medium and cultivated at 30°C with shaking at 100 rpm for 24 h. The resulting mycelia were collected and dried in an oven at 105°C for 1 h, and the dry weight was measured. The mycelial content of rice-*koji* can be quantified based on measurement of the amount of *N*-acetylglucosamine (GlcNAc) produced upon enzymatic lysis of rice-*koji* (35). The amount of GlcNAc in mycelia obtained from liquid culture and rice-*koji* was quantified using a previously reported method (36).

### Enzymatic activity in rice-*koji* prepared using *rkp* disruptants

Enzyme activities in rice-*koji* samples were determined according to the official methods of the National Tax Administration Agency, Japan, and the standard analytical methods of the NRIB, Japan (37, 38). Acid protease activity was measured using the official protocol, whereas α-amylase, glucoamylase, α-glucosidase, and acid carboxypeptidase activities were measured using assay kits (Kikkoman Biochemifa, Tokyo, Japan) in accordance with these standard methods.

### Laboratory-scale sake brewing and sake analysis

Laboratory-scale sake brewing was performed with rice-*koji* prepared from each Δ*rkp* strain. Sake brewing experiments were conducted using rice-*koji* prepared from two or three independently obtained disruptant strains for each *rkp* gene, allowing comparison at the biological replicate level under standardized brewing conditions. Brewing employed 80 g of total rice (including rice-*koji*) and the yeast strain K701, following a previously described small-scale sake production method (1). After fermentation, the mash was centrifuged at 4,000 × *g* for 10 min, and the supernatant was collected as the refined sake. Alcohol concentration, *nihonshu-do* (sake meter value), acidity, and amino acid content were analyzed using the official methods of the National Tax Administration Agency, Japan (37).

### Metabolome analysis of sake

Metabolome analysis of sake was performed using a previously established sake metabolomics method (1). Ultra-performance liquid chromatography-quadrupole time-of-flight mass spectrometry (UPLC-Q/TOF-MS) was conducted with a Xevo G2 Q/TOF-MS system coupled to an Acquity UPLC system (Waters, Milford, MA, USA). Metabolites were separated on an Acquity UPLC HSS T3 column (2.1 × 150 mm, 1.8 μm; Waters). The mobile phases consisted of (A) Milli-Q water with 0.1% (vol/vol) formic acid and (B) acetonitrile with 0.1% (vol/vol) formic acid. Gradient elution was programmed as follows: 0 min, 0% B; 5 min, 0% B; 15 min, 100% B; 20 min, 100% B; 21 min, 0% B; and 30 min, 0% B. Electrospray ionization was applied in positive ion mode. Instrument settings were capillary voltage, 2 kV; cone voltage, 15 V; source temperature, 140°C; desolvation temperature, 450°C; cone gas flow rate, 50 L/h; and desolvation gas flow, 800 L/h. Data were acquired in MS 1 mode over an *m/z* range of 50–1,000 at five scans per second. Each sample was analyzed in triplicate.

### Data processing

MS peak alignment, peak picking, and intensity normalization were carried out using Progenesis QI v2.4 (Nonlinear Dynamics, Newcastle upon Tyne, UK). The Progenesis QI settings were as follows: data format, centroided (resolution: 9,000); ion mode, positive; and only the [M + H]^+^ adduct was included. Peak picking was performed using the automatic processing algorithm with the sensitivity set to “> 3.” The minimum chromatographic peak width allowed during peak picking was 0.05 min. For normalization, the total ion intensity of all detected features in each sample served as the reference. After normalization, a peak table was generated and used for preliminary data processing. The tolerances for mass and retention time deviations were set to 0.05 Da and 0.3 min, respectively. To define the threshold for peak inclusion in subsequent statistical analyses, a model sake containing 14 representative sake components was analyzed. The coefficients of variation (CVs) of these components were calculated, and the maximum CV value among them was used as a reference. Peaks with CV values within three times this maximum CV were retained for further analysis.

### Statistical analysis

PCA of rice-*koji* analytical results, sake general properties, and sake metabolomic data were performed using SIMCA ver. 16.0.2 (Umetrics, Umeå, Sweden). All data sets were preprocessed by calculating ratios relative to the control strain and subsequently applying log transformation to these relative ratios. PCA was then conducted after mean-centering and Pareto scaling.

## SUPPLEMENTAL MATERIAL

Figure S1 aem.01955-25-s0001.pdf Colony morphology and conidiation of the control strain and Δ*rkp* strains.

Table S1 aem.01955-25-s0002.pdf List of constructed *rkp* gene disruptants.

Table S2 aem.01955-25-s0003.pdf List of protein spots identified by proteomic analysis.

Table S3 aem.01955-25-s0004.pdf Summary of analytical results for rice-*koji* using Δ*rkp* strains.

Table S4 aem.01955-25-s0005.pdf Summary of analytical results for sake produced using Δ*rkp* strains.

Table S5 aem.01955-25-s0006.xlsx Summary of metabolome analytical results for sake produced using Δ*rkp* strains.

Table S6 aem.01955-25-s0007.pdf Composition of glucose minimal medium.

Table S7 aem.01955-25-s0008.pdf Primers used for generation of gene disruption fragments.

ASM does not own the copyrights to Supplemental Material that may be linked to, or accessed through, an article. The authors have granted ASM a non-exclusive, world-wide license to publish the Supplemental Material files. Please contact the corresponding author directly for reuse.
