# Supplementary material for: Effects of Aspergillus oryzae-derived rice-koji protein on the sake metabolome
Source: Appl Environ Microbiol. 2026 Feb 19;92(3):e01955-25. doi: 10.1128/aem.01955-25 (PMC12997762; doi:10.1128/aem.01955-25)
Supplement: Figure S1 — Colony morphology and conidiation of the control strain and Δrkp strains. [file aem.01955-25-s0001.pdf]

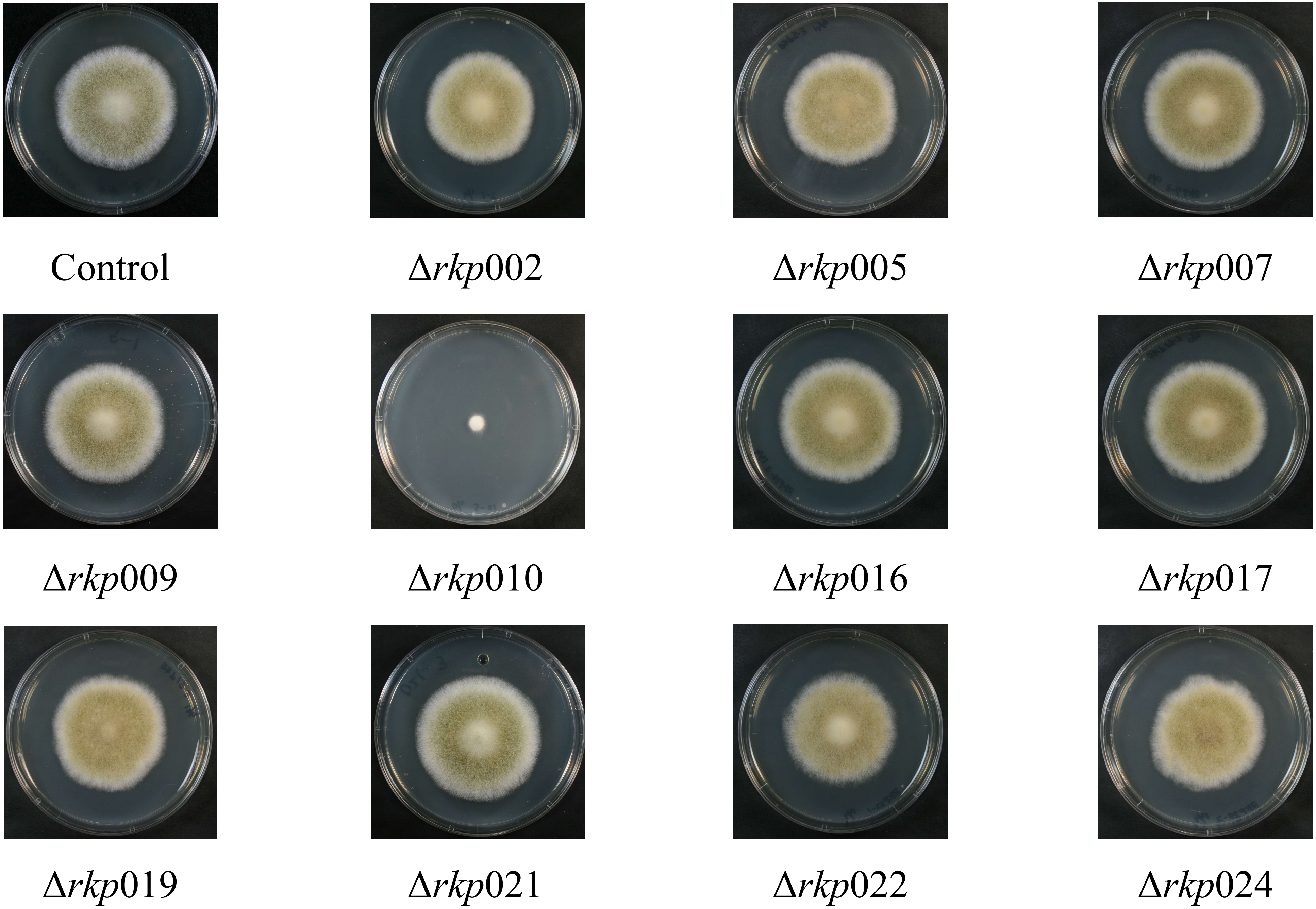
Control
Δ*rkp*002
Δ*rkp*005
Δ*rkp*007
Δ*rkp*009
Δ*rkp*010
Δ*rkp*016
Δ*rkp*017
Δ*rkp*019
Δ*rkp*021
Δ*rkp*022
Δ*rkp*024

Fig. S1

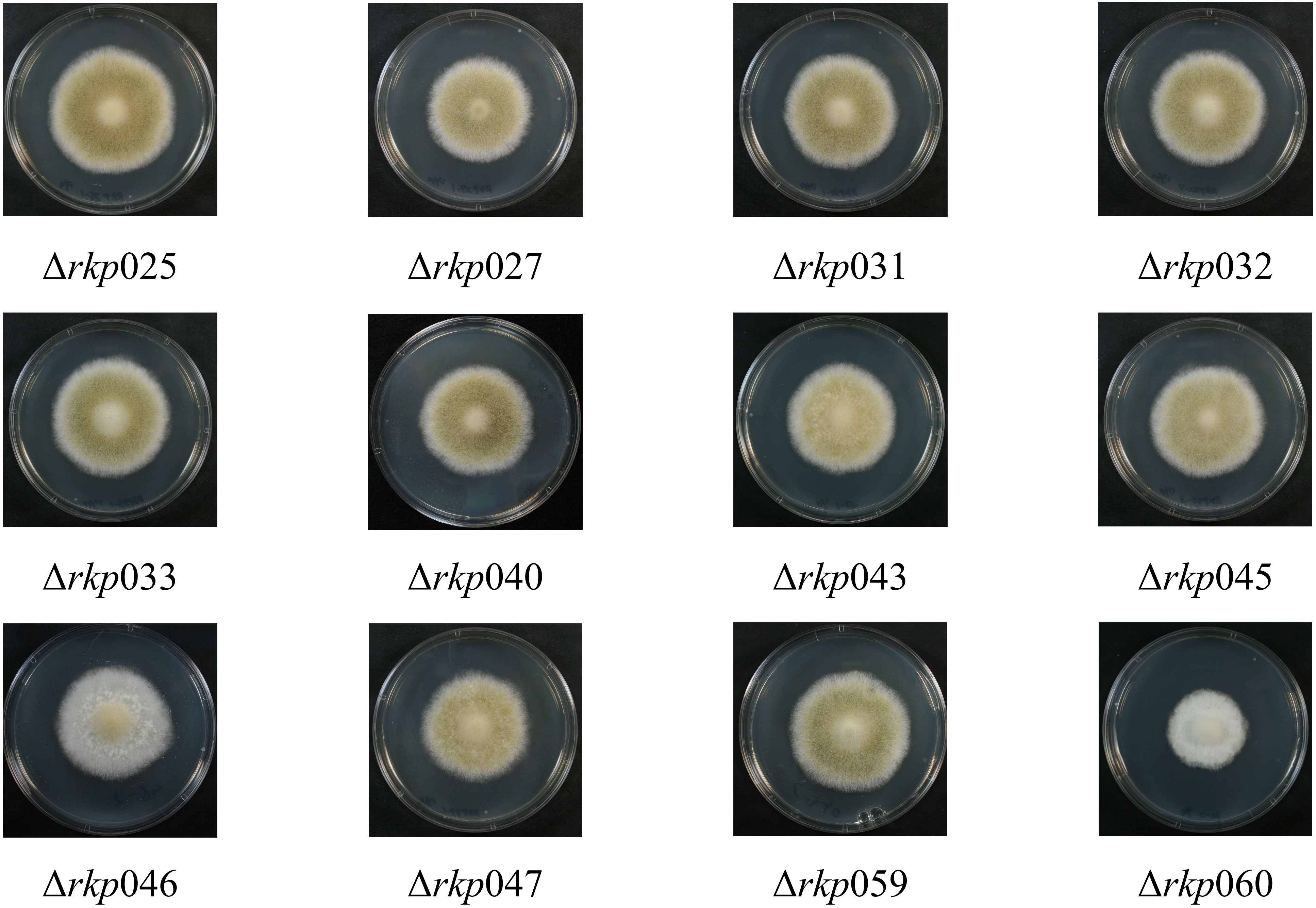
Δ*rkp*025
Δ*rkp*027
Δ*rkp*031
Δ*rkp*032
Δ*rkp*033
Δ*rkp*040
Δ*rkp*043
Δ*rkp*045
Δ*rkp*046
Δ*rkp*047
Δ*rkp*059
Δ*rkp*060

Fig. S1

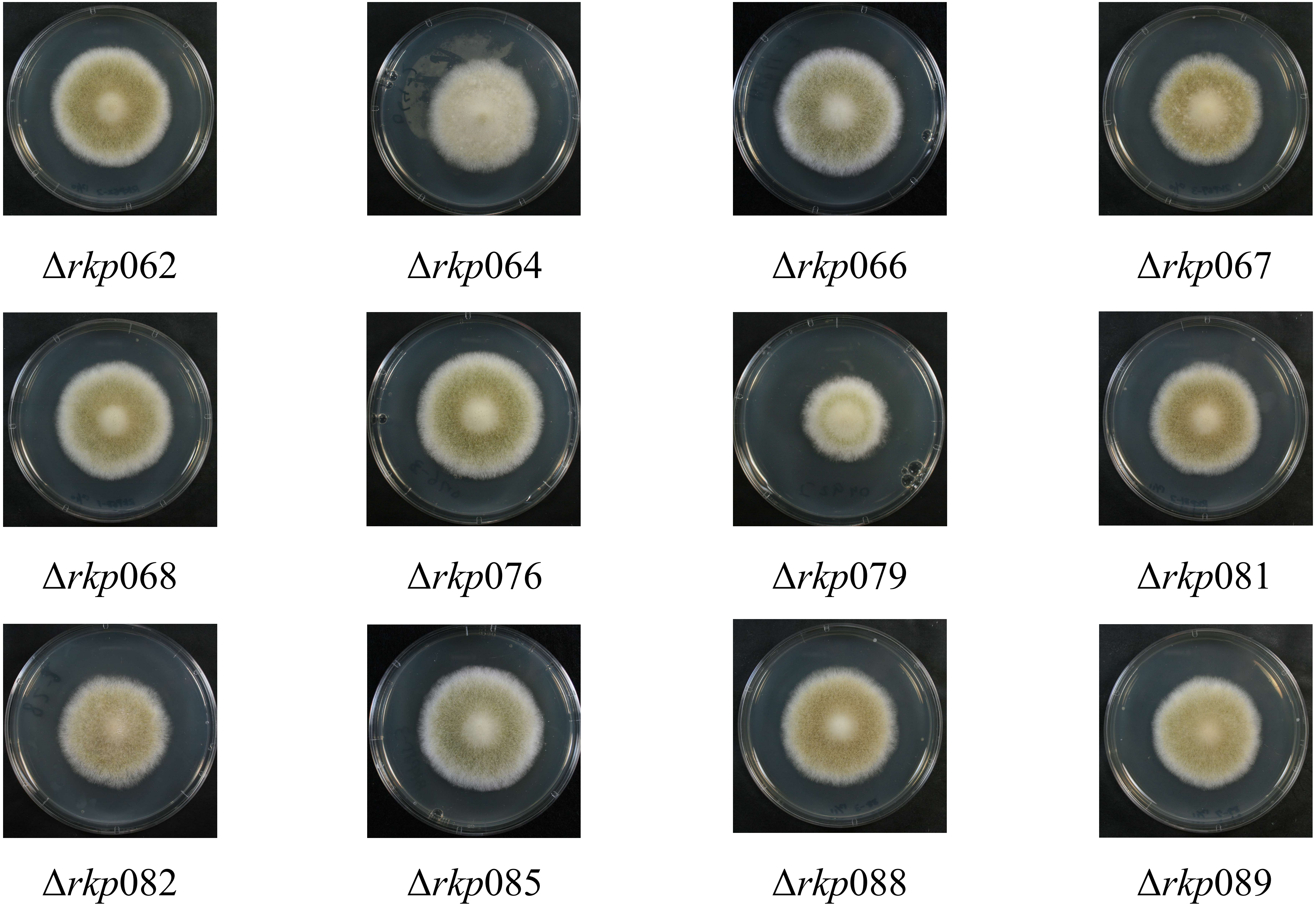
Δ*rkp*062
Δ*rkp*064
Δ*rkp*066
Δ*rkp*067
Δ*rkp*068
Δ*rkp*076
Δ*rkp*079
Δ*rkp*081
Δ*rkp*082
Δ*rkp*085
Δ*rkp*088
Δ*rkp*089

Fig. S1

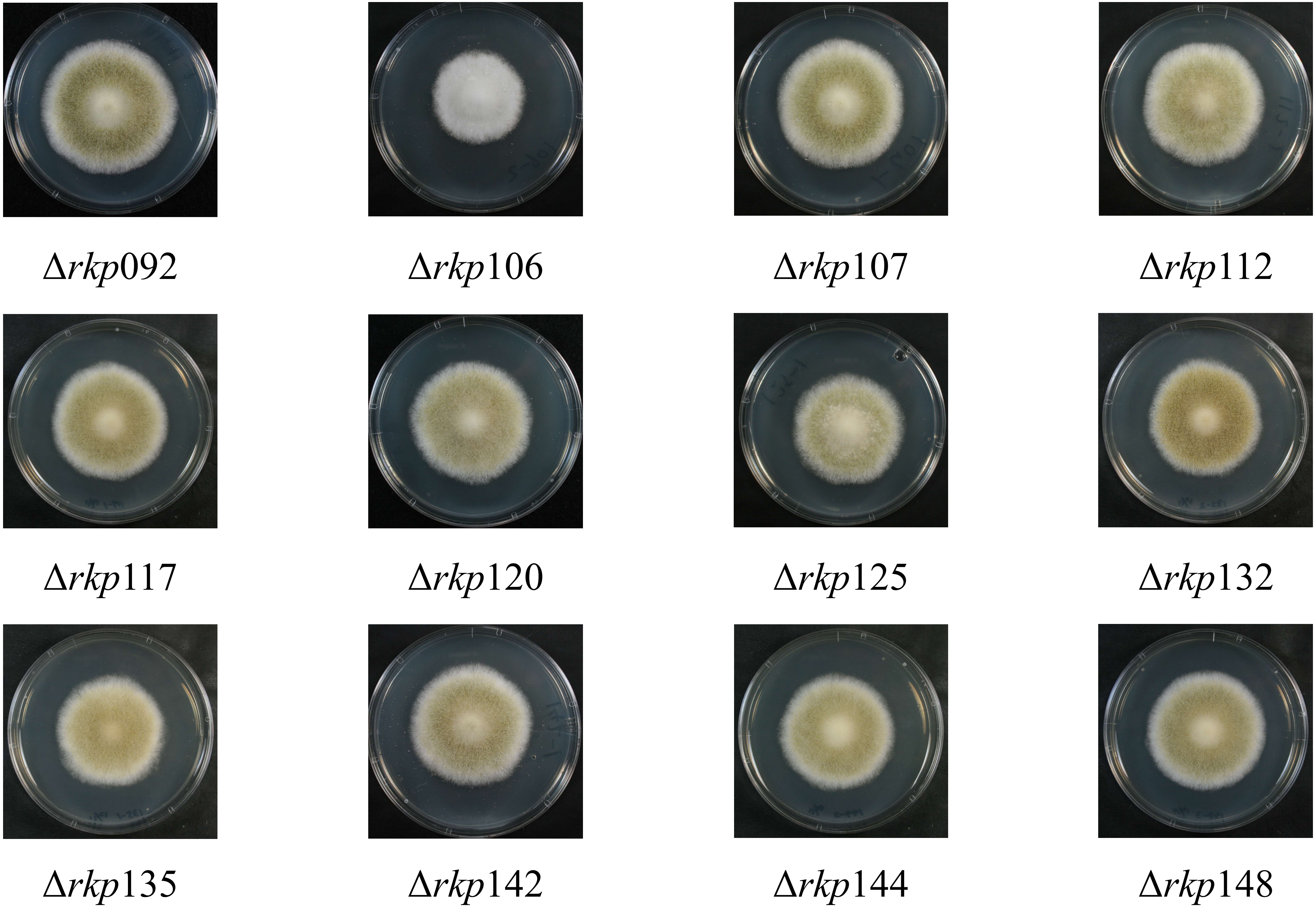
Δ*rkp*092
Δ*rkp*106
Δ*rkp*107
Δ*rkp*112
Δ*rkp*117
Δ*rkp*120
Δ*rkp*125
Δ*rkp*132
Δ*rkp*135
Δ*rkp*142
Δ*rkp*144
Δ*rkp*148

Fig. S1

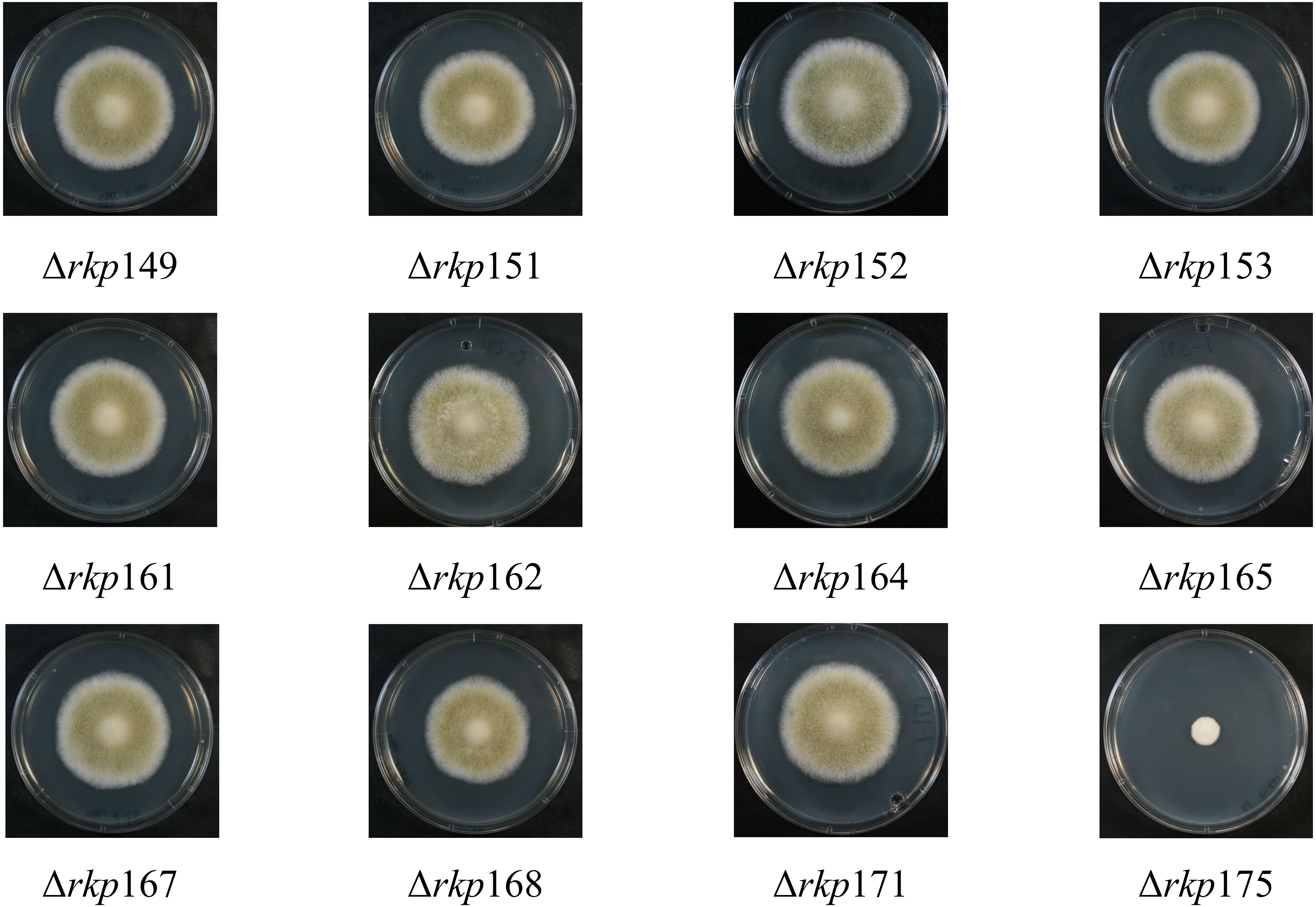

Δ*rkp*149
Δ*rkp*151
Δ*rkp*152
Δ*rkp*153
Δ*rkp*161
Δ*rkp*162
Δ*rkp*164
Δ*rkp*165
Δ*rkp*167
Δ*rkp*168
Δ*rkp*171
Δ*rkp*175

Fig. S1

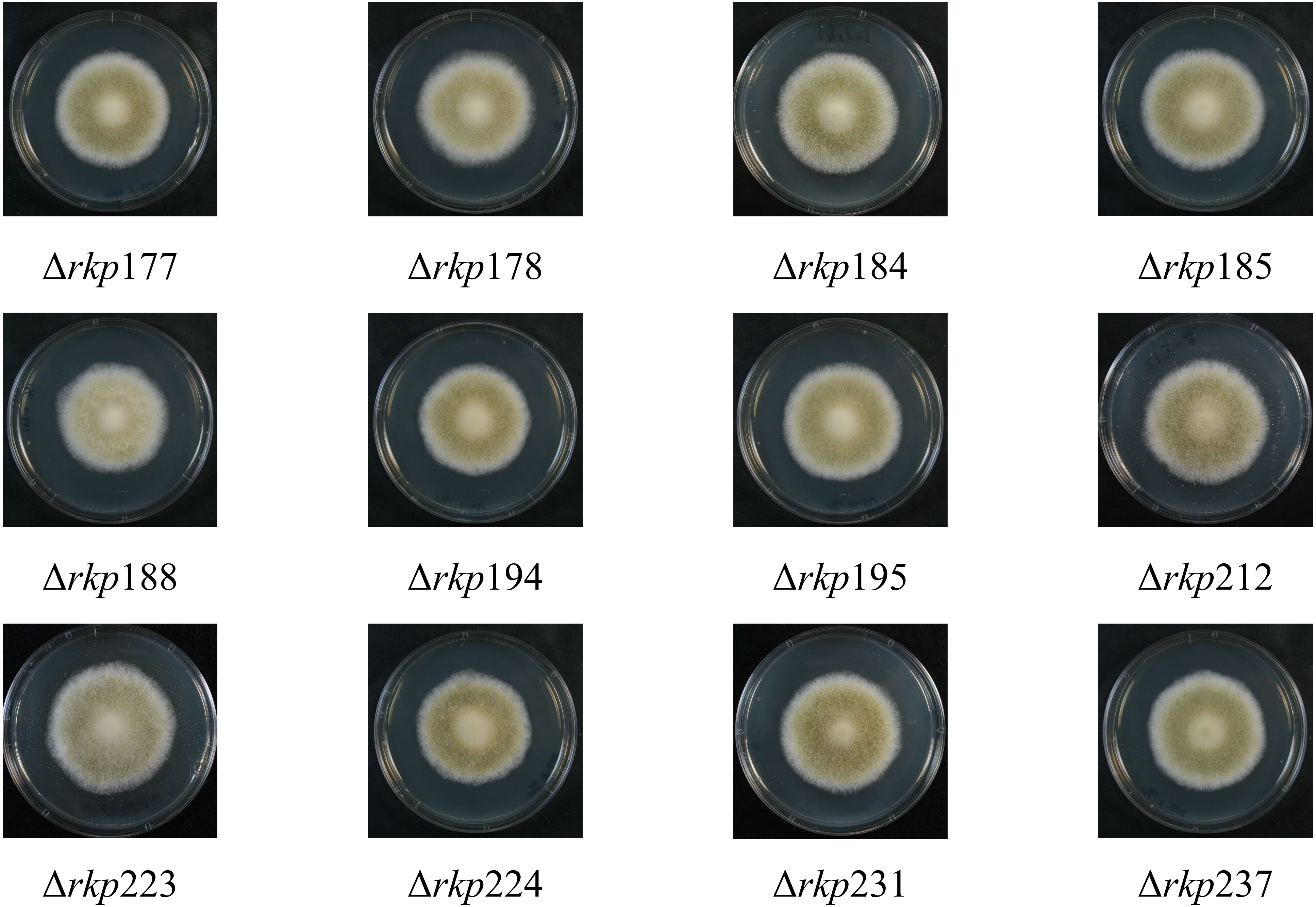

Δ*rkp*177
Δ*rkp*178
Δ*rkp*184
Δ*rkp*185
Δ*rkp*188
Δ*rkp*194
Δ*rkp*195
Δ*rkp*212
Δ*rkp*223
Δ*rkp*224
Δ*rkp*231
Δ*rkp*237

Fig. S1

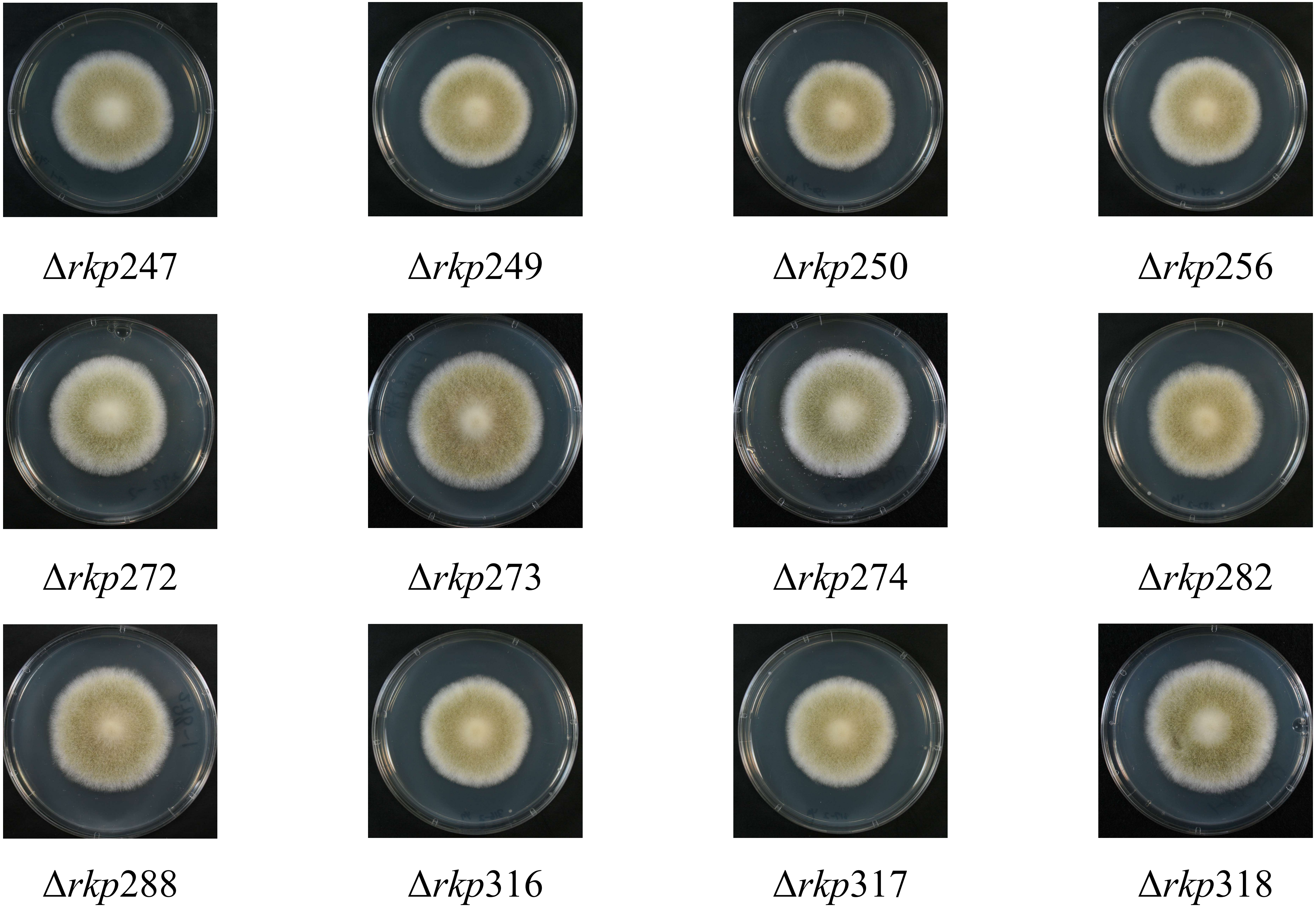
Δ*rkp*247
Δ*rkp*249
Δ*rkp*250
Δ*rkp*256
Δ*rkp*272
Δ*rkp*273
Δ*rkp*274
Δ*rkp*282
Δ*rkp*288
Δ*rkp*316
Δ*rkp*317
Δ*rkp*318

Fig. S1

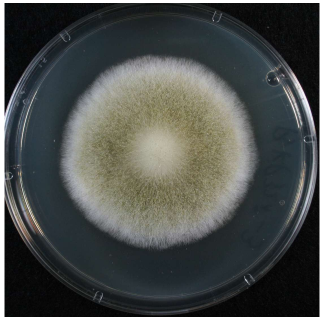

Δ*rkp*328

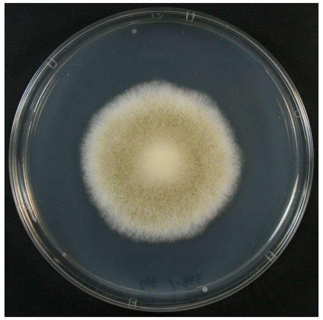

Δ*rkp*346

Fig. S1

Fig. S1. Colony morphology and conidiation of the control strain and Δ*rkp* strains. Photographs show colonies of the control strain and all Δ*rkp* strains grown on agar medium under identical culture conditions. Colonies were incubated for 5 days at 30 °C prior to imaging.
