# Supplementary material for: Effects of Aspergillus oryzae-derived rice-koji protein on the sake metabolome
Source: Appl Environ Microbiol. 2026 Feb 19;92(3):e01955-25. doi: 10.1128/aem.01955-25 (PMC12997762; doi:10.1128/aem.01955-25)
Supplement: Table S1 — List of constructed rkp gene disruptants. [file aem.01955-25-s0002.pdf]

Table S1. List of constructed *rkp* gene disruptants

RKP No.	Identity	homo/hetero	Gene ID	Discription (CAoGDX)	Predicted signal peptide (CAoGDX)
RKP002	A	homo	AO090003000321	glaB Glucoamylase	No
RKP005	C	homo	AO090120000413	Enoyl-CoA hydratase	No
RKP007	C	homo	AO090020000640	Beta-fructosidases (levanase/invertase)	No
RKP009	B	homo	AO090023000645	Puromycin-sensitive aminopeptidase and related aminopeptidases	No
RKP010	B	homo	AO090113000129	Molecular chaperones HSP105/HSP110/SSE1, HSP70 superfamily	No
RKP016	C	homo	AO090005000457	Metalloendopeptidase family - saccharolysin & thimet oligopeptidase	No
RKP017	B	homo	AO090011000300	Acid phosphatase	Yes
RKP019	A	hetero	AO090012000995	Molecular chaperones GRP78/BiP/KAR2, HSP70 superfamily	No
RKP021	B	homo	AO090012000526	Transketolase	No
RKP022	A	homo	AO090701000720	Xaa-Pro aminopeptidase	No
RKP024	B	hetero	AO090011000638	Molecular chaperones mortalin/PBP74/GRP75, HSP70 superfamily	No
RKP025	C	homo	AO090012000110	Transferrin receptor and related proteins containing the protease-associated (PA)	No
RKP027	B	homo	AO090103000007	Molecular chaperones GRP78/BiP/KAR2, HSP70 superfamily	No
RKP031	C	homo	AO090102000263	Sphingomyelinase	Yes
RKP032	C	homo	AO090001000117	Tyrosinase	No
RKP033	A	homo	AO090003000174	Phosphoenolpyruvate carboxykinase (ATP)	No
RKP040	C	homo	AO090103000073	Phospholipase C	No
RKP043	B	homo	AO090023000905	Protein ecm33	Yes
RKP045	B	hetero	AO090120000224	IMP dehydrogenase/GMP reductase	No
RKP046	C	homo	AO090011000230	Mannitol-1-phosphate/altronate dehydrogenases	No
RKP047	A	hetero	AO090011000764	Mitochondrial chaperonin, Cpn60/Hsp60p	No
RKP059	B	homo	AO090012000156	Homogentisate 1,2-dioxygenase	No
RKP060	C	homo	AO090012000855	Protoporphyrinogen oxidase	No
RKP062	B	homo	AO090005001092	Pyridine nucleotide-disulphide oxidoreductase	No
RKP064	B	hetero	AO090011000486	Dihydrolipoamide dehydrogenase	No
RKP066	C	homo	AO090003000164	Alanine aminotransferase	No
RKP067	B	hetero	AO090026000537	RAB proteins geranylgeranyltransferase component A (RAB escort protein)	No
RKP068	A	homo	AO090701000206	4-aminobutyrate aminotransferase	No
RKP076	A	homo	AO090023000923	Glutamate/leucine/phenylalanine/valine dehydrogenases	No
RKP079	B	hetero	AO090026000490	Galactose-1-phosphate uridylyltransferase	No
RKP081	B	homo	AO090003000735	Oxysterol-binding protein	No
RKP082	B	homo	AO090038000417	S-adenosylhomocysteine hydrolase	No
RKP085	B	homo	AO090003001103	Kynurenine aminotransferase, glutamine transaminase K	No
RKP088	C	homo	AO090011000576	Mannitol-1-phosphate/altronate dehydrogenases	No
RKP089	B	hetero	AO090012000998	Coproporphyrinogen III oxidase CPO/HEM13	No
RKP092	A	homo	AO090003000693	Aspartyl protease	Yes
RKP106	B	homo	AO090010000463	UDP-glucose 4-epimerase/UDP-sulfoquinovose synthase	No
RKP107	B	homo	AO090003000140	Lactoylglutathione lyase and related lyases	No
RKP112	B	hetero	AO090003000721	Homoserine dehydrogenase	No
RKP117	C	homo	AO090020000042	Predicted mutarotase	No
RKP120	B	homo	AO090023000264	Aldo/keto reductase family proteins	No
RKP125	C	homo	AO090003001045	Aldo/keto reductase family proteins	No

RKP No.	Identity	homo/hetero	Gene ID	Discription in CAoGDX	Predicted signal peptide (CAoGDX)
RKP132	C	homo	AO090023000696	Ca2+-binding protein Regucalcin/SMP30	No
RKP135	A	hetero	AO090003000090	Flavoprotein involved in thiazole biosynthesis	No
RKP142	C	homo	AO080529000039	Predicted protein	
RKP144	C	homo	AO090001000546	Purine nucleoside phosphorylase	No
RKP148	A	homo	AO090001000189	Fucose-specific lectin	No
RKP149	B	homo	AO090005000277	Predicted esterase	No
RKP151	C	homo	AO090026000752	Uncharacterized conserved protein	No
RKP152	C	homo	AO090102000599	Aspartyl aminopeptidase	No
RKP153	C	homo	AO090038000424	Predicted TIM-barrel enzyme, possibly a dioxygenase	No
RKP161	C	homo	AO090005001321	-	No
RKP162	C	hetero	AO090005000789	6-phosphogluconolactonase - like protein	No
RKP164	B	homo	AO090023000702	Alkyl hydroperoxide reductase, thiol specific antioxidant and related	No
RKP165	C	homo	AO090001000216	Iron/ascorbate family oxidoreductases	No
RKP167	C	homo	AO090009000495	Dienelactone hydrolase and related enzymes	No
RKP168	B	homo	AO090701000013	NAD-dependent malate dehydrogenase	No
RKP171	C	homo	AO090026000103	Endo-1,4-beta-xylanase A	Yes
RKP175	C	homo	AO090010000480	Actin depolymerizing factor	No
RKP177	B	homo	AO090003001047	Peptidyl-prolyl cis-trans isomerase	No
RKP178	A	homo	AO090020000521	sodC Cu2+/Zn2+ superoxide dismutase SOD1	No
RKP184	B	homo	AO090011000679	Aspartate aminotransferase/Glutamic oxaloacetic transaminase	No
RKP185	C	homo	AO090038000636	-	No
RKP188	B	hetero	AO090102000558	Nucleoside diphosphate kinase	No
RKP194	C	homo	AO090701000141	Glucosidase I	Yes
RKP195	C	homo	AO080515000161	Ngg1-interacting factor 3 protein NIF3L1	
RKP212	C	homo	AO090003000873	NADH-cytochrome b-5 reductase	No
RKP223	C	homo	AO090012000134	-	No
RKP224	C	homo	AO090026000700	Rho GDP-dissociation inhibitor	No
RKP231	C	homo	AO090023000885	Mg-dependent DNase	No
RKP237	B	homo	AO090011000475	HSP90 co-chaperone CPR7/Cyclophilin	No
RKP247	C	homo	AO090701000749	Predicted phosphatase/phosphohexomutase	No
RKP249	C	homo	AO090023000671	Uncharacterized enzymes related to aldose 1-epimerase	No
RKP250	C	homo	AO090005001418	-	No
RKP256	C	homo	AO090012000915	Dynein light chain	No
RKP272	C	homo	AO090005001627	Predicted metal-binding protein	No
RKP273	C	homo	AO090005001447	Aspartyl aminopeptidase	No
RKP274	C	homo	AO090023000256	Cytosine deaminase and related metal-dependent hydrolases	No
RKP282	C	homo	AO090012000926	Serine-pyruvate aminotransferase/archaeal aspartate aminotransferase	No
RKP288	C	homo	AO090103000142	WD40 repeat-containing protein	No
RKP316	C	homo	AO090120000297	dsRNA-specific nuclease Dicer and related ribonucleases	No
RKP317	C	homo	AO090102000246	Uracil phosphoribosyltransferase	No
RKP318	C	homo	AO090102000368	Sugar kinases, ribokinase family	No
RKP328	C	homo	AO090038000442	Aldo/keto reductase family proteins	No
RKP346	C	homo	AO090003000753	Predicted dehydrogenases and related proteins	No
