# Supplementary material for: Effects of Aspergillus oryzae-derived rice-koji protein on the sake metabolome
Source: Appl Environ Microbiol. 2026 Feb 19;92(3):e01955-25. doi: 10.1128/aem.01955-25 (PMC12997762; doi:10.1128/aem.01955-25)
Supplement: Table S2 — List of protein spots identified by proteomic analysis. [file aem.01955-25-s0003.pdf]

Table S2. List of protein spots identified by proteomic analysis

Spot No.	RKP No.	Identity Group	Disruption	NS Average	NN Average	YS Average	YN Average	NS/YS	NN/YN
2	RKP002	A	Y	0.211	0.226	0.236	0.269	0.894	0.840
3	RKP003	B	-	0.105	0.033	0.089	0.074	1.180	0.446
4	RKP003	B	-	0.07	0.066	0.087	0.043	0.805	1.535
5	RKP005	C	Y	0.234	0.333	ND	ND	-	-
6	RKP006	A	-	0.926	0.817	0.709	0.804	1.306	1.016
7	RKP007	C	Y	0.207	0.282	0.074	0.13	2.797	2.169
8	RKP002	A	Y	23.27	28.925	22.008	23.06	1.057	1.254
9	RKP009	B	Y	0.324	0.344	0.663	0.321	0.489	1.072
10	RKP010	B	Y	0.172	0.103	0.245	0.242	0.702	0.426
11	RKP011	B	N	0.164	0.181	0.185	0.246	0.886	0.736
12	RKP011	B	N	0.532	0.531	0.484	0.814	1.099	0.652
13	RKP002	A	Y	0.385	0.496	0.231	0.843	1.667	0.588
14	RKP002	A	Y	1.067	1.172	0.833	1.324	1.281	0.885
15	RKP010	B	Y	0.08	0.161	0.13	0.088	0.615	1.830
16	RKP016	C	Y	0.091	0.096	0.032	ND	2.844	-
17	RKP017	B	Y	0.75	1.057	0.341	0.531	2.199	1.991
18	RKP018	A	-	0.34	0.052	0.059	0.035	5.763	1.486
19	RKP019	A	Y	0.075	0.175	0.265	0.872	0.283	0.201
20	RKP018	A	-	0.178	0.045	0.166	0.098	1.072	0.459
21	RKP021	B	Y	0.352	0.123	0.07	0.12	5.029	1.025
22	RKP022	A	Y	0.323	0.227	0.403	0.286	0.801	0.794
23	RKP019	A	Y	0.624	0.64	0.723	0.448	0.863	1.429
24	RKP024	B	Y	0.419	0.26	0.269	0.321	1.558	0.810
25	RKP025	C	Y	0.311	0.268	0.267	0.168	1.165	1.595
26	RKP019	A	Y	0.023	0.037	0.026	0.015	0.885	2.467
27	RKP027	B	Y	0.031	0.105	0.036	0.105	0.861	1.000
28	RKP028	A	-	1.049	1.174	0.767	0.885	1.368	1.327
29	RKP029	C	N	0.128	0.168	0.05	0.048	2.560	3.500
30	RKP029	C	N	0.06	0.03	0.19	0.147	0.316	0.204
31	RKP031	C	Y	0.539	0.67	0.137	0.516	3.934	1.298
32	RKP032	C	Y	0.065	0.026	ND	ND	-	-
33	RKP033	A	Y	0.066	0.048	0.041	0.028	1.610	1.714
34	RKP034	A	-	0.086	0.095	0.059	0.091	1.458	1.044
35	RKP034	A	-	0.773	0.416	0.501	0.399	1.543	1.043
36	RKP036	C	N	0.049	0.03	0.038	0.029	1.289	1.034
37	RKP037	A	-	0.176	0.189	0.045	0.087	3.911	2.172
38	RKP037	A	-	0.681	0.454	0.183	0.191	3.721	2.377
40	RKP040	C	N	0.163	0.325	ND	0.259	-	1.255
41	RKP041	C	N	0.257	0.184	0.329	0.286	0.781	0.643
42	RKP042	A	-	0.394	0.437	0.539	0.951	0.731	0.460
43	RKP043	B	Y	0.317	0.596	0.197	0.613	1.609	0.972
44	RKP042	A	-	0.421	0.273	0.319	0.424	1.320	0.644
45	RKP045	B	Y	0.046	0.068	0.012	0.092	3.833	0.739
46	RKP046	C	Y	0.167	0.278	0.047	0.05	3.553	5.560
47	RKP047	A	Y	0.185	0.15	0.235	0.185	0.787	0.811
48	RKP045	B	Y	0.011	0.048	0.007	0.073	1.571	0.658
49	RKP042	A	-	0.172	0.131	0.245	0.127	0.702	1.031
50	RKP042	A	-	0.283	0.253	0.412	0.253	0.687	1.000
51	RKP051	A	-	0.159	0.108	0.107	0.092	1.486	1.174
52	RKP051	A	-	0.068	0.059	0.089	0.023	0.764	2.565
53	RKP053	B	-	0.196	0.118	0.235	0.082	0.834	1.439
55	RKP055	A	-	0.52	0.369	0.37	0.267	1.405	1.382
56	RKP056	A	-	0.261	0.165	0.177	0.114	1.475	1.447
57	RKP057	C	N	0.16	0.18	0.157	0.144	1.019	1.250
58	RKP058	A	-	0.164	0.149	0.051	0.043	3.216	3.465
59	RKP059	B	Y	0.156	0.076	ND	ND	-	-
60	RKP060	C	Y	0.308	0.38	0.259	0.222	1.189	1.712
61	RKP060	C	Y	0.274	0.284	0.316	0.218	0.867	1.303
62	RKP062	B	Y	0.248	0.222	0.168	0.138	1.476	1.609
63	RKP063	A	-	0.062	0.075	0.144	0.159	0.431	0.472
64	RKP064	B	Y	0.254	0.186	0.245	0.207	1.037	0.899
65	RKP018	A	-	0.084	0.071	0.046	0.026	1.826	2.731
66	RKP066	C	Y	0.261	0.12	0.209	0.166	1.249	0.723
67	RKP067	B	Y	0.357	0.13	0.31	0.15	1.152	0.867
68	RKP068	A	Y	0.243	0.132	0.079	0.081	3.076	1.630
70	RKP011	B	N	0.043	0.133	0.037	0.053	1.162	2.509
71	RKP071	A	-	15.719	20.959	13.028	17.962	1.207	1.167
72	RKP011	B	N	0.063	0.055	0.054	0.031	1.167	1.774
73	RKP073	B	N	0.228	0.218	0.303	0.2	0.752	1.090
76	RKP076	A	Y	0.149	0.133	0.139	0.124	1.072	1.073
77	RKP076	A	Y	0.618	0.679	0.799	0.61	0.773	1.113
78	RKP078	A	-	0.476	0.129	0.193	0.075	2.466	1.720
79	RKP079	B	Y	0.057	0.036	0.02	0.026	2.850	1.385
80	RKP080	A	-	0.241	0.166	0.178	0.114	1.354	1.456
81	RKP081	B	Y	0.159	0.106	0.223	0.127	0.713	0.835
82	RKP082	B	Y	0.285	0.288	0.311	0.237	0.916	1.215
83	RKP021	B	Y	0.086	0.104	0.476	0.271	0.181	0.384
84	RKP021	B	Y	0.567	0.437	0.136	0.087	4.169	5.023
85	RKP085	B	Y	0.12	0.065	0.171	0.099	0.702	0.657
86	RKP086	B	N	0.231	0.189	0.328	0.189	0.704	1.000
88	RKP088	C	Y	0.178	0.053	0.35	0.051	0.509	1.039
89	RKP089	B	Y	0.118	0.043	0.021	0.012	5.619	3.583
92	RKP092	A	Y	0.213	0.262	0.237	0.158	0.899	1.658
93	RKP093	B	-	0.08	0.077	0.167	0.098	0.479	0.786
94	RKP094	A	N	0.542	0.434	0.578	0.462	0.938	0.939
95	RKP095	B	N	0.144	0.138	0.114	0.103	1.263	1.340
96	RKP096	C	N	0.131	0.03	0.218	0.045	0.601	0.667
98	RKP098	A	-	0.06	0.04	0.036	0.021	1.667	1.905
100	RKP019	A	Y	0.1	0.224	0.119	0.21	0.840	1.067
101	RKP101	A	N	1.103	1.443	0.872	1.254	1.265	1.151
104	RKP104	A	-	0.114	0.088	0.132	0.157	0.864	0.561
105	RKP104	A	-	0.384	0.192	0.488	0.394	0.787	0.487
106	RKP106	B	Y	0.089	0.309	0.184	0.3	0.484	1.030
107	RKP107	B	Y	0.226	0.137	0.239	0.143	0.946	0.958
108	RKP106	B	Y	0.655	0.276	0.364	0.508	1.799	0.543
109	RKP109	B	-	0.533	0.42	0.47	0.371	1.134	1.132
111	RKP111	A	-	0.214	0.153	0.234	0.161	0.915	0.950
112	RKP112	B	Y	0.143	0.138	0.291	0.173	0.491	0.798
115	RKP115	C	N	0.607	0.306	0.744	0.607	0.816	0.504
116	RKP115	C	N	0.614	0.862	1.406	0.814	0.437	1.059
117	RKP117	C	Y	0.935	0.275	0.337	0.388	2.774	0.709
118	RKP118	C	N	0.22	0.25	0.349	0.369	0.630	0.678
120	RKP120	B	Y	0.615	0.465	0.68	0.482	0.904	0.965
121	RKP121	B	N	0.175	0.104	0.299	0.284	0.585	0.366
122	RKP121	B	N	0.28	0.204	0.432	0.323	0.648	0.632
123	RKP121	B	N	0.445	0.144	0.288	0.064	1.545	2.250
124	RKP124	B	-	0.076	0.053	0.145	0.03	0.524	1.767
125	RKP125	C	Y	0.757	0.83	1.068	0.768	0.709	1.081
126	RKP126	A	-	0.14	0.284	0.159	0.207	0.881	1.372
128	RKP128	B	-	0.569	0.436	0.48	0.348	1.185	1.253
129	RKP128	B	-	1.364	0.986	1.331	1.027	1.025	0.960
130	RKP130	B	N	0.911	0.703	0.921	0.668	0.989	1.052
131	RKP131	B	-	0.514	0.408	0.865	0.626	0.594	0.652
132	RKP132	C	Y	0.172	0.094	0.137	0.133	1.255	0.707
133	RKP133	A	N	0.429	0.238	0.409	0.211	1.049	1.128
134	RKP134	C	N	0.054	0.078	0.089	0.049	0.607	1.592
135	RKP135	A	Y	0.484	0.263	0.073	0.013	6.630	20.231
136	RKP131	B	-	0.084	0.082	0.2	0.129	0.420	0.636
138	RKP132	C	Y	0.046	0.072	0.031	0.051	1.484	1.412
139	RKP131	B	-	0.255	0.117	0.179	0.14	1.425	0.836
140	RKP140	C	N	0.396	0.278	0.394	0.247	1.005	1.126
142	RKP142	C	Y	0.306	0.185	0.039	0.08	7.846	2.313
143	RKP131	B	-	1.428	1.128	1.457	1.223	0.980	0.922
144	RKP144	C	Y	0.143	0.095	0.166	0.1	0.861	0.950
145	RKP145	C	N	0.328	0.23	0.057	0.063	5.754	3.651
146	RKP080	A	-	0.107	0.042	0.12	0.088	0.892	0.477
147	RKP147	C	N	ND	ND	0.381	0.257	-	-
148	RKP148	A	Y	0.073	0.044	0.134	0.176	0.545	0.250
149	RKP149	B	Y	0.527	0.314	0.578	0.423	0.912	0.742
150	RKP150	C	N	0.189	0.101	0.057	0.02	3.316	5.050
151	RKP151	C	Y	0.123	0.079	0.061	0.053	2.016	1.491
152	RKP152	C	Y	0.126	0.051	0.178	0.126	0.708	0.405
153	RKP153	C	Y	0.127	0.066	0.106	0.025	1.198	2.640
154	RKP154	A	-	0.071	0.201	0.071	0.143	1.000	1.406
156	RKP021	B	Y	0.076	0.104	0.105	0.049	0.724	2.122
157	RKP157	A	-	0.032	0.033	0.028	0.023	1.143	1.435
158	RKP158	C	N	0.035	0.035	0.039	0.016	0.897	2.188
159	RKP101	A	N	0.112	0.086	0.121	0.161	0.926	0.534
160	RKP147	C	N	0.4	0.185	0.55	0.3	0.727	0.617
161	RKP161	C	Y	0.164	0.068	ND	ND	-	-
162	RKP162	C	Y	0.107	0.067	0.026	0.016	4.115	4.188
163	RKP163	B	N	0.252	0.203	0.128	0.067	1.969	3.030
164	RKP164	B	Y	0.399	0.267	0.533	0.501	0.749	0.533
165	RKP165	C	Y	0.244	0.14	0.308	0.153	0.792	0.915
166	RKP034	A	-	0.088	0.055	0.128	0.067	0.688	0.821
167	RKP167	C	Y	0.266	0.109	0.288	0.188	0.924	0.580
168	RKP168	B	Y	ND	0.114	ND	0.053	-	2.151
170	RKP164	B	Y	0.109	0.077	0.165	0.148	0.661	0.520
171	RKP171	C	Y	0.044	0.107	0.051	0.095	0.863	1.126
175	RKP175	C	Y	0.254	0.217	0.35	0.347	0.726	0.625
176	RKP176	B	N	0.507	0.242	0.069	0.074	7.348	3.270
177	RKP177	B	Y	0.454	0.202	0.741	0.49	0.613	0.412
178	RKP178	A	Y	1.611	1.479	1.639	1.355	0.983	1.092
181	RKP181	C	N	0.184	0.186	0.223	0.257	0.825	0.724
183	RKP183	B	-	0.066	0.045	0.051	0.044	1.294	1.023
184	RKP184	B	Y	0.13	0.037	0.094	0.075	1.383	0.493
185	RKP185	C	Y	0.019	0.054	0.128	0.068	0.148	0.794
186	RKP186	A	-	0.025	0.033	0.037	0.042	0.676	0.786
187	RKP176	B	N	0.237	0.129	0.11	0.109	2.155	1.183
188	RKP188	B	Y	0.023	0.058	0.073	0.061	0.315	0.951
192	RKP101	A	N	0.038	0.116	0.091	0.22	0.418	0.527
194	RKP194	C	Y	0.071	0.058	0.101	0.05	0.703	1.160
195	RKP195	C	Y	0.205	0.055	0.287	0.151	0.714	0.364
198	RKP011	B	N	0.052	0.034	0.067	0.03	0.776	1.133
199	RKP199	C	N	0.135	0.129	0.024	0.04	5.625	3.225
203	RKP203	A	-	0.23	0.139	0.164	0.158	1.402	0.880
204	RKP204	B	-	0.096	0.057	0.302	0.315	0.318	0.181
208	RKP208	C	N	0.193	0.054	0.37	0.295	0.522	0.183
210	RKP021	B	Y	ND	ND	0.064	0.034	-	-
212	RKP212	C	Y	0.02	0.031	0.022	0.04	0.909	0.775
214	RKP214	B	N	ND	ND	0.317	0.265	-	-
215	RKP176	B	N	ND	ND	1.197	1.009	-	-
220	RKP220	C	N	ND	ND	0.311	0.338	-	-
222	RKP222	C	N	ND	ND	0.044	0.098	-	-
223	RKP223	C	Y	ND	ND	0.083	0.068	-	-
224	RKP224	C	Y	0.086	0.077	0.157	0.079	0.548	0.975
226	RKP161	C	Y	0.025	0.03	0.083	0.049	0.301	0.612
227	RKP148	A	Y	ND	ND	0.476	0.368	-	-
228	RKP148	A	Y	ND	ND	0.398	0.658	-	-
229	RKP126	A	-	ND	ND	0.744	0.987	-	-
230	RKP222	C	N	0.166	0.068	0.488	0.274	0.340	0.248
231	RKP231	C	Y	0.079	0.045	0.215	0.165	0.367	0.273
236	RKP021	B	Y	ND	ND	0.168	0.257	-	-
237	RKP237	B	Y	0.137	0.037	0.166	0.263	0.825	0.141
238	RKP238	C	N	0.058	0.027	0.053	0.054	1.094	0.500
239	RKP029	C	N	ND	ND	0.098	0.064	-	-
240	RKP222	C	N	0.068	0.04	0.14	0.177	0.486	0.226
242	RKP042	A	-	0.055	0.05	0.158	0.119	0.348	0.420
243	RKP104	A	-	0.051	0.023	0.036	0.116	1.417	0.198
246	RKP034	A	-	0.071	0.033	0.115	0.079	0.617	0.418
247	RKP247	C	Y	0.057	ND	0.156	0.083	0.365	-
248	RKP162	C	Y	0.113	ND	0.149	0.154	0.758	-
249	RKP249	C	Y	0.623	0.268	0.404	0.224	1.542	1.196
250	RKP250	C	Y	0.032	0.035	0.049	0.09	0.653	0.389
252	RKP042	A	-	0.127	0.12	0.131	0.22	0.969	0.545
253	RKP237	B	Y	0.069	0.044	0.115	0.11	0.600	0.400
256	RKP256	C	Y	0.066	0.048	0.135	0.128	0.489	0.375
257	RKP256	C	Y	0.045	0.017	0.074	0.086	0.608	0.198
258	RKP237	B	Y	0.085	0.014	0.128	0.091	0.664	0.154
259	RKP247	C	Y	ND	ND	ND	0.055	-	-
260	RKP260	A	-	0.071	0.047	0.043	0.043	1.651	1.093
272	RKP272	C	Y	0.05	0.011	0.074	0.047	0.676	0.234
273	RKP273	C	Y	0.211	0.068	0.104	0.071	2.029	0.958
274	RKP274	C	Y	0.066	0.065	0.026	0.042	2.538	1.548
282	RKP282	C	Y	0.185	0.061	0.215	0.112	0.860	0.545
284	RKP011	B	N	0.117	0.033	0.153	0.026	0.765	1.269
286	RKP222	C	N	0.092	0.04	0.339	0.188	0.271	0.213
288	RKP288	C	Y	ND	ND	0.264	0.174	-	-
291	RKP247	C	Y	0.011	ND	0.096	0.027	0.115	-
293	RKP135	A	Y	ND	ND	0.482	0.277	-	-
294	RKP135	A	Y	0.061	0.05	0.503	0.169	0.121	0.296
297	RKP249	C	Y	ND	ND	0.385	0.337	-	-
300	RKP040	C	Y	0.022	0.047	0.292	0.243	0.075	0.193
302	RKP302	C	N	0.075	0.031	0.109	0.066	0.688	0.470
308	RKP308	C	N	0.327	0.393	0.708	1.012	0.462	0.388
316	RKP316	C	Y	0.066	0.031	0.068	0.026	0.971	1.192
317	RKP317	C	Y	0.149	0.087	0.166	0.083	0.898	1.048
318	RKP318	C	Y	0.059	0.022	0.138	0.034	0.428	0.647
320	RKP320	C	N	0.03	0.044	0.026	0.049	1.154	0.898
321	RKP208	C	N	0.053	0.031	0.135	0.044	0.393	0.705
325	RKP115	C	N	0.361	0.157	0.283	0.211	1.276	0.744
326	RKP326	C	N	0.068	0.035	0.061	0.02	1.115	1.750
327	RKP327	B	N	0.104	ND	0.06	0.028	1.733	-
328	RKP328	C	Y	0.04	0.03	ND	ND	-	-
330	RKP247	C	Y	0.165	0.054	ND	ND	-	-
331	RKP034	A	-	0.154	0.067	0.071	0.068	2.169	0.985
332	RKP222	C	N	0.039	0.019	0.056	0.039	0.696	0.487
333	RKP333	B	N	0.07	ND	0.007	ND	10.000	-
334	RKP334	C	N	0.049	0.021	0.097	0.041	0.505	0.512
336	RKP214	B	N	0.142	0.04	ND	ND	-	-
343	RKP055	A	-	0.133	0.112	0.089	0.08	1.494	1.400
344	RKP344	B	N	0.056	0.039	0.052	0.023	1.077	1.696
346	RKP346	C	Y	0.074	0.038	0.074	0.042	1.000	0.905
348	RKP348	B	-	0.119	0.06	0.15	0.043	0.793	1.395
349	RKP088	C	Y	0.081	0.039	0.183	0.033	0.443	1.182
351	RKP351	A	-	ND	ND	0.306	0.112	-	-
352	RKP352	B	N	0.085	0.037	0.077	0.035	1.104	1.057
359	RKP101	A	N	0.093	0.132	0.167	0.181	0.557	0.729

Y : yes, N : no, - : not performed.
NS : Nihonbare–RIB128–Shubosoe
NN : Nihonbare–RIB128–Nakatome
YS : Yamadanishiki–RIBOIS01–Shubosoe
YN : Yamadanishiki–RIBOIS01–Nakatome
