# Supplementary material for: Effects of Aspergillus oryzae-derived rice-koji protein on the sake metabolome
Source: Appl Environ Microbiol. 2026 Feb 19;92(3):e01955-25. doi: 10.1128/aem.01955-25 (PMC12997762; doi:10.1128/aem.01955-25)
Supplement: Table S3 — Summary of analytical results for rice-koji using Δrkp strains. [file aem.01955-25-s0004.pdf]

Table S3. Summary of analytical results for rice-*koji* using Δ*rkp* strains

	Growth phenotype					rice-*koji* analysis							
	Mycelial content in rice-*koji*	Dry cell weight in liquid culture	Colony diameter	Conidial production on agar medium	Total Protain	α-Amylase per rice-*koji*	α-Amylase per mycelial content	Glucoamylase per rice-*koji*	Glucoamylase per mycelial content	Acid carboxypeptidase per rice-*koji*	Acid carboxypeptidase per mycelial content	Acid protease per rice-*koji*	Acid protease per mycelial content
Control	1.0000	1.0000	1.0000	1.0000	1.0000	1.0000	1.0000	1.0000	1.0000	1.0000	1.0000	1.0000	1.0000
Δ*rkp* 002	0.9971	0.5656	0.9827	1.0121	0.9281	1.1539	1.1574	0.0825	0.0827	0.9039	0.9066	1.4186	1.4228
Δ*rkp* 005	0.6805	0.1151	0.9386	1.5745	0.4702	0.8755	1.2865	0.5097	0.7490	0.6274	0.9220	0.6400	0.9405
Δ*rkp* 007	1.1762	0.9065	1.0093	1.6624	1.0590	0.8489	0.7217	0.9683	0.8233	0.9345	0.7945	1.1970	1.0177
Δ*rkp* 009	1.0745	1.0514	0.9929	1.1788	1.1024	1.3122	1.2212	1.0512	0.9783	1.0896	1.0140	1.0985	1.0223
Δ*rkp* 016	1.1229	0.7014	1.0155	1.5803	1.2325	1.0052	0.8952	1.0825	0.9640	0.9861	0.8782	1.1222	0.9993
Δ*rkp* 017	0.9059	1.1102	1.0030	1.3805	0.9218	0.7991	0.8821	0.8864	0.9785	0.8944	0.9874	1.0736	1.1851
Δ*rkp* 019	0.3651	0.3845	0.9614	1.1899	0.6096	0.4196	1.1493	0.5747	1.5743	0.6425	1.7600	0.7689	2.1062
Δ*rkp* 021	0.9880	1.7104	1.0300	0.8856	1.0665	1.0879	1.1012	0.9837	0.9957	1.1565	1.1705	0.9585	0.9702
Δ*rkp* 022	0.9250	0.3353	0.9483	1.1345	0.8749	0.8220	0.8887	0.8533	0.9225	0.8511	0.9201	0.9995	1.0806
Δ*rkp* 024	0.4258	0.0391	0.9356	1.4104	0.3903	0.3573	0.8391	0.2683	0.6301	0.3755	0.8819	0.5108	1.1996
Δ*rkp* 025	1.0534	1.1836	1.0271	1.4389	1.1614	0.9246	0.8777	1.0843	1.0293	1.0662	1.0122	1.1360	1.0784
Δ*rkp* 027	1.2987	0.8881	0.9739	1.4904	1.0476	0.8513	0.6555	0.9864	0.7596	0.9426	0.7259	0.9725	0.7489
Δ*rkp* 031	1.0586	1.5947	1.0333	1.1994	0.9166	0.8437	0.7970	0.8724	0.8241	0.8358	0.7896	1.0075	0.9517
Δ*rkp* 032	1.0008	1.5697	1.0457	1.2139	0.8593	0.7521	0.7515	0.7929	0.7922	0.8358	0.8351	1.0135	1.0126
Δ*rkp* 033	0.8435	1.5437	1.0583	1.1251	0.9583	0.8345	0.9893	0.8834	1.0472	0.8430	0.9993	1.0896	1.2917
Δ*rkp* 040	0.9895	0.4333	0.9731	1.2551	0.9508	0.9295	0.9394	0.9478	0.9578	0.8863	0.8956	0.9201	0.9298
Δ*rkp* 043	1.0225	0.9060	0.9448	0.2887	0.7329	0.8061	0.7884	0.7968	0.7793	0.7916	0.7742	1.0143	0.9920
Δ*rkp* 045	0.6927	0.3344	1.0104	0.7183	0.4288	0.5973	0.8623	0.5416	0.7819	0.6676	0.9638	0.7555	1.0907
Δ*rkp* 046	1.0170	0.3636	0.9500	0.0746	0.9664	0.9383	0.9226	0.8766	0.8619	0.9286	0.9130	0.8904	0.8755
Δ*rkp* 047	0.7988	1.0177	0.9823	0.7118	0.7383	0.8406	1.0524	0.8381	1.0492	0.7294	0.9131	0.9112	1.1407
Δ*rkp* 059	1.0157	0.8034	1.0133	0.9587	1.0542	1.1474	1.1296	1.0575	1.0412	0.9341	0.9197	0.9666	0.9516
Δ*rkp* 060	0.1257	0.2519	0.7615	0.0203	0.0000	0.1337	1.0641	0.0736	0.5856	0.0800	0.6365	0.0126	0.1005
Δ*rkp* 062	0.9164	1.5714	0.9916	1.0244	0.8810	0.8745	0.9543	0.8574	0.9357	0.9745	1.0635	1.0227	1.1160
Δ*rkp* 064	0.7408	0.0931	0.9659	0.4918	0.7033	0.5801	0.7830	0.8077	1.0903	0.6279	0.8476	0.6156	0.8309
Δ*rkp* 066	1.0886	0.4291	1.0435	0.8997	1.0647	1.1299	1.0380	1.0418	0.9570	1.1019	1.0123	0.9361	0.8599
Δ*rkp* 067	0.9519	1.7863	0.9736	0.9804	0.7598	0.8437	0.8863	0.8863	0.9311	0.7865	0.8262	0.9386	0.9860
Δ*rkp* 068	1.0098	1.2824	1.0367	1.3315	1.0075	1.0352	1.0251	1.0568	1.0465	0.9287	0.9197	0.9439	0.9347
Δ*rkp* 076	1.0162	1.2590	1.0405	0.9526	1.0272	1.0297	1.0133	0.9944	0.9786	0.9876	0.9719	0.9666	0.9511
Δ*rkp* 079	0.9372	0.4538	0.8935	0.3374	1.0752	0.8189	0.8737	0.8940	0.9540	0.8703	0.9286	0.8688	0.9270
Δ*rkp* 081	0.9638	0.9145	0.9844	2.0834	1.0182	0.9692	1.0056	1.0723	1.1126	0.9102	0.9444	0.9220	0.9567
Δ*rkp* 082	0.6401	0.1096	0.9481	0.7718	0.5815	0.4945	0.7724	0.5118	0.7994	0.5994	0.9363	0.6658	1.0401
Δ*rkp* 085	1.0516	1.0335	1.0611	1.0551	1.0560	0.9854	0.9371	1.0345	0.9838	0.9732	0.9255	0.9858	0.9374
Δ*rkp* 088	0.9462	1.2854	1.0474	2.1974	0.9106	0.9335	0.9866	0.9017	0.9530	0.9418	0.9954	0.7208	0.7618
Δ*rkp* 089	0.5126	0.1806	0.9592	1.2386	0.7114	0.5750	1.1218	0.4718	0.9204	0.5558	1.0844	0.5108	0.9965
Δ*rkp* 092	1.0402	1.6128	1.0930	0.9419	1.0419	0.9598	0.9227	1.0218	0.9822	0.9846	0.9465	0.9682	0.9307
Δ*rkp* 106	0.2667	0.3355	0.7976	0.0622	0.2325	0.2341	0.8778	0.1418	0.5318	0.1843	0.6909	0.4354	1.6325
Δ*rkp* 107	0.9752	0.8446	1.0433	0.8733	1.0588	1.0064	1.0321	1.0037	1.0293	0.8796	0.9020	0.9395	0.9635
Δ*rkp* 112	0.5765	0.0225	0.9842	0.8766	0.5644	0.4787	0.8304	0.4642	0.8051	0.5589	0.9695	0.6558	1.1375
Δ*rkp* 117	1.0855	1.3119	1.0343	1.6246	0.8487	0.9432	0.8690	0.9812	0.9040	1.0733	0.9888	0.8651	0.7970
Δ*rkp* 120	0.9747	0.7544	1.0056	1.1060	1.0348	1.1182	1.1472	1.0306	1.0574	0.9954	1.0212	0.9690	0.9941
Δ*rkp* 125	0.9459	0.5407	0.9619	0.2368	0.9578	1.0821	1.1441	0.9215	0.9743	0.9022	0.9539	0.9008	0.9523
Δ*rkp* 132	1.0373	1.0605	1.0154	2.1391	0.8702	0.8852	0.8534	0.9322	0.8987	0.8637	0.8326	0.8695	0.8383
Δ*rkp* 135	0.9122	0.1132	0.9939	1.5986	0.8648	0.8755	0.9597	0.9312	1.0208	0.8403	0.9211	0.7908	0.8669
Δ*rkp* 142	1.0716	1.4119	1.0241	1.0694	1.0896	1.1287	1.0533	1.0376	0.9683	1.1513	1.0744	1.1079	1.0338
Δ*rkp* 144	0.9831	0.8933	1.0373	1.4598	0.9375	1.0556	1.0737	1.0088	1.0261	1.0714	1.0898	0.8709	0.8859
Δ*rkp* 148	1.0713	1.0233	1.0905	1.1556	0.9940	1.0971	1.0242	1.0115	0.9443	1.1402	1.0643	0.9528	0.8894
Δ*rkp* 149	0.9114	0.8898	1.0597	1.0784	0.9334	1.1749	1.2891	0.8999	0.9874	1.0457	1.1474	0.8970	0.9842
Δ*rkp* 151	0.9099	1.3463	1.0620	1.0115	0.8836	1.0437	1.1470	0.9472	0.9697	1.0454	1.1489	0.8858	0.9735
Δ*rkp* 152	1.0182	1.1492	1.0839	1.1183	1.0280	1.1136	1.0937	1.0120	0.9939	0.8981	0.8821	0.9457	0.9289
Δ*rkp* 153	0.9768	1.4390	1.0620	0.8863	0.9509	1.1005	1.1267	0.9558	0.9623	1.0928	1.1188	0.9776	1.0009
Δ*rkp* 161	0.9933	1.0480	1.0735	0.9655	0.9213	1.0083	1.0151	0.9958	0.9703	1.0426	1.0496	0.9627	0.9692
Δ*rkp* 162	0.5551	0.2442	0.9681	0.5739	0.6311	0.5114	0.9212	0.4383	0.7896	0.5023	0.9049	0.6403	1.1535
Δ*rkp* 164	1.0749	1.0772	0.9973	1.3238	1.0314	1.0041	0.9341	0.9502	0.8840	1.0376	0.9653	0.9132	0.8495
Δ*rkp* 165	0.9307	0.3776	0.9767	1.0583	0.9065	0.9400	1.0101	0.9177	0.9861	0.8121	0.8727	0.8605	0.9246
Δ*rkp* 167	1.0262	0.8365	1.0508	1.0037	0.8783	1.0660	1.0387	0.9549	1.9949	1.0807	1.0531	0.9082	0.8849
Δ*rkp* 168	0.8534	0.8671	1.0597	0.9414	0.8527	1.1423	1.3386	0.8899	1.0428	1.2285	1.4396	0.8276	0.9698
Δ*rkp* 171	0.9932	0.4429	1.0294	0.9253	0.8911	0.9773	0.9840	0.8299	0.8355	0.8899	0.8960	0.8822	0.8882
Δ*rkp* 175	0.4787	0.0678	0.2645	0.0110	0.0000	0.1231	0.2571	0.0177	0.0175	0.0898	0.1875	0.0000	0.0000
Δ*rkp* 177	1.0076	0.6673	1.0606	1.0889	0.9186	1.0364	1.0285	0.9501	1.3184	1.1390	1.1304	0.8541	0.8477
Δ*rkp* 178	0.7207	0.3085	1.0835	0.6610	0.5499	0.8486	1.1775	0.6486	0.7303	0.9317	1.2928	0.6782	0.9411
Δ*rkp* 184	1.0665	1.2929	1.0209	0.8717	1.0092	1.2743	1.1948	1.0387	0.9740	1.3138	1.2319	1.0326	0.9682
Δ*rkp* 185	0.8881	0.7306	1.0813	0.8990	0.7948	0.9655	1.0872	0.8593	1.3836	1.0501	1.1824	0.8059	0.9075
Δ*rkp* 188	0.6210	0.6282	1.0034	0.6121	0.4261	0.6513	1.0488	0.5561	0.6111	0.7018	1.1300	0.4759	0.7662
Δ*rkp* 194	0.9108	1.1614	1.0063	0.9810	0.7759	0.8229	0.9036	0.8179	0.8980	0.7554	0.8295	0.6734	0.7394
Δ*rkp* 195	1.1084	1.0071	1.0564	0.9057	0.9348	0.9588	0.8651	0.8913	0.8042	0.8994	0.8115	0.8011	0.7228
Δ*rkp* 212	1.0354	1.0228	1.0307	0.9715	1.0331	1.1788	1.1385	1.0783	1.0414	0.9292	0.8974	1.0342	0.9988
Δ*rkp* 223	0.9693	0.9861	1.0427	1.0192	0.9151	1.0984	1.1332	0.9888	1.0200	1.0007	1.0324	1.0358	1.0685
Δ*rkp* 224	1.0059	1.2634	1.0214	1.1334	0.9036	0.8799	0.8748	0.9058	0.9006	0.8750	0.8699	0.7842	0.7797
Δ*rkp* 231	0.9758	1.0061	1.0189	1.2715	0.9886	1.2429	1.2737	1.0099	1.0349	0.9536	0.9773	1.0546	1.0808
Δ*rkp* 237	1.0256	1.0024	1.0884	1.0268	0.9348	0.9646	0.9406	0.9018	0.8793	0.8694	0.8477	0.7529	0.7341
Δ*rkp* 247	1.0007	1.0203	1.0906	1.0798	0.8671	0.8645	0.8639	1.0108	1.0102	0.8553	0.8548	1.1036	1.1028
Δ*rkp* 249	1.0305	1.1091	1.0206	0.9963	0.9374	0.9625	0.9340	0.9194	0.8922	0.8600	0.8346	1.1692	1.1346
Δ*rkp* 250	0.9869	1.1769	1.0157	0.9523	0.9947	0.9591	0.9718	0.9883	1.0014	0.9170	0.9292	1.1233	1.1382
Δ*rkp* 256	0.9660	0.7766	1.0116	0.8132	0.9504	0.9536	0.9872	0.9573	0.9910	0.7806	0.8081	1.1714	1.2127
Δ*rkp* 272	0.9959	1.1879	1.0103	0.7093	0.9852	1.1835	1.1884	0.9638	0.9678	1.0335	1.0378	1.0263	1.0306
Δ*rkp* 273	0.8507	1.1191	1.1024	1.2437	0.8090	0.9319	1.0954	0.7755	0.9116	0.8159	0.9591	0.8916	1.0480
Δ*rkp* 274	1.1171	1.0106	1.0582	1.1931	1.0348	1.1439	1.0239	1.0014	0.8964	1.0633	0.9518	1.0890	0.9748
Δ*rkp* 282	0.8668	1.1279	1.0570	1.0413	0.8619	0.9439	1.0889	0.9476	1.0932	0.8796	1.0147	1.1276	1.3009
Δ*rkp* 288	0.9063	0.3841	1.0446	1.0814	0.9784	1.0518	1.1606	0.9526	1.0511	0.9166	1.0114	1.0169	1.1221
Δ*rkp* 316	0.9604	0.9399	1.0047	0.9462	0.9348	0.7884	0.8209	0.6682	0.6958	0.8449	0.8798	1.1517	1.1992
Δ*rkp* 317	1.0118	0.8840	1.0020	0.8277	0.8749	0.8831	0.8728	0.9424	0.9314	0.8744	0.8642	1.0489	1.0366
Δ*rkp* 318	0.9923	1.3024	1.1049	1.0652	1.0656	1.1823	1.1915	1.0279	1.0358	0.9775	0.9850	1.1204	1.1291
Δ*rkp* 328	1.0443	1.2808	1.0455	1.3665	1.1614	1.2825	1.2281	1.0949	1.0484	1.0001	0.9577	1.1126	1.0654
Δ*rkp* 346	0.9048	0.7287	0.9911	0.8583	0.8176	0.8968	0.9912	0.8935	0.9875	0.8585	0.9488	1.1211	1.2390

Values shown represent mean relative values calculated from independently obtained disruptant strains and are expressed relative to the control strain. Measures of variance are not shown.
