# Supplementary material for: Effects of Aspergillus oryzae-derived rice-koji protein on the sake metabolome
Source: Appl Environ Microbiol. 2026 Feb 19;92(3):e01955-25. doi: 10.1128/aem.01955-25 (PMC12997762; doi:10.1128/aem.01955-25)
Supplement: Table S4 — Summary of analytical results for sake produced using Δrkp strains. [file aem.01955-25-s0005.pdf]

Table S4. Summary of analytical results for sake produced using Δ*rkp* strains

	sake analysis						Volatile compounds in sake				Organic acids in sake									Amino acids in sake
	CO2	Cake weight	EtOH	Nihonshu-do	Acidity	Amino acid	EtOCap	iAmOAc	iAmOH	EtOAc	phosphoric	citric	succinic	malic	lactic	fumaric	acetic	pyroglutamic	Total acid	Asp
Control	1.0000	1.0000	1.0000	1.0000	1.0000	1.0000	1.0000	1.0000	1.0000	1.0000	1.0000	1.0000	1.0000	1.0000	1.0000	1.0000	1.0000	1.0000	1.0000	1.0000
Δ*rkp* 002	0.6464	1.3537	0.7164	10.1372	0.9677	1.2369	0.8439	1.1024	1.3654	1.1006	0.5657	1.1431	0.8183	0.5756	0.6216	0.2478	1.4641	1.2399	0.8695	1.7744
Δ*rkp* 005	0.9775	1.3186	0.9966	0.5583	0.9754	0.9414	0.9001	0.8503	1.0460	0.9269	0.8177	0.8477	1.0946	0.7386	0.9388	0.9519	1.1199	0.8253	0.9616	1.1555
Δ*rkp* 007	1.0127	0.8861	1.0667	0.9402	0.9985	0.9488	0.9513	0.9987	1.0145	1.0213	1.0002	0.7851	0.9378	1.1112	1.0002	0.9045	0.9429	0.9305	0.9763	0.8652
Δ*rkp* 009	0.9784	1.0399	0.9847	1.2264	1.0617	0.8139	0.8238	1.0351	1.0006	0.9477	1.0024	0.8558	0.9531	1.0640	0.9392	0.7358	0.9423	1.1623	0.9990	0.9740
Δ*rkp* 016	1.0155	0.8871	1.0715	0.9401	0.9849	0.9516	1.0229	0.9948	1.0185	0.9866	1.0204	0.8203	0.9445	1.1096	1.0210	0.8275	1.0066	0.9527	0.9965	0.8632
Δ*rkp* 017	0.9850	1.0318	1.0419	1.4034	1.0680	1.1031	0.8892	0.8224	0.9948	0.9724	1.0332	1.0002	1.0600	0.8687	0.9487	1.2679	1.1993	1.0413	1.0258	1.4952
Δ*rkp* 019	0.9407	1.3682	0.9902	1.1469	1.0009	0.8999	0.9400	0.8684	1.0594	1.0030	0.8293	0.8977	1.0766	0.7774	0.9130	0.9864	1.1356	1.1193	0.9686	1.1004
Δ*rkp* 021	0.9944	1.0221	1.0202	1.0313	0.9961	0.8879	0.7541	0.9153	0.9969	0.8783	0.9560	0.9700	0.9435	1.0340	0.9225	0.6476	0.9134	1.1422	0.9852	0.8608
Δ*rkp* 022	1.0014	1.0170	1.0551	1.0974	1.0814	1.0567	1.0112	0.8207	0.9765	0.9231	1.0436	0.9944	1.0959	0.9262	0.9727	1.2592	1.2655	1.0621	1.0605	1.2615
Δ*rkp* 024	0.7706	1.8836	0.8219	3.5804	0.9541	0.9877	0.7819	0.7867	1.1724	0.9238	0.5914	0.9835	1.0918	0.5334	0.7035	1.0487	1.2583	0.9359	0.8863	1.3340
Δ*rkp* 025	1.0174	0.9833	1.0688	1.3868	1.0094	1.0135	0.9641	0.9779	0.9874	0.9816	1.0270	0.8548	0.9619	1.0879	1.0234	1.0227	1.0710	1.0765	1.0129	1.0370
Δ*rkp* 027	0.9951	1.0350	1.0045	1.3125	1.0106	0.9768	0.9391	0.9446	1.0019	0.9906	1.0258	0.8738	0.9563	1.0453	0.9985	1.0910	1.0837	1.1631	1.0027	1.0569
Δ*rkp* 031	0.9873	1.1268	1.0620	1.4695	1.0634	1.0636	0.9276	0.8805	1.0028	0.9963	1.0195	0.9956	1.0677	0.9757	0.9621	1.1752	1.1168	1.1675	1.0318	1.2406
Δ*rkp* 032	0.9862	1.1539	1.0208	1.3374	1.0866	1.0843	0.9595	0.8586	0.9806	0.9604	1.0671	1.0303	1.1011	0.9623	0.9680	1.3337	1.2754	1.1673	1.0750	1.3365
Δ*rkp* 033	0.9897	1.1632	1.0367	1.2630	1.0612	1.1012	0.8930	0.8039	1.0274	0.9295	1.0083	0.9850	1.0612	0.8195	0.9319	1.2270	1.2446	1.1408	1.0200	1.4890
Δ*rkp* 040	0.9890	1.0177	0.9842	0.9059	0.9822	0.9435	0.7582	1.0192	0.9994	0.9722	0.9602	1.0496	0.9700	0.9902	0.9477	0.7981	0.9198	1.2954	0.9648	0.8150
Δ*rkp* 043	0.9883	1.1360	1.0203	0.8157	1.0400	1.0570	0.9285	0.9228	1.0225	0.9798	0.9763	1.0040	1.0237	0.8779	0.9650	1.0936	1.1791	1.1379	1.0068	1.2214
Δ*rkp* 045	0.9494	1.4790	1.0340	1.1389	0.9802	0.9968	0.8770	0.8541	1.0321	0.9659	0.8583	0.9954	1.0804	0.6376	0.8751	0.9600	1.2379	0.9491	0.9619	1.2916
Δ*rkp* 046	0.9828	1.0406	0.9649	1.1954	0.9844	0.8879	0.7992	1.0767	1.0209	0.9352	1.0130	1.3052	0.9023	1.0192	0.9953	0.6124	0.8056	1.2516	0.9926	0.8590
Δ*rkp* 047	0.9467	1.3045	0.9992	1.5269	1.0291	1.0255	0.8789	0.8374	1.0466	0.9143	0.9074	1.1242	1.0706	0.8194	0.8935	1.1197	1.2329	1.1792	1.0038	1.2336
Δ*rkp* 059	0.9456	1.0931	0.9565	1.2733	0.9543	0.8938	0.8525	1.3323	1.0237	1.1137	0.8921	1.3902	0.8905	1.1217	0.9487	0.6022	0.6606	0.5876	0.9696	0.8212
Δ*rkp* 060	0.2189	2.6100	0.2226	16.5570	0.8410	0.3353	0.5113	0.2786	1.6790	0.6315	0.0000	0.8643	1.1743	0.3314	0.0000	2.2476	1.5941	1.0368	0.7123	0.2723
Δ*rkp* 062	0.9937	1.0616	1.0398	1.3126	1.0947	1.1002	0.8967	0.7498	1.0089	0.8725	1.0466	1.1041	1.1443	0.8744	0.9785	1.3002	1.3559	1.2429	1.0893	1.4592
Δ*rkp* 064	0.8912	1.2450	0.9290	1.1394	0.8708	0.8067	0.7377	0.9696	1.0821	0.9349	0.7451	0.9882	0.9026	0.6297	0.8570	0.6668	1.0022	0.9258	0.8489	0.8701
Δ*rkp* 066	0.9593	0.9953	0.9632	1.2500	1.0016	0.8634	0.8074	1.2061	1.0332	1.0457	0.9390	0.8876	0.9341	1.1213	0.9278	0.5651	0.7912	1.3866	0.9899	0.8497
Δ*rkp* 067	0.9814	1.0395	1.0309	1.1885	1.0406	1.0090	0.9242	0.9520	1.0152	1.0429	1.0170	1.2085	1.0450	0.9423	1.0030	1.1020	1.1414	1.0400	1.0372	1.2235
Δ*rkp* 068	0.9705	1.0683	1.0504	1.4778	1.0599	1.0859	0.9099	0.9100	0.9847	1.0390	1.0245	0.9814	1.1027	0.9205	1.0063	1.3257	1.1905	1.1077	1.0575	1.4333
Δ*rkp* 076	0.9659	1.0568	0.9640	1.2110	0.9996	0.9009	0.7459	1.0304	1.0358	0.9267	0.9440	0.9072	0.9338	1.0319	0.8956	0.7682	0.9337	0.9582	0.9766	0.9010
Δ*rkp* 079	0.9757	1.0115	0.9737	0.9295	1.0175	0.9054	0.7746	1.0176	1.0409	0.9780	0.9585	1.2375	0.9610	0.9501	0.8966	0.7929	1.0690	1.3409	0.9675	0.9099
Δ*rkp* 081	0.9894	0.9775	0.9596	1.4941	1.0701	1.1075	0.9496	0.8976	1.0266	1.0057	1.0921	0.9701	1.1076	0.9836	0.9807	1.3290	1.2125	1.0858	1.0772	1.4959
Δ*rkp* 082	0.9317	1.2617	0.9528	0.4661	0.9006	0.7867	0.6844	0.8339	1.0104	0.8717	0.7256	1.0924	0.9480	0.6163	0.8812	0.9166	1.1856	0.6491	0.8906	0.8769
Δ*rkp* 085	0.9510	1.0211	0.9613	1.3980	0.9821	0.9317	0.8074	1.2093	1.0408	1.0572	0.9193	0.8899	0.9017	1.0790	0.9407	0.5331	0.7458	1.0542	0.9669	0.8842
Δ*rkp* 088	0.9814	1.2111	1.0514	1.5024	1.0883	1.0954	0.9443	0.8305	0.9546	0.9566	1.0802	1.0193	1.1375	0.9504	0.9733	1.3325	1.3984	1.2828	1.1049	1.4538
Δ*rkp* 089	0.9481	1.3240	1.0182	0.6746	0.9720	0.9778	0.8631	0.9093	1.0183	0.9921	0.8551	1.0439	1.0879	0.6439	0.8770	0.8741	1.1410	0.9644	0.9543	1.2324
Δ*rkp* 092	0.9896	1.0492	0.9921	0.9296	0.9996	0.9515	0.7377	0.8834	1.0349	0.9280	0.9855	0.9780	0.9958	0.9283	0.9262	0.8690	1.0810	1.0739	0.9806	1.0279
Δ*rkp* 106	0.3072	2.0592	0.3099	9.1531	0.7759	0.6368	0.2910	0.2668	1.4103	0.4917	0.0092	1.3246	0.9571	0.1858	0.2494	2.8015	1.8257	1.2961	0.7196	0.6566
Δ*rkp* 107	0.9477	1.1332	0.9622	1.4756	0.9632	0.9288	0.8156	1.1933	1.0481	1.0165	0.9320	0.9217	0.9004	1.0442	0.9336	0.5978	0.8488	1.0485	0.9628	0.9417
Δ*rkp* 112	0.9175	1.3382	0.9572	0.7649	0.8700	0.9212	0.6230	0.6613	1.0137	0.7997	0.8126	1.2259	0.9712	0.4878	0.7398	0.6429	1.5501	1.1342	0.9134	1.1313
Δ*rkp* 117	0.9908	1.0348	1.0182	1.6180	1.0858	1.1154	0.9219	0.8029	0.9755	0.8946	1.0533	1.0502	1.1009	0.8825	0.9527	1.2433	1.4368	1.4222	1.0838	1.3987
Δ*rkp* 120	0.9724	1.1166	0.9884	1.0626	0.9987	0.8101	0.7090	0.7891	1.0225	0.8674	0.9688	0.9467	0.9972	0.8903	0.8282	0.8894	1.2481	0.9685	0.9763	1.0686
Δ*rkp* 125	0.9543	1.1775	0.9638	0.9060	0.9377	0.8522	0.7664	0.9808	1.0910	0.9405	0.8635	1.1609	0.9368	0.9352	0.8867	0.6807	0.9235	0.9346	0.9207	0.8355
Δ*rkp* 132	0.9908	0.9949	1.0467	1.5022	1.0666	1.1080	0.9222	0.9386	1.0454	1.0216	1.0323	1.0168	1.0773	0.8900	0.9399	1.1956	1.3270	1.1578	1.0526	1.3431
Δ*rkp* 135	0.9856	1.0203	1.0377	1.2545	1.0356	1.0502	0.9581	1.0757	1.0857	1.0743	0.9815	1.0296	1.0341	0.9080	0.9343	1.1049	1.0910	1.1478	0.9996	1.1700
Δ*rkp* 142	0.9990	1.0171	0.9930	0.9139	1.0144	0.9798	0.7090	0.7955	1.0446	0.8794	1.0369	0.8965	1.0026	0.8822	0.8861	1.0021	1.2286	0.9508	0.9932	1.1145
Δ*rkp* 144	0.9985	1.0156	1.0298	1.1965	1.0474	1.0282	0.9307	0.9349	0.9979	0.9726	0.9999	1.0527	1.0327	0.9264	0.9360	1.0491	1.1487	1.1559	1.0131	1.1102
Δ*rkp* 148	0.9958	0.9629	1.0219	1.3786	1.0655	1.0591	0.8985	0.8356	0.9775	0.9177	1.0348	1.0360	1.0675	0.8964	0.9450	1.1518	1.3069	1.0379	1.0469	1.2038
Δ*rkp* 149	1.0138	0.9497	1.0541	0.8115	1.0010	0.9705	0.9783	1.0302	1.0696	0.9939	0.9751	0.9819	0.9605	1.0125	0.9670	0.8566	1.0383	0.9576	0.9820	0.8411
Δ*rkp* 151	0.9982	0.9985	1.0588	1.2216	1.0559	1.0099	0.9221	0.9897	1.0325	0.9900	0.9908	1.0232	1.0347	1.0072	0.9330	1.0157	1.1721	1.1157	1.0239	0.9913
Δ*rkp* 152	0.9873	1.0484	0.9800	1.1016	1.0620	0.8459	0.7254	0.9105	1.0352	0.9014	0.9856	0.9586	0.9678	0.9829	0.8874	0.8269	1.0601	1.0192	0.9680	1.0368
Δ*rkp* 153	0.9991	0.9824	1.0145	1.2630	1.0607	1.0344	0.9199	0.9341	1.0211	0.9602	0.9974	0.9713	1.0172	0.9293	0.9166	1.0986	1.2023	1.1306	1.0072	1.1022
Δ*rkp* 161	0.9883	1.5104	1.0441	1.0471	1.0515	1.0283	0.9570	0.9515	0.9932	0.9882	0.9582	1.0230	1.0004	0.8871	0.8972	0.9658	1.2177	1.1636	0.9901	1.0821
Δ*rkp* 162	0.8305	1.4574	0.8638	1.6311	0.8689	0.8984	0.6025	0.5495	1.0339	0.6816	0.7274	1.0247	0.9187	0.3948	0.6741	0.8809	1.7123	1.0518	0.8729	0.9995
Δ*rkp* 164	1.0010	1.0103	1.0116	0.8747	1.0526	0.8725	0.6680	0.7268	1.0449	0.8534	1.0984	0.9790	1.0287	0.8271	0.8825	1.0891	1.3686	1.7666	1.0226	1.2618
Δ*rkp* 165	0.9954	1.0428	1.0387	0.9843	1.0365	0.9203	0.6803	0.7764	1.0567	0.8572	1.0378	1.0045	0.9934	0.8322	0.9003	0.8995	1.2728	1.0229	0.9919	1.0280
Δ*rkp* 167	0.9909	1.0035	1.0251	1.2548	1.0357	1.0430	0.9877	1.0186	1.0153	1.0365	1.0089	1.0180	0.9816	0.9806	0.9302	1.0800	1.0731	1.1857	0.9933	1.1309
Δ*rkp* 168	0.9865	0.9915	1.0024	0.9352	1.0559	0.9925	0.9514	1.1105	1.0770	1.0653	0.9813	0.7196	1.0359	0.9964	0.9550	1.2426	1.1526	1.0833	1.0131	0.9593
Δ*rkp* 171	0.9643	1.1410	1.0559	0.8747	0.9673	0.9129	0.6557	0.7508	1.0641	0.8547	0.9617	1.0497	0.9667	0.7672	0.8741	0.9090	1.3461	1.2970	0.9685	1.0998
Δ*rkp* 175	0.2422	1.9719	0.2543	16.8484	0.9401	0.6296	0.4720	0.3566	1.9560	0.6193	0.0297	1.1219	1.1434	0.3474	0.0000	1.6574	2.3716	1.5032	0.8228	0.6630
Δ*rkp* 177	0.9841	1.0528	1.0388	1.2545	1.0551	1.0611	0.8660	0.8194	0.9918	0.9150	0.9531	1.0175	0.9848	0.8454	0.8894	0.9465	1.3057	1.1350	0.9875	1.1901
Δ*rkp* 178	0.9940	1.0574	1.0509	1.1389	1.0482	1.0601	0.8772	0.7957	0.9538	0.9513	0.9919	0.9845	1.0441	0.7844	0.9118	1.1586	1.4056	1.1769	1.0227	1.2558
Δ*rkp* 184	1.0359	0.8989	1.0060	0.9609	1.0585	1.0426	0.6393	0.7300	1.0536	0.8210	1.1748	0.8224	1.0402	0.9192	0.9287	1.1510	1.3824	1.3450	1.0560	1.2384
Δ*rkp* 185	0.9982	1.0200	1.0346	1.2131	1.0285	1.0392	0.9005	0.9041	0.9920	0.9646	0.9987	0.9872	1.0029	0.8553	0.9176	1.0565	1.1785	1.0386	0.9895	1.1547
Δ*rkp* 188	0.8922	1.3487	0.9897	2.3933	1.0312	1.0112	0.9273	1.0020	1.0913	1.0704	0.7822	1.1023	0.9983	0.7018	0.8018	0.9474	1.2127	0.9586	0.9219	1.2221
Δ*rkp* 194	0.9927	1.0409	0.9918	0.9232	1.0519	1.0111	0.8542	0.8388	0.9702	0.9402	0.9705	1.0161	1.0145	0.7881	0.9065	0.9909	1.2956	1.0957	0.9943	1.1138
Δ*rkp* 195	0.9972	0.9706	1.0309	1.2709	1.0507	1.0739	0.8995	0.8382	0.9695	0.9547	1.0425	0.9708	1.0306	0.8564	0.9326	1.1196	1.2809	1.2746	1.0245	1.2787
Δ*rkp* 212	1.0228	0.9693	0.9943	0.9608	1.0474	0.9779	0.6557	0.7188	1.0490	0.7939	1.0744	0.9778	1.0158	0.8729	0.9073	0.9939	1.2600	0.9588	1.0097	1.0295
Δ*rkp* 223	0.9794	1.0340	0.9931	1.0156	1.0065	0.9633	0.7377	0.9153	1.0752	0.9934	0.9927	1.0164	0.9803	0.9159	0.9469	0.8595	1.0662	1.3311	0.9777	0.8718
Δ*rkp* 224	0.9979	0.9671	0.9902	1.2078	1.0599	1.0395	0.9077	0.8004	1.0063	0.9214	1.0217	0.9651	1.0147	0.8964	0.9613	0.9979	1.3568	1.1522	1.0340	1.1790
Δ*rkp* 231	1.0438	0.8464	1.0350	0.9217	1.0685	1.0620	0.6434	0.7188	1.0489	0.7964	1.2287	0.8907	1.0262	0.9342	0.9132	1.1731	1.3404	1.7846	1.0537	1.2500
Δ*rkp* 237	1.0043	0.9974	1.0425	1.1546	1.0647	1.0358	0.8648	0.8067	0.9790	0.8963	0.9985	0.9647	1.0240	0.8528	0.9088	1.0518	1.2950	1.1866	1.0101	1.2232
Δ*rkp* 247	0.9887	1.0112	0.9881	1.2545	1.0487	1.0246	0.9164	0.8939	1.0296	0.9970	1.0330	0.9790	1.0291	0.9621	0.9481	1.1119	1.2160	1.3856	1.0318	1.1478
Δ*rkp* 249	0.9985	1.1503	1.0509	1.2703	1.0703	1.0919	0.8522	0.7426	1.0006	0.9024	1.0470	0.9513	1.0360	0.8074	0.9089	1.1605	1.3138	1.1414	1.0189	1.3044
Δ*rkp* 250	0.9856	0.9520	1.0446	1.2956	1.0362	1.1061	0.8958	0.8351	1.0034	1.0234	1.0084	0.9461	0.9815	0.8010	0.8875	1.1346	1.1327	1.1329	0.9646	1.3313
Δ*rkp* 256	0.9978	0.9867	1.0129	1.0061	1.0287	1.0125	1.0105	0.9274	1.0072	0.9756	1.0161	1.0191	1.0055	0.9102	0.9316	0.9647	1.1289	1.0141	0.9975	0.9110
Δ*rkp* 272	1.0384	0.9211	1.0237	0.7569	1.1407	0.8769	0.6721	0.7716	1.0571	0.8439	1.1478	0.9639	1.0075	0.8798	0.9493	0.9642	1.2370	1.1016	1.0225	1.0020
Δ*rkp* 273	1.0147	1.0302	1.0133	0.6314	0.9882	0.9020	0.6762	0.7444	1.0289	0.8557	1.0730	0.9634	1.0094	0.8071	0.8887	0.9210	1.2576	1.0527	0.9936	1.0588
Δ*rkp* 274	1.0465	0.8001	1.0415	0.7963	1.0437	1.0263	0.7008	0.8642	1.0754	0.9027	1.2266	0.9556	0.9909	0.9515	0.9245	1.0105	1.2761	1.0944	1.0381	1.0657
Δ*rkp* 282	0.9878	0.8451	1.0520	1.1880	1.0353	1.0711	0.8625	0.8061	0.9801	0.9394	1.0297	0.9904	1.0471	0.8535	0.9169	1.1326	1.2496	1.1268	1.0196	1.2151
Δ*rkp* 288	1.0274	0.8803	1.0200	0.8041	1.0311	1.0344	0.6598	0.8019	1.1286	0.8672	1.1629	1.0667	1.0198	0.8341	0.9009	1.0383	1.3311	0.9516	1.0272	1.2352
Δ*rkp* 316	0.9913	0.8904	0.9618	1.0722	1.0429	1.0706	0.8326	0.7334	0.9706	0.8665	1.0211	0.8830	1.0316	0.7798	0.8826	1.0583	1.4064	1.1841	1.0117	1.2844
Δ*rkp* 317	0.9962	0.7905	0.9549	1.2706	1.0482	1.0972	0.9552	0.8256	0.9720	0.9638	1.0939	0.9624	1.0696	0.8851	0.9521	1.2563	1.2736	1.1983	1.0522	1.3279
Δ*rkp* 318	1.0168	0.8827	1.0420	0.8590	1.0120	1.0034	0.6311	0.7013	0.9678	0.8349	1.1320	0.9918	1.0025	0.9238	0.8934	0.9376	1.1571	1.0044	1.0055	0.9735
Δ*rkp* 328	1.0575	0.7830	1.0465	0.7884	1.0278	1.0348	0.5820	0.5383	1.0337	0.7141	1.2534	0.9842	1.0196	0.8904	0.8996	1.1649	1.3150	1.7049	1.0462	1.1734
Δ*rkp* 346	0.9913	0.8267	1.0293	1.1547	1.0330	1.0406	0.9315	0.9443	0.9844	1.0259	1.0358	0.9883	1.0395	0.9191	0.9504	1.1042	1.1805	1.1270	1.0225	1.1434

	Amino acids in sake (continued)																			
	Thr	Ser	Asn	Glu	Gln	Gly	Ala	Val	Cys	Met	Ile	Leu	Tyr	Phe	His	Lys	Trp	Arg	Pro	Total amino acid
Control	1.0000	1.0000	1.0000	1.0000	1.0000	1.0000	1.0000	1.0000	1.0000	1.0000	1.0000	1.0000	1.0000	1.0000	1.0000	1.0000	1.0000	1.0000	1.0000	1.0000
Δ*rkp* 002	1.3967	1.5485	1.2621	1.3603	1.5355	1.4867	1.3077	1.1219	0.9220	2.4911	1.2879	1.3683	1.2812	1.3129	0.8986	1.3871	1.9885	1.2653	1.2987	1.3455
Δ*rkp* 005	0.8720	0.8908	0.7766	0.9503	0.8821	0.9351	0.8878	0.8818	0.6986	0.8797	0.9274	0.9277	0.8628	0.8700	0.8491	1.0091	0.8026	1.0197	0.8540	0.9187
Δ*rkp* 007	0.9064	0.9038	0.9146	0.8770	0.8905	0.9727	0.9457	0.9727	0.9784	0.9526	0.9156	0.8882	0.9543	0.9158	1.0012	0.9168	0.9847	1.0431	0.9891	0.9484
Δ*rkp* 009	0.9649	0.9921	0.9571	0.9917	0.9939	1.0127	0.9944	0.9863	0.9780	0.9575	0.9804	0.9700	0.9876	0.9521	0.9801	1.0149	0.7331	1.0081	1.0540	0.9943
Δ*rkp* 016	0.9114	0.9061	0.9115	0.8662	0.8785	0.9809	0.9591	0.9867	0.9538	0.9546	1.0238	0.9144	0.9819	0.9173	0.9960	0.9191	0.9115	1.0547	0.9997	0.9597
Δ*rkp* 017	1.2007	1.2391	1.1192	1.1938	1.1628	1.0671	1.0150	1.1328	1.1015	1.3714	1.1881	1.1839	1.0934	1.1637	1.0534	1.1572	1.2997	1.1334	1.0059	1.1274
Δ*rkp* 019	0.8817	0.9002	0.8047	0.9309	0.9073	0.9567	0.8875	0.9204	0.7563	0.8400	0.9571	0.9459	0.8926	0.9107	0.8000	0.9897	0.8165	1.0184	0.8990	0.9303
Δ*rkp* 021	0.9235	0.9286	0.9178	0.9535	0.9155	0.9697	0.9930	0.9732	0.9465	0.5857	0.9712	0.9716	0.9885	0.9682	0.9224	0.9599	0.7068	1.0154	0.9915	0.9702
Δ*rkp* 022	1.0982	1.1155	1.0744	1.1004	1.0748	1.0390	0.9831	1.0683	1.0382	1.2193	1.2030	1.1409	1.0805	1.0911	1.0986	1.1386	1.1819	1.1343	0.9960	1.0833
Δ*rkp* 024	0.9264	0.9462	0.8338	1.1252	1.0181	0.9477	0.8746	1.0475	0.5494	0.9230	1.1980	1.0976	0.9198	1.0251	0.5601	1.1366	1.2690	0.9959	0.8101	0.9812
Δ*rkp* 025	1.0117	1.0195	0.9893	0.9739	0.9830	1.0334	1.0046	1.0089	1.0495	1.1709	1.0832	1.0050	1.0629	1.0129	1.0806	1.0499	1.0926	1.1055	1.0457	1.0332
Δ*rkp* 027	0.9627	0.9798	0.9193	0.9634	0.9431	0.9846	0.9292	0.9780	0.7941	1.0450	0.9474	0.9545	0.9771	0.9581	1.0168	1.0198	0.9852	1.0727	0.9693	0.9786
Δ*rkp* 031	1.0705	1.0980	1.0511	1.1409	1.0068	1.0377	0.9933	1.1392	0.9972	1.1702	1.1352	1.1467	1.0884	1.1225	1.0947	1.1691	1.4105	1.0930	1.0217	1.0833
Δ*rkp* 032	1.1184	1.1439	1.0775	1.1643	1.0314	1.0464	0.9892	1.1766	1.0519	1.2754	1.2667	1.1970	1.1418	1.1462	1.1255	1.2007	1.4133	1.1467	1.0240	1.1169
Δ*rkp* 033	1.2055	1.2504	1.1440	1.2306	1.1104	1.0893	1.0477	1.0712	0.9685	1.4209	1.3277	1.2412	1.1465	1.1670	1.2042	1.2907	1.4304	1.1225	1.0265	1.1491
Δ*rkp* 040	0.8982	0.8979	0.9143	0.9101	0.8990	0.9022	0.9397	0.9241	0.8556	0.8410	0.9104	0.9148	0.9248	0.9019	0.8687	0.9173	1.1278	0.9366	0.9434	0.9182
Δ*rkp* 043	1.0828	1.1078	1.0624	1.1254	1.0044	1.0293	1.0062	1.0208	0.7718	1.2366	1.2171	1.1412	1.0800	1.0714	1.1501	1.1796	1.3729	1.0751	0.9949	1.0704
Δ*rkp* 045	0.9895	1.0183	0.9428	1.0747	0.9080	0.9985	0.9439	1.0367	0.7411	0.9828	1.0935	1.0671	0.9722	0.9927	0.9792	1.1716	0.9800	1.0631	0.8923	1.0120
Δ*rkp* 046	0.9116	0.9316	0.9269	0.9260	0.9490	0.9636	1.0005	0.9567	0.9214	0.8393	0.9585	0.9428	0.9563	0.9304	0.9005	0.9130	0.9942	0.9462	1.0229	0.9545
Δ*rkp* 047	1.0116	1.0190	0.9192	1.0589	0.9681	0.9716	0.9350	1.0904	0.8386	1.0410	1.1964	1.0917	1.0236	1.0211	0.9651	1.1484	1.3659	1.0880	0.9025	1.0267
Δ*rkp* 059	0.8653	0.8829	0.8872	0.9064	0.8800	0.9235	0.9721	0.8975	0.7838	0.7828	0.9123	0.8883	0.9131	0.8709	0.8076	0.9049	0.5834	0.9394	0.9834	0.9161
Δ*rkp* 060	0.2199	0.3145	0.2604	0.3080	0.4582	0.2345	0.2489	0.2040	0.0000	0.3226	0.2499	0.2535	0.2069	0.2090	0.0000	0.3936	0.0000	0.3239	0.1792	0.2618
Δ*rkp* 062	1.1898	1.2269	1.1382	1.2377	1.0903	1.1163	1.0511	1.2313	1.0932	1.3532	1.3279	1.2539	1.1486	1.1916	1.2272	1.2802	1.3930	1.0849	1.0490	1.1588
Δ*rkp* 064	0.8215	0.7891	0.7572	0.8361	0.8251	0.8163	0.8236	0.8219	0.5197	0.6639	0.8633	0.8155	0.7637	0.7571	0.5874	0.8347	0.4758	0.8583	0.7946	0.8143
Δ*rkp* 066	0.8756	0.8860	0.8761	0.8857	0.8496	0.9247	0.9803	0.9224	0.8729	0.8862	0.9186	0.8896	0.9187	0.8891	0.9409	0.8805	0.8164	0.9457	0.9636	0.9205
Δ*rkp* 067	1.0359	1.0417	0.9473	1.0443	0.9578	0.9897	0.9651	1.0717	0.9777	1.1198	1.0564	1.0555	1.0216	1.0340	1.0399	1.1399	1.2371	1.1212	0.9765	1.0373
Δ*rkp* 068	1.1699	1.2039	1.1086	1.2249	1.0598	1.0880	1.0371	1.2702	1.0638	1.3237	1.3230	1.2411	1.1524	1.1804	1.2100	1.2631	1.4605	1.1089	1.0516	1.1530
Δ*rkp* 076	0.8711	0.8910	0.8394	0.9089	0.8471	0.9209	0.9294	0.9349	0.8083	0.9052	0.9369	0.9336	0.9324	0.9177	0.8999	0.9659	0.9131	0.9953	0.9840	0.9337
Δ*rkp* 079	0.8978	0.8953	0.8555	0.9194	0.8506	0.9040	0.9211	0.9072	0.8404	0.9574	0.9052	0.9050	0.9058	0.8817	0.8219	0.9422	0.8598	0.9630	0.9342	0.9148
Δ*rkp* 081	1.2295	1.2748	1.1585	1.2946	1.0764	1.0917	1.0456	1.2962	1.1512	1.4866	1.3749	1.2664	1.1724	1.1546	1.2400	1.3419	1.5861	1.1457	1.0844	1.1848
Δ*rkp* 082	0.8054	0.7680	0.7598	0.8224	0.7250	0.7695	0.8017	0.8452	0.5587	0.7084	0.8678	0.8138	0.7714	0.7584	0.6791	0.8448	0.4614	0.8616	0.7816	0.8048
Δ*rkp* 085	0.9137	0.9413	0.9465	0.9310	0.8859	0.9802	1.0036	0.9867	0.9364	0.8993	0.9824	0.9619	0.9858	0.9505	0.9656	0.9421	0.8827	0.9838	1.0145	0.9694
Δ*rkp* 088	1.1781	1.2281	1.1132	1.1983	1.0935	1.0661	0.9924	1.2082	0.8647	1.3748	1.2936	1.2116	1.1226	1.1379	1.2000	1.2857	1.4997	1.1357	1.0329	1.1370
Δ*rkp* 089	0.9566	0.9799	0.9012	1.0294	0.8740	0.9770	0.9065	1.0798	0.6518	0.9514	1.0472	1.0304	0.9449	0.9589	0.9350	1.1611	1.0352	1.0490	0.8999	0.9877
Δ*rkp* 092	0.9989	1.0071	1.0011	0.9871	0.9429	0.9675	0.9896	1.0007	0.9598	1.0545	0.9996	0.9962	0.9813	0.9586	0.9748	0.9840	1.1817	0.9913	0.9947	0.9881
Δ*rkp* 106	0.6511	0.7321	0.5264	0.7129	0.7605	0.4548	0.5121	0.5249	0.0712	1.0973	0.6738	0.5862	0.4568	0.5418	0.1077	0.7095	0.6735	0.5040	0.3922	0.5464
Δ*rkp* 107	0.9223	0.9615	0.9130	0.9843	0.7861	0.9792	1.0011	0.9905	0.9380	0.9402	1.0025	0.9884	0.9847	0.9733	0.9445	0.9959	0.8933	1.0191	1.0099	0.9802
Δ*rkp* 112	0.9494	0.9126	0.8478	0.8843	0.9107	0.8231	0.8434	0.9264	0.5802	0.8161	0.9839	0.9009	0.8170	0.8465	0.7274	0.9358	0.6364	0.8557	0.7890	0.8695
Δ*rkp* 117	1.1475	1.1848	1.0838	1.1711	1.1287	1.0656	1.0097	1.1914	0.9004	1.3429	1.2063	1.2027	1.1304	1.1768	1.2048	1.2609	1.4519	1.1410	1.0502	1.1338
Δ*rkp* 120	0.9656	0.9760	0.9543	0.9584	0.9858	0.9516	0.9521	1.0127	0.9449	1.0576	1.0136	1.0082	0.9749	0.9874	1.0082	1.0073	1.1651	1.0171	0.9943	0.9888
Δ*rkp* 125	0.8055	0.8030	0.7911	0.8415	0.8025	0.8426	0.8723	0.8666	0.7980	0.7698	0.8652	0.8638	0.8469	0.8311	0.7228	0.8475	0.5892	0.9096	0.8783	0.8570
Δ*rkp* 132	1.1305	1.1569	1.0711	1.1179	1.1004	1.0673	1.0101	1.1810	1.0522	1.3065	1.1733	1.1631	1.1033	1.1445	1.1799	1.2017	1.4114	1.0805	1.0317	1.1073
Δ*rkp* 135	1.0558	1.0670	1.0195	1.0574	1.0515	1.0060	0.9993	1.0671	0.9708	1.1743	1.0622	1.0642	1.0046	1.0043	1.0800	1.1495	1.1485	0.9996	0.9821	1.0333
Δ*rkp* 142	1.0323	1.0480	1.0179	0.9945	1.0485	0.9869	0.9950	1.0115	1.0082	1.0902	1.0075	1.0118	0.9721	0.9771	0.9807	1.0240	1.2921	1.0036	1.0022	1.0075
Δ*rkp* 144	1.0501	1.0603	1.0370	1.0488	1.0368	1.0130	0.9840	1.0928	1.0314	1.1815	1.0776	1.0778	1.0554	1.0469	1.1302	1.1257	1.3275	1.0336	1.0209	1.0453
Δ*rkp* 148	1.0713	1.0933	1.0361	1.0566	1.0500	1.0203	0.9904	1.1197	1.0134	1.2338	1.1022	1.1014	1.0579	1.0669	1.1381	1.1337	1.3206	1.0167	1.0163	1.0553
Δ*rkp* 149	0.9442	0.9341	0.9635	0.9207	0.9799	0.9701	0.9859	0.9455	0.9896	1.0602	0.9322	0.9206	0.9616	0.8997	1.0046	0.9807	0.8534	0.8675	0.9966	0.9430
Δ*rkp* 151	0.9992	1.0015	0.9879	0.9874	1.0363	1.0223	1.0033	1.0315	0.9971	1.1097	1.0282	1.0320	1.0274	1.0097	1.0994	1.0757	1.0872	0.8947	1.0245	1.0019
Δ*rkp* 152	0.9633	0.9179	0.8942	0.9366	0.9350	0.8849	0.8927	0.9928	0.7581	0.9470	1.0322	0.9760	0.8901	0.9289	0.8559	0.9907	1.0471	0.9263	0.8923	0.9302
Δ*rkp* 153	1.0524	1.0576	1.0261	1.0271	1.0872	1.0190	0.9954	1.0409	0.8379	1.1866	1.0573	1.0710	1.0372	1.0349	1.0959	1.1383	1.1826	1.0316	1.0162	1.0388
Δ*rkp* 161	1.0198	1.0237	0.9925	0.9924	1.0412	1.0007	0.9774	0.9326	0.7195	1.1289	1.0402	1.0451	1.0220	1.0107	1.0593	1.1177	1.2824	1.0299	0.9950	1.0113
Δ*rkp* 162	0.8881	0.8528	0.8049	0.8701	0.8568	0.8008	0.8308	0.9198	0.6474	0.9201	0.9947	0.8976	0.8095	0.8500	1.0019	0.9633	0.8506	0.8321	0.8035	0.8572
Δ*rkp* 164	1.1517	1.1445	1.1234	1.0662	1.1343	1.0069	1.0348	1.0624	1.0582	1.3353	1.0665	1.0700	1.0079	1.0322	1.1008	1.0984	1.2977	1.0359	1.0275	1.0611
Δ*rkp* 165	0.9677	0.9657	0.9436	0.9112	0.9090	0.8975	0.9168	0.9442	0.9070	1.0688	0.9381	0.9295	0.8956	0.9083	0.8810	0.9409	1.0661	0.9219	0.9109	0.9243
Δ*rkp* 167	1.0975	1.1021	1.0675	1.0483	1.1147	1.0403	1.0087	1.0921	0.8685	1.2431	1.1510	1.1115	1.0803	1.0622	1.1217	1.1673	1.3922	1.0659	1.0383	1.0715
Δ*rkp* 168	1.0249	1.0195	1.0341	0.9949	1.0146	1.0116	0.9582	1.0574	0.9873	1.1449	0.9910	0.9970	0.9890	0.9624	1.1016	1.0868	0.7613	1.0330	1.0016	1.0068
Δ*rkp* 171	0.9665	0.9811	0.9338	0.9558	0.9605	0.9355	0.9146	0.9640	0.7088	1.0365	0.9968	0.9984	0.9236	0.9567	0.8923	1.0265	0.7200	0.9593	0.9203	0.9542
Δ*rkp* 175	0.6329	0.7893	0.5601	0.5925	1.0237	0.4414	0.4267	0.4190	0.2086	0.9328	0.4563	0.5289	0.3965	0.4193	0.1808	0.7409	0.0000	0.5865	0.5789	0.5398
Δ*rkp* 177	1.0558	1.0746	1.0161	1.0225	1.0645	1.0121	0.9870	1.0998	0.9584	1.1349	1.0897	1.0890	1.0546	1.0755	1.0594	1.1702	1.0672	1.0723	1.0006	1.0532
Δ*rkp* 178	1.0702	1.0964	1.0318	1.0256	1.1617	1.0236	0.9575	1.0591	1.0090	1.1878	1.1058	1.0995	1.0463	1.0908	1.0890	1.2408	1.1783	1.1276	0.9686	1.0664
Δ*rkp* 184	1.2045	1.1977	1.1770	1.0871	1.1457	1.0424	1.0667	1.0789	1.1547	1.4592	1.0788	1.0727	1.0272	1.0445	1.1206	1.1025	1.0886	1.0344	1.0507	1.0792
Δ*rkp* 185	1.0727	1.0927	1.0404	1.0276	1.1427	1.0159	0.9790	1.0546	0.9796	1.1899	1.1807	1.1078	1.0664	1.0693	1.1158	1.1431	1.3119	1.0186	1.0046	1.0524
Δ*rkp* 188	0.9785	0.9991	0.8822	1.0217	1.0906	0.9705	0.9264	1.0135	0.7880	1.1566	1.0591	1.0562	0.9667	1.0112	0.8257	1.1752	1.1623	1.0687	0.9194	1.0077
Δ*rkp* 194	1.0116	1.0163	0.9426	0.9239	1.0446	0.9539	0.9252	0.9839	0.8983	1.0799	1.0733	1.0173	0.9967	0.9808	0.9977	1.0754	0.8917	1.0537	0.9230	0.9903
Δ*rkp* 195	1.1593	1.1869	1.1096	1.0805	1.1971	1.0792	1.0481	1.1648	1.0784	1.3025	1.1411	1.1392	1.0979	1.1195	1.2041	1.1916	1.3657	1.0473	1.0396	1.1075
Δ*rkp* 212	1.0048	1.0032	0.9919	0.9415	0.9420	0.8933	0.9376	0.9325	0.9310	1.1662	0.9358	0.9329	0.8819	0.8958	0.8905	0.9370	0.7167	0.9067	0.9256	0.9297
Δ*rkp* 223	0.8651	0.8685	0.8606	0.8351	0.8102	0.7999	0.8323	0.8284	0.7643	0.9439	0.8397	0.8434	0.8060	0.8138	0.7934	0.8273	0.4575	0.8287	0.8386	0.8293
Δ*rkp* 224	1.1000	1.1210	1.0643	1.0369	1.1213	1.0707	1.0478	1.1667	1.0406	1.2866	1.2526	1.1312	1.1260	1.1002	1.1667	1.0980	1.2549	0.9711	1.0301	1.0807
Δ*rkp* 231	1.2497	1.2372	1.2218	1.1038	1.1698	1.0476	1.0996	1.0741	1.1770	1.4637	1.0694	1.0672	1.0323	1.0412	1.1080	1.1429	1.1729	1.0447	1.0794	1.0938
Δ*rkp* 237	1.0867	1.1135	1.0421	1.0183	1.1280	1.0399	1.0036	1.1193	1.0177	0.9260	1.0945	1.0911	1.0588	1.0746	1.1601	1.1327	1.1327	1.0306	0.9874	1.0580
Δ*rkp* 247	1.0751	1.0884	1.0483	1.0142	1.1034	1.0296	0.9899	1.1107	1.0070	1.1256	1.0727	1.0753	1.0562	1.0590	1.1502	1.1295	1.3089	1.0796	0.9954	1.0569
Δ*rkp* 249	1.1701	1.1982	1.1095	1.0753	1.1731	1.0739	1.0487	1.1408	1.0500	1.3121	1.2535	1.1751	1.1172	1.1463	1.1390	1.1396	1.3744	0.9694	1.0177	1.0985
Δ*rkp* 250	1.1932	1.2244	1.1475	1.1251	1.1907	1.0816	1.0656	1.1258	0.8879	1.3000	1.2742	1.1978	1.1258	1.1485	1.1621	1.1426	1.4026	0.9824	1.0446	1.1131
Δ*rkp* 256	0.9130	0.9112	0.9013	0.8701	0.9226	0.8959	0.9040	0.9135	0.8542	0.9299	0.9132	0.9220	0.9093	0.9102	0.9212	0.9344	0.9077	0.8554	0.8781	0.8978
Δ*rkp* 272	1.0091	0.9886	1.0098	0.8923	0.9841	0.8912	0.9047	0.8821	0.9400	1.1751	0.9012	0.8936	0.8442	0.8383	0.8851	0.9211	0.6110	0.8793	0.8590	0.8988
Δ*rkp* 273	0.9737	0.9693	0.9483	0.9063	0.9836	0.8951	0.9241	0.9022	0.8831	1.0498	0.9039	0.9175	0.8729	0.8458	0.8080	0.9623	0.7324	0.9464	0.9278	0.9246
Δ*rkp* 274	1.1947	1.1598	1.2019	1.0122	1.1639	1.0197	1.0832	1.0223	1.1660	1.4525	1.0215	1.0056	0.9896	0.9675	1.0810	1.0797	1.0093	1.0160	1.0530	1.0500
Δ*rkp* 282	1.1154	1.1321	1.0781	1.0678	1.0956	1.0657	1.0411	1.0956	0.8600	1.2045	1.2155	1.1516	1.1032	1.1193	1.1204	1.0957	1.3234	0.9745	1.0364	1.0755
Δ*rkp* 288	1.2187	1.1963	1.1953	1.0593	1.1786	1.0061	1.0833	1.0505	1.0549	1.4809	1.0611	1.0536	1.0116	0.9986	1.0174	1.0997	0.9015	1.0176	1.0531	1.0672
Δ*rkp* 316	1.1147	1.1423	1.0548	1.0353	1.0985	1.0576	1.0152	1.1033	0.9666	1.1693	1.1328	1.1026	1.0562	1.0956	1.1104	1.1248	1.2932	1.0378	1.0025	1.0694
Δ*rkp* 317	1.1781	1.2073	1.1209	1.0858	1.1479	1.0639	1.0454	1.1259	1.0818	1.3231	1.2472	1.1762	1.1226	1.1394	1.1482	1.1363	1.4088	1.0383	1.0418	1.1090
Δ*rkp* 318	1.0271	1.0172	1.0429	0.9481	1.0087	0.9212	0.9742	0.9345	0.9999	1.1996	0.9286	0.9317	0.9082	0.8829	0.9113	0.9596	1.0831	0.9064	0.9754	0.9478
Δ*rkp* 328	1.2517	1.2287	1.2219	1.0614	1.1088	1.0535	1.1088	1.0397	1.1581	1.5681	1.0475	1.0454	1.0070	1.0041	1.0771	1.0877	0.7643	0.9813	1.0766	1.0670
Δ*rkp* 346	1.0825	1.0957	1.0590	1.0183	1.0527	1.0443	1.0332	1.0643	1.0097	1.1796	1.1545	1.1042	1.0764	1.0660	1.0812	1.0844	1.2152	0.9705	1.0238	1.0504

Values shown represent mean relative values calculated from independently obtained disruptant strains and are expressed relative to the control strain. Measures of variance are not shown.
