# Supplementary material for: Effects of Aspergillus oryzae-derived rice-koji protein on the sake metabolome
Source: Appl Environ Microbiol. 2026 Feb 19;92(3):e01955-25. doi: 10.1128/aem.01955-25 (PMC12997762; doi:10.1128/aem.01955-25)
Supplement: Table S6 — Composition of glucose minimal medium. [file aem.01955-25-s0007.pdf]

Table S6. Composition of glucose minimal medium

Component	Amount per 1 L
Glucose	30 g
L-glutamic acid	3 g
KCl	0.52 g
K_2HPO_4	1.52 g
L-Methionine	1.5 g
$MgSO_4$	2.1 mL (1M stock)
Trace elements	1.0 mL

The pH of the medium was adjusted to 6.5 before autoclaving.
$MgSO_4$ was sterilized separately and added aseptically after autoclaving.
The trace element solution consisted of 1.0 g/L $FeSO_4 \cdot 7H_2O$, 8.8 g/L $ZnSO_4 \cdot 7H_2O$, 0.4 g/L $CuSO_4 \cdot 5H_2O$, 0.15 g/L $MnSO_4 \cdot 4H_2O$, 0.1 g/L $Na_2B_4O_7 \cdot 10H_2O$, and 0.05 g/L $(NH_4)_6Mo_7O_{24} \cdot 4H_2O$.
For agar plates, agar was added at a final concentration of 20 g/L.
