# Supplementary material for: Effects of Aspergillus oryzae-derived rice-koji protein on the sake metabolome
Source: Appl Environ Microbiol. 2026 Feb 19;92(3):e01955-25. doi: 10.1128/aem.01955-25 (PMC12997762; doi:10.1128/aem.01955-25)
Supplement: Table S7 — Primers used for generation of gene disruption fragments. [file aem.01955-25-s0008.pdf]

Table S7. Primers used for generation of gene disruption fragments

Gene	Primer name	Sequence (5′–3′)	Purpose
rkp 002	rkp-002-A	GCGGGTCACTCATTGGCCAC	Amplification of 5′ flanking region
	rkp-002-C	CTGTGGACTCGTATGCTGTTGCGCAGTGCCTGTCTAAATGCGACCA	
	rkp-002-B	CTGACCTATCTTCCTGGACATGACGGACTCGCAGCAGTTTTCCGCA	Amplification of 3′ flanking region
	rkp-002-D	TGGTCAAGCGAGACTCCATC	
rkp 005	rkp-005-A	GGACGACGTGCATGGAAGTG	Amplification of 5′ flanking region
	rkp-005-C	CTGTGGACTCGTATGCTGTTGCGCAGAGGATCCGTACGGAGGTGGA	
	rkp-005-B	CTGACCTATCTTCCTGGACATGACGGCGGACGGAAAGGAGAAACGG	Amplification of 3′ flanking region
	rkp-005-D	AGAGTGACCACGGCGAACAA	
rkp 007	rkp-007-A	GTCGGGCAAATTGTGGAGAC	Amplification of 5′ flanking region
	rkp-007-C	CTGTGGACTCGTATGCTGTTGCGCAGTGATGGGGTAGGGAAAGGGT	
	rkp-007-B	CTGACCTATCTTCCTGGACATGACGGATAATGTCGGCGAAACACCC	Amplification of 3′ flanking region
	rkp-007-D	GCCAAAGTACGGCTGACTGA	
rkp 009	rkp-009-A	TGGTGTACGTCCGTCGTTAG	Amplification of 5′ flanking region
	rkp-009-C	CTGTGGACTCGTATGCTGTTGCGCAGACCCGGCATTAGACGATCAC	
	rkp-009-B	CTGACCTATCTTCCTGGACATGACGGCGAGGGCAGTACTTGCAAGA	Amplification of 3′ flanking region
	rkp-009-D	CAATGGCCATGAGCCAACAC	
rkp 010	rkp-010-A	GGATGAGGAGGTGGGGACAA	Amplification of 5′ flanking region
	rkp-010-C	CTGTGGACTCGTATGCTGTTGCGCAGATGATGTTGTGCGCGAGGTT	
	rkp-010-B	CTGACCTATCTTCCTGGACATGACGGTGGGGAGCGAAGAAAGAGGG	Amplification of 3′ flanking region
	rkp-010-D	GCCTAGTGCCATGCGTCTTG	
rkp 011	rkp-011-A	CTCGTAATAGGGCTGTGCAA	Amplification of 5′ flanking region
	rkp-011-C	CTGTGGACTCGTATGCTGTTGCGCAGCTCTGCGAGAGGTAGCATGA	
	rkp-011-B	CTGACCTATCTTCCTGGACATGACGGTCCATACTGATCACCACATC	Amplification of 3′ flanking region
	rkp-011-D	GCAGATGGGCGAGAGCTTAC	
rkp 016	rkp-016-A	CCCGTTATGGAGTGGGACGA	Amplification of 5′ flanking region
	rkp-016-C	CTGTGGACTCGTATGCTGTTGCGCAGTCTTCAGCTTCCACCGACAC	
	rkp-016-B	CTGACCTATCTTCCTGGACATGACGGAGAAGAACGCCCACAAGCAG	Amplification of 3′ flanking region
	rkp-016-D	CCATGCACCACAAATCCACC	
rkp 017	rkp-017-A	GGATTACGGCGACGCATTGA	Amplification of 5′ flanking region
	rkp-017-C	CTGTGGACTCGTATGCTGTTGCGCAGGATTGCTCCGGCGAGTCAGG	
	rkp-017-B	CTGACCTATCTTCCTGGACATGACGGGGAAGTCGGATCCGTTCCCT	Amplification of 3′ flanking region
	rkp-017-D	ACAAGAGCCCCAACCGTGAG	
rkp 019	rkp-019-A	GGGTGACCTGCAGTTGGAAG	Amplification of 5′ flanking region
	rkp-019-C	CTGTGGACTCGTATGCTGTTGCGCAGTGATTTGCAGAACTTGGGAG	
	rkp-019-B	CTGACCTATCTTCCTGGACATGACGGGAGGGCAGAGATTGTGCGAG	Amplification of 3′ flanking region
	rkp-019-D	TGATCGATTTGTGGGTAAGG	
rkp 021	rkp-021-A	GGGGTGCCATCGGAGTATAG	Amplification of 5′ flanking region
	rkp-021-C	CTGTGGACTCGTATGCTGTTGCGCAGCCAGGGAGAAGTCCTATGCTT	
	rkp-021-B	CTGACCTATCTTCCTGGACATGACGGGGTTAGACAGCTAGCTTTCCAC	Amplification of 3′ flanking region
	rkp-021-D	GAGCCTCAAGGTCAACCAGA	
rkp 022	rkp-022-A	GCCCCGAAGAATCTCAGGTC	Amplification of 5′ flanking region
	rkp-022-C	CTGTGGACTCGTATGCTGTTGCGCAGCGTAGCGGAATGTGGCTGAC	
	rkp-022-B	CTGACCTATCTTCCTGGACATGACGGGAAAAGGGTGGGGTTGAGGG	Amplification of 3′ flanking region
	rkp-022-D	TTCGTCCGAGGCTACGAGAC	
rkp 024	rkp-024-A	GGCACCGTGTATTCGGCAAG	Amplification of 5′ flanking region
	rkp-024-C	CTGTGGACTCGTATGCTGTTGCGCAGCACCCTTGTTTTAACACGCA	
	rkp-024-B	CTGACCTATCTTCCTGGACATGACGGGAAGGGAGACCGGCAGTAGT	Amplification of 3′ flanking region
	rkp-024-D	TTGTGCAGATGTTACGGAGG	
rkp 025	rkp-025-A	ACCTAGCTGGCCCTTTGGGA	Amplification of 5′ flanking region
	rkp-025-C	CTGTGGACTCGTATGCTGTTGCGCAGGATCTCTGTTGCGGGCTACT	
	rkp-025-B	CTGACCTATCTTCCTGGACATGACGGTCACCCTGGAGCTGAGGTGG	Amplification of 3′ flanking region
	rkp-025-D	GGTTTGAAGCCGGTCACTCT	
rkp 027	rkp-027-A	TGGCGGTTTTGGGGATGTTC	Amplification of 5′ flanking region
	rkp-027-C	CTGTGGACTCGTATGCTGTTGCGCAGCGCGTGCTTGTGTTTCAGGG	
	rkp-027-B	CTGACCTATCTTCCTGGACATGACGGAGGGCAAAACGAAACCCACT	Amplification of 3′ flanking region
	rkp-027-D	GGTTACGGTCCGAAGGGACA	
rkp 029	rkp-029-A	TGGGAAGTAACCAGCGGACT	Amplification of 5′ flanking region
	rkp-029-C	CTGTGGACTCGTATGCTGTTGCGCAGTGCATTCACACCCGATCACT	
	rkp-029-B	CTGACCTATCTTCCTGGACATGACGGACCGGTAGGAGGGGTTCTTG	Amplification of 3′ flanking region
	rkp-029-D	TTCAGTGCGAGGAGCTTCTG	
rkp 031	rkp-031-A	GATTGGGGAAGGCGAATGGG	Amplification of 5′ flanking region
	rkp-031-C	CTGTGGACTCGTATGCTGTTGCGCAGGTTCTTGGAGCGCCACTTCC	
	rkp-031-B	CTGACCTATCTTCCTGGACATGACGGGAACTGCCGTTGGTGACGAG	Amplification of 3′ flanking region
	rkp-031-D	GGCGCTTCTGGTAGGGATCT	
rkp 032	rkp-032-A	GAGGTCACCCTTTGCAGCAG	Amplification of 5′ flanking region
	rkp-032-C	CTGTGGACTCGTATGCTGTTGCGCAGGCCCTACACTTGGCTGTTCC	
	rkp-032-B	CTGACCTATCTTCCTGGACATGACGGCCAAAGGTCCGCATCACCAG	Amplification of 3′ flanking region
	rkp-032-D	GTGCGACCTTTCCAGCATGT	
rkp 033	rkp-033-A	TGATGAACGGGAACCCGAGA	Amplification of 5′ flanking region
	rkp-033-C	CTGTGGACTCGTATGCTGTTGCGCAGGGCTTGGCTATTCAGACTGG	
	rkp-033-B	CTGACCTATCTTCCTGGACATGACGGGGAGATGTCGGGGCTGAAGA	Amplification of 3′ flanking region
	rkp-033-D	ATTGCGGAAGTGCATTACGG	
rkp 036	rkp-036-A	GCAAACAGTGAAACCACTAC	Amplification of 5′ flanking region
	rkp-036-C	CTGTGGACTCGTATGCTGTTGCGCAGGTAGTACTACACCACAATCC	
	rkp-036-B	CTGACCTATCTTCCTGGACATGACGGTAGTAGGGAAAGGGTGTGAG	Amplification of 3′ flanking region
	rkp-036-D	CACGCGATTGATGTGACAAC	
rkp 040	rkp-040-A	CTACCGTCCAGTTGCTACAG	Amplification of 5′ flanking region
	rkp-040-C	CTGTGGACTCGTATGCTGTTGCGCAGTGAGTCTAGGCAGCCACGAA	
	rkp-040-B	CTGACCTATCTTCCTGGACATGACGGACAATCGATAAGGACCCTTT	Amplification of 3′ flanking region
	rkp-040-D	CCTCCGGTAGCATCGCTAGA	
rkp 041	rkp-041-A	ATGTAGGAGAGCCAGGCGATAA	Amplification of 5′ flanking region
	rkp-041-C	CTGTGGACTCGTATGCTGTTGCGCAGAGAGGCCATTGCTCAAGTCA	
	rkp-041-B	CTGACCTATCTTCCTGGACATGACGGGGGAATGCTTGTTCGAGTTGGA	Amplification of 3′ flanking region
	rkp-041-D	AAACCCTCAAAGCCCGGAAA	
rkp 043	rkp-043-A	AAGAACTACGCGGTGGTAGA	Amplification of 5′ flanking region
	rkp-043-C	CTGTGGACTCGTATGCTGTTGCGCAGGCAGTCTGGGTTTGTCGCTC	
	rkp-043-B	CTGACCTATCTTCCTGGACATGACGGCAACCAGGGCTTAATGGTGG	Amplification of 3′ flanking region
	rkp-043-D	GAACGAACACCCACTCAGCC	
rkp 045	rkp-045-A	GCGGTGCTTGAAAAGTCCCT	Amplification of 5′ flanking region
	rkp-045-C	CTGTGGACTCGTATGCTGTTGCGCAGGTTCCGGAGTTCGATGCGTG	
	rkp-045-B	CTGACCTATCTTCCTGGACATGACGGTTTGCCGCACAAGCTAACGA	Amplification of 3′ flanking region
	rkp-045-D	CCCCGGAAAGACATGAGCTG	
rkp 046	rkp-046-A	GGCACAAGATTCCTTTGCGT	Amplification of 5′ flanking region
	rkp-046-C	CTGTGGACTCGTATGCTGTTGCGCAGAATCCCTTCCTCTTTGTACC	
	rkp-046-B	CTGACCTATCTTCCTGGACATGACGGGGCGACTATTGAAGTATCCC	Amplification of 3′ flanking region
	rkp-046-D	GCGTGAACAACAGCATCGAG	
rkp 047	rkp-047-A	TAGCCGCAGCTCTTAGCGCA	Amplification of 5′ flanking region
	rkp-047-C	CTGTGGACTCGTATGCTGTTGCGCAGTGCACGTCCTCACTACCCAA	
	rkp-047-B	CTGACCTATCTTCCTGGACATGACGGAGCAAGGACGGGTGCAGAGG	Amplification of 3′ flanking region
	rkp-047-D	CAATCGCTCTGCCGTGCAAG	
rkp 057	rkp-057-A	TCAGTACAGAGCTGGCACTTTG	Amplification of 5′ flanking region
	rkp-057-C	CTGTGGACTCGTATGCTGTTGCGCAGCGGCTATACTTCCATTTCATGCTCT	
	rkp-057-B	CTGACCTATCTTCCTGGACATGACGGAGGTCAGACGACCCATCCTC	Amplification of 3′ flanking region
	rkp-057-D	AGGTGAAGTCTCCCGCTCTT	
rkp 059	rkp-059-A	ACAAGCGATGCACAATGCTAC	Amplification of 5′ flanking region
	rkp-059-C	CTGTGGACTCGTATGCTGTTGCGCAGGGTCACGATTGCTGTGGGTT	
	rkp-059-B	CTGACCTATCTTCCTGGACATGACGGCTCATTGCCGAGACTGGTGA	Amplification of 3′ flanking region
	rkp-059-D	CGTGGTGCTGGATACACCTT	
rkp 060	rkp-060-A	TGAATCGCCGTGAACCATCC	Amplification of 5′ flanking region
	rkp-060-C	CTGTGGACTCGTATGCTGTTGCGCAGAGGTCCGTTTCGCATTGGTC	
	rkp-060-B	CTGACCTATCTTCCTGGACATGACGGGTATCGCAGGCCGGGAAAAC	Amplification of 3′ flanking region
	rkp-060-D	GTCGAAGCACCCACAATCCC	
rkp 062	rkp-062-A	ACATGCTGTCGCCTTGGATG	Amplification of 5′ flanking region
	rkp-062-C	CTGTGGACTCGTATGCTGTTGCGCAGCCCTTCAGTCCGAGTCAGCA	
	rkp-062-B	CTGACCTATCTTCCTGGACATGACGGAGCCGGTTCTTGAAAGCCTG	Amplification of 3′ flanking region
	rkp-062-D	ACCTTCCCCGGTCGCATTAC	
rkp 064	rkp-064-A	CGGTCGGACGTTGATTTACCT	Amplification of 5′ flanking region
	rkp-064-C	CTGTGGACTCGTATGCTGTTGCGCAGGTGGGCCTGTCCTTTCATCA	
	rkp-064-B	CTGACCTATCTTCCTGGACATGACGGCTTCCAGTGAGGCCCAAGTC	Amplification of 3′ flanking region
	rkp-064-D	TTCGTTCAGCTTGCTCACCT	
rkp 066	rkp-066-A	GGCCAAGTTCATCGCTAGCA	Amplification of 5′ flanking region
	rkp-066-C	CTGTGGACTCGTATGCTGTTGCGCAGTAACCGGGAAGCATGCAACA	
	rkp-066-B	CTGACCTATCTTCCTGGACATGACGGCTTGTCCAGCGTCGTAGAGG	Amplification of 3′ flanking region
	rkp-066-D	ATTGCACACAGACGACGTCA	
rkp 067	rkp-067-A	TGCTCTTGACAGATCCGCGT	Amplification of 5′ flanking region
	rkp-067-C	CTGTGGACTCGTATGCTGTTGCGCAGCCTGAGGGTCCGGTGCTATG	
	rkp-067-B	CTGACCTATCTTCCTGGACATGACGGTGAAGTGGGAGGATGCGGAG	Amplification of 3′ flanking region
	rkp-067-D	TAGTCACTCCCCGCTCCAAG	
rkp 068	rkp-068-A	CGGAGGGGAAGCTTGGGTAG	Amplification of 5′ flanking region
	rkp-068-C	CTGTGGACTCGTATGCTGTTGCGCAGATCTAATGCCGTCCCAAGGT	
	rkp-068-B	CTGACCTATCTTCCTGGACATGACGGACCAAGCACGAGTCTCGAGG	Amplification of 3′ flanking region
	rkp-068-D	ACCGAAATCCCACCCATCTC	
rkp 073	rkp-073-A	TACCCCACGGTTGGATAGGG	Amplification of 5′ flanking region
	rkp-073-C	CTGTGGACTCGTATGCTGTTGCGCAGTCACAAAAGGCACGGATGTGA	
	rkp-073-B	CTGACCTATCTTCCTGGACATGACGGTGTGCTTATCGCATTTGAGTGATGT	Amplification of 3′ flanking region
	rkp-073-D	TACCCCACGGTTGGATAGGG	
rkp 076	rkp-076-A	GTCCCGATTGGTCGTGGTAG	Amplification of 5′ flanking region
	rkp-076-C	CTGTGGACTCGTATGCTGTTGCGCAGTGCCTGTTTAAGCGCGATTG	
	rkp-076-B	CTGACCTATCTTCCTGGACATGACGGCCCCGGGCTTGATAAGACAC	Amplification of 3′ flanking region
	rkp-076-D	TGGACCGATGGTTGTAACCC	
rkp 079	rkp-079-A	TCTAAGGACTTCTCCAGTGT	Amplification of 5′ flanking region
	rkp-079-C	CTGTGGACTCGTATGCTGTTGCGCAGCATTGCTGATTGGATGGTGT	
	rkp-079-B	CTGACCTATCTTCCTGGACATGACGGGGGGGTTGATATTAGCCGTTT	Amplification of 3′ flanking region
	rkp-079-D	GCTACCAGAGTAGGTGGCAA	
rkp 081	rkp-081-A	CCCCTTTCGCGTGTTCTTCC	Amplification of 5′ flanking region
	rkp-081-C	CTGTGGACTCGTATGCTGTTGCGCAGACGCGTTTGCTTGACCCAGA	
	rkp-081-B	CTGACCTATCTTCCTGGACATGACGGCACGCGAACGGTCGTAACAC	Amplification of 3′ flanking region
	rkp-081-D	GAGTGGCTTACGGCTGCGAA	
rkp 082	rkp-082-A	TCGAGTACCTCGCTCCTACT	Amplification of 5′ flanking region
	rkp-082-C	CTGTGGACTCGTATGCTGTTGCGCAGCCTACAAGGCCGACCACTAC	
	rkp-082-B	CTGACCTATCTTCCTGGACATGACGGTTCACCGAGGTGGTAATCCC	Amplification of 3′ flanking region
	rkp-082-D	TCACTGGGTTTCCAACGCTT	
rkp 085	rkp-085-A	GCATTGTTGAGACCTCTGTATGAC	Amplification of 5′ flanking region
	rkp-085-C	CTGTGGACTCGTATGCTGTTGCGCAGTCTTCAACGACCTGCATCAAAC	
	rkp-085-B	CTGACCTATCTTCCTGGACATGACGGAGAAAGCGGGGTGACGTATG	Amplification of 3′ flanking region
	rkp-085-D	AGTTCCTTGCCTTCACCCTT	
rkp 086	rkp-086-A	GAAGCCGGTGTGTTACCTTTG	Amplification of 5′ flanking region
	rkp-086-C	CTGTGGACTCGTATGCTGTTGCGCAGAGTCATTTCCTCTGCCGTCAA	
	rkp-086-B	CTGACCTATCTTCCTGGACATGACGGTGATGACTGTCGGATAATACAGGA	Amplification of 3′ flanking region
	rkp-086-D	CTAACGAACCCCAGCTTACGA	
rkp 088	rkp-088-A	ACGAGTGGACCCGGGAAAAG	Amplification of 5′ flanking region
	rkp-088-C	CTGTGGACTCGTATGCTGTTGCGCAGTGCATGGGATACGGCGAACA	
	rkp-088-B	CTGACCTATCTTCCTGGACATGACGGCCTGTGAAGTGGGAGGGTGA	Amplification of 3′ flanking region
	rkp-088-D	CGCCGAGGGATCCAAACGAC	
rkp 089	rkp-089-A	CAAAGGGGTGCTCGTCCTTG	Amplification of 5′ flanking region
	rkp-089-C	CTGTGGACTCGTATGCTGTTGCGCAGAAAGCCAGACGCTTGACCAG	
	rkp-089-B	CTGACCTATCTTCCTGGACATGACGGTTGGCACATGTCCCAGTTCC	Amplification of 3′ flanking region
	rkp-089-D	AGCTGCGGACAATGCTTGAC	
rkp 092	rkp-092-A	TGCCAGCTTCATCCGAGTAA	Amplification of 5′ flanking region
	rkp-092-C	CTGTGGACTCGTATGCTGTTGCGCAGTGTACCTCTGCCACACGTAG	
	rkp-092-B	CTGACCTATCTTCCTGGACATGACGGGGATCGCCGCCTATCTACAG	Amplification of 3′ flanking region
	rkp-092-D	GGGCATTTTCAACGGCGTAA	
rkp 094	rkp-094-A	ATCGTCCGCGGTTACATCAC	Amplification of 5′ flanking region
	rkp-094-C	CTGTGGACTCGTATGCTGTTGCGCAGCACCTAGATCTGCCCTACACC	
	rkp-094-B	CTGACCTATCTTCCTGGACATGACGGACTGAAGCAGACGGAGAGGA	Amplification of 3′ flanking region
	rkp-094-D	AGGACCCATAGCACTACCGA	
rkp 095	rkp-095-A	GCCCTGCAGTAGTTTAAAGCC	Amplification of 5′ flanking region
	rkp-095-C	CTGTGGACTCGTATGCTGTTGCGCAGTTCCTTGTCGAATTTGCATGTTCT	
	rkp-095-B	CTGACCTATCTTCCTGGACATGACGGTGCCTTAGGACCGACCTATG	Amplification of 3′ flanking region
	rkp-095-D	TGACGCCAATCATGAAGCCA	
rkp 096	rkp-096-A	GGGAGAAGCACAAAGTCACTCT	Amplification of 5′ flanking region
	rkp-096-C	CTGTGGACTCGTATGCTGTTGCGCAGTCAGCTCTGGTCATAGATACTCACT	
	rkp-096-B	CTGACCTATCTTCCTGGACATGACGGACTACTTTGGGGATCGTGCATC	Amplification of 3′ flanking region
	rkp-096-D	CGGCAGCGAAAACATCCAAC	
rkp 101	rkp-101-A	TCCATAGGCAATCCCGATAC	Amplification of 5′ flanking region
	rkp-101-C	CTGTGGACTCGTATGCTGTTGCGCAGCTCACACGTACAGCAAACCC	
	rkp-101-B	CTGACCTATCTTCCTGGACATGACGGAGCACAAGCCTAGACGAACT	Amplification of 3′ flanking region
	rkp-101-D	ACTATTCGCGTCCAACGAAC	
rkp 106	rkp-106-A	ACCGTTTGGTTAATGACAGACCT	Amplification of 5′ flanking region
	rkp-106-C	CTGTGGACTCGTATGCTGTTGCGCAGAAAGCCCTGTAGATTGGTTGAGA	
	rkp-106-B	CTGACCTATCTTCCTGGACATGACGGTGGACGTCCACAGTCCGTAA	Amplification of 3′ flanking region
	rkp-106-D	CACGGTATGCTCTCCCTGTTT	
rkp 107	rkp-107-A	TTCCGTTGGTGGGAGTTTGG	Amplification of 5′ flanking region
	rkp-107-C	CTGTGGACTCGTATGCTGTTGCGCAGCCTCGCAAACATGTCACTAC	
	rkp-107-B	CTGACCTATCTTCCTGGACATGACGGCGGTTTACTTTCCCCACGTT	Amplification of 3′ flanking region
	rkp-107-D	TATCCGGGGTCAACTGCAGA	
rkp 112	rkp-112-A	CCTGCCTCTTTGACCGTAACT	Amplification of 5′ flanking region
	rkp-112-C	CTGTGGACTCGTATGCTGTTGCGCAGTGATTACGAGCGTAGAAACTCCTTC	
	rkp-112-B	CTGACCTATCTTCCTGGACATGACGGTGTCGATGGGAAATAGAAGGACA	Amplification of 3′ flanking region
	rkp-112-D	CCTCTTTCCACGTCGCATCA	
rkp 115	rkp-115-A	AGCAAGGGCCAACACTAAGG	Amplification of 5′ flanking region
	rkp-115-C	CTGTGGACTCGTATGCTGTTGCGCAGCTCGTCGCAACCCAAACCTG	
	rkp-115-B	CTGACCTATCTTCCTGGACATGACGGGAAGATGCGCCCAATTCGGT	Amplification of 3′ flanking region
	rkp-115-D	TCGCTGACGCGGAATAGCTG	
rkp 117	rkp-117-A	GGGCACCGATATTTCCGCTC	Amplification of 5′ flanking region
	rkp-117-C	CTGTGGACTCGTATGCTGTTGCGCAGTCCAGCAGTCGTACACTCCA	
	rkp-117-B	CTGACCTATCTTCCTGGACATGACGGACTCCGTACCCCGCATAGAC	Amplification of 3′ flanking region
	rkp-117-D	CTTGCGCCTGCTTCGTACTC	
rkp 118	rkp-118-A	ACCAAGAGTCGGCACCTATC	Amplification of 5′ flanking region
	rkp-118-C	CTGTGGACTCGTATGCTGTTGCGCAGCACGCGTAGTTGCATTACCC	
	rkp-118-B	CTGACCTATCTTCCTGGACATGACGGCCTAGAAAGGAAGCCTTCGT	Amplification of 3′ flanking region
	rkp-118-D	GTGTCATCTTCCCCGGAACT	
rkp 120	rkp-120-A	CACAACGGTAAAGGCGCAAA	Amplification of 5′ flanking region
	rkp-120-C	CTGTGGACTCGTATGCTGTTGCGCAGCTCGATGTGCTGGTCACGTT	
	rkp-120-B	CTGACCTATCTTCCTGGACATGACGGGGTCCTTCACAGCGAGTACC	Amplification of 3′ flanking region
	rkp-120-D	TGTAACGCCGGCTTGCATTC	
rkp 121	rkp-121-A	ATCAGTCAATGGTGGGAACTGA	Amplification of 5′ flanking region
	rkp-121-C	CTGTGGACTCGTATGCTGTTGCGCAGCCAGCAGGACAAACTAGCCA	
	rkp-121-B	CTGACCTATCTTCCTGGACATGACGGCAGGTCCAAGACGAACACCT	Amplification of 3′ flanking region
	rkp-121-D	GCTGCCTCACCTACATCGAC	
rkp 125	rkp-125-A	GGGATCAACAGCGAAGTCAG	Amplification of 5′ flanking region
	rkp-125-C	CTGTGGACTCGTATGCTGTTGCGCAGGAAACGAAGAAATGTAACGG	
	rkp-125-B	CTGACCTATCTTCCTGGACATGACGGGAACCTCTCCTGTCCAGTCA	Amplification of 3′ flanking region
	rkp-125-D	GAACAAGGACGAACCACTCA	
rkp 130	rkp-130-A	GGCAGTAATCGGCATAGCTC	Amplification of 5′ flanking region
	rkp-130-C	CTGTGGACTCGTATGCTGTTGCGCAGGCAACTCAACCGCTTCACAG	
	rkp-130-B	CTGACCTATCTTCCTGGACATGACGGGTAGCGGACGAAGAAAGGTG	Amplification of 3′ flanking region
	rkp-130-D	GCTTGGCAATCTCCGCATTC	
rkp 132	rkp-132-A	AAGGAGGAAAGGCCGGATGA	Amplification of 5′ flanking region
	rkp-132-C	CTGTGGACTCGTATGCTGTTGCGCAGTGCAAATCCCAACAGCCACA	
	rkp-132-B	CTGACCTATCTTCCTGGACATGACGGGCGTTGTGACCGGTGAGAAG	Amplification of 3′ flanking region
	rkp-132-D	CATAGGTCACGCTTGCCGTC	
rkp 133	rkp-133-A	AGAAGTTGCAGGTGGGAATG	Amplification of 5′ flanking region
	rkp-133-C	CTGTGGACTCGTATGCTGTTGCGCAGAGTGTATTCTGTGTGCGTAA	
	rkp-133-B	CTGACCTATCTTCCTGGACATGACGGTCACCTGATAATCCCTAGGT	Amplification of 3′ flanking region
	rkp-133-D	GTTCCACCGCTACTCCCTAA	
rkp 134	rkp-134-A	ACGGTGCAAACTCACCACTT	Amplification of 5′ flanking region
	rkp-134-C	CTGTGGACTCGTATGCTGTTGCGCAGGCCATATAACGCCCTCGCAA	
	rkp-134-B	CTGACCTATCTTCCTGGACATGACGGCCGTGCTTCTAGCTAGTCCT	Amplification of 3′ flanking region
	rkp-134-D	TAGCCCGACAAAGCACGAAC	
rkp 135	rkp-135-A	CCTTGCACCCACCGATGAAG	Amplification of 5′ flanking region
	rkp-135-C	CTGTGGACTCGTATGCTGTTGCGCAGGGCTCATCGTCACCCCATGA	
	rkp-135-B	CTGACCTATCTTCCTGGACATGACGGCCCACGCCAAAGACGTTACC	Amplification of 3′ flanking region
	rkp-135-D	AGCTTATCCGCGCCCATAGT	
rkp 140	rkp-140-A	TTAGAGATCCTAGGAGCACA	Amplification of 5′ flanking region
	rkp-140-C	CTGTGGACTCGTATGCTGTTGCGCAGAGCGTGAATTAGATTCCTTACACT	
	rkp-140-B	CTGACCTATCTTCCTGGACATGACGGGAGCATATGCTCCGCGTAAG	Amplification of 3′ flanking region
	rkp-140-D	GCACCAGTTCCGATGCTTAC	
rkp 142	rkp-142-A	ATGCAGCGAGAGAACACTCA	Amplification of 5′ flanking region
	rkp-142-C	CTGTGGACTCGTATGCTGTTGCGCAGTGCTTCTCCGACTTGTCTAC	
	rkp-142-B	CTGACCTATCTTCCTGGACATGACGGCAAGCGTTACTGGAGTAAGG	Amplification of 3′ flanking region
	rkp-142-D	TCCACGACAGAGCACCTACA	
rkp 144	rkp-144-A	AGATCTACGGCGAGCAGTGT	Amplification of 5′ flanking region
	rkp-144-C	CTGTGGACTCGTATGCTGTTGCGCAGATCAGGGATTGGCGACGAGA	
	rkp-144-B	CTGACCTATCTTCCTGGACATGACGGGTGTGCGGTTCCTTAGGCAG	Amplification of 3′ flanking region
	rkp-144-D	GGTGCATCTTCGGATGGCAG	
rkp 145	rkp-145-A	CCTCATCAATTCCAACGACT	Amplification of 5′ flanking region
	rkp-145-C	CTGTGGACTCGTATGCTGTTGCGCAGATCTCGGGAGTCACTTCGAC	
	rkp-145-B	CTGACCTATCTTCCTGGACATGACGGACTCCTGGTTCTCGACACTC	Amplification of 3′ flanking region
	rkp-145-D	GCTTTCTCATCTGGCACACT	
rkp 147	rkp-147-A	CTGTTGCAGTCTAATTGCGA	Amplification of 5′ flanking region
	rkp-147-C	CTGTGGACTCGTATGCTGTTGCGCAGTACTCGCCTGTAGTTGCAGA	
	rkp-147-B	CTGACCTATCTTCCTGGACATGACGGAACCACTGGGGTAGTGAGAG	Amplification of 3′ flanking region
	rkp-147-D	CACAAGCCGATTGCTAACCC	
rkp 148	rkp-148-A	AGCGCTTTGCTCTATGCTGG	Amplification of 5′ flanking region
	rkp-148-C	CTGTGGACTCGTATGCTGTTGCGCAGTGGTCATCGAGCCTCCATGT	
	rkp-148-B	CTGACCTATCTTCCTGGACATGACGGTCTCACTCGTGCAGCTGTGT	Amplification of 3′ flanking region
	rkp-148-D	CAGCTCGACCATCGGGTTTC	
rkp 149	rkp-149-A	CAAGCGCAACTCGGACCTTG	Amplification of 5′ flanking region
	rkp-149-C	CTGTGGACTCGTATGCTGTTGCGCAGCAGATACCGATCCCCGTAGA	
	rkp-149-B	CTGACCTATCTTCCTGGACATGACGGGGGTGGAGGATGCGGAATTG	Amplification of 3′ flanking region
	rkp-149-D	AAGTTTTAGGCGGCTTTGTG	
rkp 150	rkp-150-A	GCTTTCGTTATCGCCGAGGTT	Amplification of 5′ flanking region
	rkp-150-C	CTGTGGACTCGTATGCTGTTGCGCAGGGGAAGCGCTGGAGCTATTG	
	rkp-150-B	CTGACCTATCTTCCTGGACATGACGGGGATTCGGCTGTGGTAGGTTG	Amplification of 3′ flanking region
	rkp-150-D	TGCCTCCGAAGAGTCACAGA	
rkp 151	rkp-151-A	GGCATCAAGTCCCGCACATT	Amplification of 5′ flanking region
	rkp-151-C	CTGTGGACTCGTATGCTGTTGCGCAGGGCTTGAGCAATCGCGCATT	
	rkp-151-B	CTGACCTATCTTCCTGGACATGACGGCGCAGCCAGTTTGACTGTGA	Amplification of 3′ flanking region
	rkp-151-D	ACTCCCCACGCTCTTATCGG	
rkp 152	rkp-152-A	TCTGGAGAGGACTGGGGTAA	Amplification of 5′ flanking region
	rkp-152-C	CTGTGGACTCGTATGCTGTTGCGCAGATTCCGATGTTGGATCTGTAATACC	
	rkp-152-B	CTGACCTATCTTCCTGGACATGACGGCCATTGAGGGAGGTTCGTGT	Amplification of 3′ flanking region
	rkp-152-D	CGCCATCGAATGCAACGTAG	
rkp 153	rkp-153-A	TTCCAGAGGTTGCTTCCCAG	Amplification of 5′ flanking region
	rkp-153-C	CTGTGGACTCGTATGCTGTTGCGCAGGGTTTGTCACCGGGCCATAG	
	rkp-153-B	CTGACCTATCTTCCTGGACATGACGGTTCTTCAACCCCACCTTCGT	Amplification of 3′ flanking region
	rkp-153-D	AGCACCAACGCTCCAAACAG	
rkp 158	rkp-158-A	CCTCAGATTGCCTGGCGATTT	Amplification of 5′ flanking region
	rkp-158-C	CTGTGGACTCGTATGCTGTTGCGCAGTTCTGCGGTTGGGTGACAAA	
	rkp-158-B	CTGACCTATCTTCCTGGACATGACGGTGAGCCGCTAGTTTGGCTTT	Amplification of 3′ flanking region
	rkp-158-D	TTCTGCGGTTGGGTGACAAA	
rkp 161	rkp-161-A	CAGGGTCTAGTGGTCCACAA	Amplification of 5′ flanking region
	rkp-161-C	CTGTGGACTCGTATGCTGTTGCGCAGCACACATAGGGCGGGGACTT	
	rkp-161-B	CTGACCTATCTTCCTGGACATGACGGGCGGTACCCTAAAATAGCCC	Amplification of 3′ flanking region
	rkp-161-D	GGCACGCGAAAACAGTACGA	
rkp 162	rkp-162-A	ATATCGACGTCAACCAGCTT	Amplification of 5′ flanking region
	rkp-162-C	CTGTGGACTCGTATGCTGTTGCGCAGGAGTTTGGGGCCTCCTACAC	
	rkp-162-B	CTGACCTATCTTCCTGGACATGACGGTGTCCTCTCGCAGGAGCTAC	Amplification of 3′ flanking region
	rkp-162-D	ATATGCTCCGAGCCAGGACA	
rkp 163	rkp-163-A	TAGATGGGACGCCCTGTTTC	Amplification of 5′ flanking region
	rkp-163-C	CTGTGGACTCGTATGCTGTTGCGCAGTGAGATGATTGACGCAAATAACCAC	
	rkp-163-B	CTGACCTATCTTCCTGGACATGACGGATCCAGGATGGCAGTGATGT	Amplification of 3′ flanking region
	rkp-163-D	CGGCATCCATGACTCTCACT	
rkp 164	rkp-164-A	CCCCATGTCGTGCTTCTCTG	Amplification of 5′ flanking region
	rkp-164-C	CTGTGGACTCGTATGCTGTTGCGCAGAAACAGTCGCACCTGTCTAC	
	rkp-164-B	CTGACCTATCTTCCTGGACATGACGGATAAGCCGAGATGTTGCACT	Amplification of 3′ flanking region
	rkp-164-D	GAGAGAACACGACCGCCAAA	
rkp 165	rkp-165-A	GGCAACCAACAGGGAATCGG	Amplification of 5′ flanking region
	rkp-165-C	CTGTGGACTCGTATGCTGTTGCGCAGATCAATTGACCTGTCGCAAA	
	rkp-165-B	CTGACCTATCTTCCTGGACATGACGGGATCCGGCACTGTGGATAGG	Amplification of 3′ flanking region
	rkp-165-D	GAAGTTGCGGTGTACGTACT	
rkp 167	rkp-167-A	GCGTACCCTCCGCAGTACAG	Amplification of 5′ flanking region
	rkp-167-C	CTGTGGACTCGTATGCTGTTGCGCAGTAGACCATGTCACAATGCCT	
	rkp-167-B	CTGACCTATCTTCCTGGACATGACGGCGACGAGAAGGACCTTCGCA	Amplification of 3′ flanking region
	rkp-167-D	TGTTTCTACGGTCAGATCCA	
rkp 168	rkp-168-A	TTAGCACGTGCTTCTAGCGA	Amplification of 5′ flanking region
	rkp-168-C	CTGTGGACTCGTATGCTGTTGCGCAGGGAATCGTGTCGTTCCGGTG	
	rkp-168-B	CTGACCTATCTTCCTGGACATGACGGCAAAGCAGGGAAAGAGGGGT	Amplification of 3′ flanking region
	rkp-168-D	TAACTAGGTGCCCTTGCGCT	
rkp 171	rkp-171-A	GGATGACAAGAGGGGTGTGA	Amplification of 5′ flanking region
	rkp-171-C	CTGTGGACTCGTATGCTGTTGCGCAGCGTTGTCACCCAACCTTGTG	
	rkp-171-B	CTGACCTATCTTCCTGGACATGACGGCTAGCCAGGGGTAGAACGTG	Amplification of 3′ flanking region
	rkp-171-D	TAGGTTTCCCTCCTTGGCTT	
rkp 175	rkp-175-A	CCGCCGACTCATTAACCGTG	Amplification of 5′ flanking region
	rkp-175-C	CTGTGGACTCGTATGCTGTTGCGCAGGAGCGTTTGGGAGGGGTTTT	
	rkp-175-B	CTGACCTATCTTCCTGGACATGACGGCTGTCGCCCAACCATCATCC	Amplification of 3′ flanking region
	rkp-175-D	GTCTGCTGGGGAGCGATAGA	
rkp 176	rkp-176-A	AGATGTCAACGCGATCGCAA	Amplification of 5′ flanking region
	rkp-176-C	CTGTGGACTCGTATGCTGTTGCGCAGAGGAAGCCAATTTGTGAGAG	
	rkp-176-B	CTGACCTATCTTCCTGGACATGACGGTCATTGCATGCCACTCGAAC	Amplification of 3′ flanking region
	rkp-176-D	AGGCCCGCTAAATAGCTAGA	
rkp 177	rkp-177-A	CCACAGCAAAACGCACGTCA	Amplification of 5′ flanking region
	rkp-177-C	CTGTGGACTCGTATGCTGTTGCGCAGTTGGTCTTATTCTCTGAGCC	
	rkp-177-B	CTGACCTATCTTCCTGGACATGACGGCGGTCAAGGAGGGGCAGAAC	Amplification of 3′ flanking region
	rkp-177-D	AACACGTAATCCCAATCTCC	
rkp 178	rkp-178-A	ACGAGGCTCTGTTCCGCTTC	Amplification of 5′ flanking region
	rkp-178-C	CTGTGGACTCGTATGCTGTTGCGCAGATGCGGTTCCTGGAGGACTG	
	rkp-178-B	CTGACCTATCTTCCTGGACATGACGGCCAGTTCTTCCCTCGCTGGT	Amplification of 3′ flanking region
	rkp-178-D	TCCTGGACGCTAATGTCGGG	
rkp 181	rkp-181-A	CGAGGATGGGGTGATAACGG	Amplification of 5′ flanking region
	rkp-181-C	CTGTGGACTCGTATGCTGTTGCGCAGACAAGGCGGTCGATCTTAGG	
	rkp-181-B	CTGACCTATCTTCCTGGACATGACGGACCGAAAGAGCAAACTGTACAC	Amplification of 3′ flanking region
	rkp-181-D	CACACAGGAACTGGACCACA	
rkp 184	rkp-184-A	CATTACTCACGCCCCAGAAG	Amplification of 5′ flanking region
	rkp-184-C	CTGTGGACTCGTATGCTGTTGCGCAGCATTGATGCCATGTCCACTT	
	rkp-184-B	CTGACCTATCTTCCTGGACATGACGGCAGGAATTCGTCAGAGGCAA	Amplification of 3′ flanking region
	rkp-184-D	TCTACTCCGGAGGAACTAGG	
rkp 185	rkp-185-A	CGCCCATGGTGTATACCGAC	Amplification of 5′ flanking region
	rkp-185-C	CTGTGGACTCGTATGCTGTTGCGCAGCAGCTTGACACGCTAGGCAG	
	rkp-185-B	CTGACCTATCTTCCTGGACATGACGGGTAAGCACCGCCTACAGCAG	Amplification of 3′ flanking region
	rkp-185-D	TCCTTCCACCGTCATACCCA	
rkp 188	rkp-188-A	TGTCGAACGGAGAGTGACGG	Amplification of 5′ flanking region
	rkp-188-C	CTGTGGACTCGTATGCTGTTGCGCAGTCTACGAGCCCAAGCACTCT	
	rkp-188-B	CTGACCTATCTTCCTGGACATGACGGGGAAAGCAGAGGTGCTGGTG	Amplification of 3′ flanking region
	rkp-188-D	GTCGCATGCCATCAGCGTAA	
rkp 194	rkp-194-A	AAGGATGATGGGCGACGTGA	Amplification of 5′ flanking region
	rkp-194-C	CTGTGGACTCGTATGCTGTTGCGCAGGACAGGAGCTCTCCGCTAGT	
	rkp-194-B	CTGACCTATCTTCCTGGACATGACGGCTTTGGCACGGGTTGAGGTG	Amplification of 3′ flanking region
	rkp-194-D	ACACCAAGGTACGGTCCAGT	
rkp 195	rkp-195-A	CTCTCAGGCTCCGAGCAGAA	Amplification of 5′ flanking region
	rkp-195-C	CTGTGGACTCGTATGCTGTTGCGCAGAAAACCCGGATCTCTCCCGT	
	rkp-195-B	CTGACCTATCTTCCTGGACATGACGGACTTCCTTGGTCAACTGCGG	Amplification of 3′ flanking region
	rkp-195-D	AGCAGTCTGTCGACAGACCA	
rkp 199	rkp-199-A	CGTCTCAGCCAAGCCCTTAC	Amplification of 5′ flanking region
	rkp-199-C	CTGTGGACTCGTATGCTGTTGCGCAGCCAACTCACCGACAGGACTT	
	rkp-199-B	CTGACCTATCTTCCTGGACATGACGGAATATGGGAGCCCCTGTCCA	Amplification of 3′ flanking region
	rkp-199-D	ACCGAGAGACCCGATAGCTC	
rkp 208	rkp-208-A	CATGGCCCTTCTATTCTTTGCTAC	Amplification of 5′ flanking region
	rkp-208-C	CTGTGGACTCGTATGCTGTTGCGCAGATGGTATGTGTGCCGGGAAA	
	rkp-208-B	CTGACCTATCTTCCTGGACATGACGGGTCGCGTGACGTAAAATGGG	Amplification of 3′ flanking region
	rkp-208-D	GCACGGACATCTGATCGACT	
rkp 212	rkp-212-A	GCTTCCTCCGTACAAGTGCAA	Amplification of 5′ flanking region
	rkp-212-C	CTGTGGACTCGTATGCTGTTGCGCAGCAACGCCGTTAGCGGTAACA	
	rkp-212-B	CTGACCTATCTTCCTGGACATGACGGGGCCCAGTGTTTCAAGTCTCT	Amplification of 3′ flanking region
	rkp-212-D	AGTGTGGTGTCGAGACACCT	
rkp 214	rkp-214-A	AGAGGTCCGATGCCGTAGAG	Amplification of 5′ flanking region
	rkp-214-C	CTGTGGACTCGTATGCTGTTGCGCAGTGTGGTCTTCCTAAGTGTGT	
	rkp-214-B	CTGACCTATCTTCCTGGACATGACGGAAGCTTGCCAAAAGATCCGT	Amplification of 3′ flanking region
	rkp-214-D	TTACGCCAGAAATCGAGCAA	
rkp 220	rkp-220-A	CTAGAGCCCTCCGAAAGGCA	Amplification of 5′ flanking region
	rkp-220-C	CTGTGGACTCGTATGCTGTTGCGCAGTTCGTTCACTGGTGCTTGCT	
	rkp-220-B	CTGACCTATCTTCCTGGACATGACGGGTCGGCGGTGAAGATCACAG	Amplification of 3′ flanking region
	rkp-220-D	TCGAGATCAAGGGCCACAAC	
rkp 222	rkp-222-A	CGAGCTGGAAGCTCTCAGAAC	Amplification of 5′ flanking region
	rkp-222-C	CTGTGGACTCGTATGCTGTTGCGCAGTTTGGCCTTGCATGATCGCT	
	rkp-222-B	CTGACCTATCTTCCTGGACATGACGGCCATCGTACAGCGTATCGCA	Amplification of 3′ flanking region
	rkp-222-D	AGCCTTCCTAGTCCACCCTT	
rkp 223	rkp-223-A	AGTGCAGCAACACCATAGTAACC	Amplification of 5′ flanking region
	rkp-223-C	CTGTGGACTCGTATGCTGTTGCGCAGTGTACTTTGCGGACGCGATT	
	rkp-223-B	CTGACCTATCTTCCTGGACATGACGGACTTGGTGAGTTTGGAGGTCTTTG	Amplification of 3′ flanking region
	rkp-223-D	TGACACCATCTCCTCACGTCA	
rkp 224	rkp-224-A	GCCAGGTGATAACCGACTCA	Amplification of 5′ flanking region
	rkp-224-C	CTGTGGACTCGTATGCTGTTGCGCAGACCTTTGAGAGCTACACCCC	
	rkp-224-B	CTGACCTATCTTCCTGGACATGACGGATATACGCAGCGTCAACGGG	Amplification of 3′ flanking region
	rkp-224-D	TTTGTACCGCAGGCGACTAC	
rkp 231	rkp-231-A	CCCTGCATAATTCCGTGGTT	Amplification of 5′ flanking region
	rkp-231-C	CTGTGGACTCGTATGCTGTTGCGCAGATACAGCGACCTGCCGTGTT	
	rkp-231-B	CTGACCTATCTTCCTGGACATGACGGCAATCTACCCAGCCCTGACT	Amplification of 3′ flanking region
	rkp-231-D	CAGTTTGGCACCAGACTCCT	
rkp 237	rkp-237-A	GTCTGCTCGTTCTGGCAGGT	Amplification of 5′ flanking region
	rkp-237-C	CTGTGGACTCGTATGCTGTTGCGCAGGCGACGTCTCAATTGCGTCA	
	rkp-237-B	CTGACCTATCTTCCTGGACATGACGGATTGATCACGGGTCTCCGCA	Amplification of 3′ flanking region
	rkp-237-D	TCGGATTGGCTCGTCCTCTT	
rkp 238	rkp-238-A	ACTCCGCTGGAAGAAGTCAG	Amplification of 5′ flanking region
	rkp-238-C	CTGTGGACTCGTATGCTGTTGCGCAGCTCCGTAAGACAGCCCTAGTG	
	rkp-238-B	CTGACCTATCTTCCTGGACATGACGGGACAATGGCAAGGAAGCAGT	Amplification of 3′ flanking region
	rkp-238-D	CTCACCTTCTTCGGGCGATT	
rkp 247	rkp-247-A	TCGGATTTCGGTGCCTGACT	Amplification of 5′ flanking region
	rkp-247-C	CTGTGGACTCGTATGCTGTTGCGCAGTAGTCGGATCCATGCCTCTG	
	rkp-247-B	CTGACCTATCTTCCTGGACATGACGGACCAAGACCACGACGCTACA	Amplification of 3′ flanking region
	rkp-247-D	ATTGCTGAGTTGGGTTGTGT	
rkp 249	rkp-249-A	CCCACTGGCTGTACCAAGAG	Amplification of 5′ flanking region
	rkp-249-C	CTGTGGACTCGTATGCTGTTGCGCAGTAGCTGGCAGCATCGTCTCT	
	rkp-249-B	CTGACCTATCTTCCTGGACATGACGGGACGATTGATGCAATGCGGG	Amplification of 3′ flanking region
	rkp-249-D	ATGCTCACAGCCTTCACCAC	
rkp 250	rkp-250-A	TGTCTGGAATCATGCACCTT	Amplification of 5′ flanking region
	rkp-250-C	CTGTGGACTCGTATGCTGTTGCGCAGATTGCAACCGCAAGTCACGA	
	rkp-250-B	CTGACCTATCTTCCTGGACATGACGGTCTCAACTACAGACAGGCCA	Amplification of 3′ flanking region
	rkp-250-D	GGGTGACGATGTCCTACCCA	
rkp 256	rkp-256-A	CGCTTGCCTTTGGGGAGTTT	Amplification of 5′ flanking region
	rkp-256-C	CTGTGGACTCGTATGCTGTTGCGCAGAGCAACGAGCCAGATAAGGG	
	rkp-256-B	CTGACCTATCTTCCTGGACATGACGGTGACGGTCAAGGACTGCAGA	Amplification of 3′ flanking region
	rkp-256-D	CAGACCACACAGCCAAGGTC	
rkp 272	rkp-272-A	CCGAGTGGATGCATATAGCACA	Amplification of 5′ flanking region
	rkp-272-C	CTGTGGACTCGTATGCTGTTGCGCAGGTTTGTAGAATACCGCGAACGAC	
	rkp-272-B	CTGACCTATCTTCCTGGACATGACGGTCTCAACGGCTCCTTTTGTTACT	Amplification of 3′ flanking region
	rkp-272-D	CTGTCACTTGAGCCAACTCCT	
rkp 273	rkp-273-A	CAATGTCGGATCGCAAAGTGT	Amplification of 5′ flanking region
	rkp-273-C	CTGTGGACTCGTATGCTGTTGCGCAGTACTGCCAGCTATCTGCACT	
	rkp-273-B	CTGACCTATCTTCCTGGACATGACGGAACAATGCACTGTGTCTTCCTG	Amplification of 3′ flanking region
	rkp-273-D	CACAGCAGGTTCGTGTTTCC	
rkp 274	rkp-274-A	ATGAAACCTCCCCGCTCTCT	Amplification of 5′ flanking region
	rkp-274-C	CTGTGGACTCGTATGCTGTTGCGCAGGGGTATTGCCGGCCTAGATG	
	rkp-274-B	CTGACCTATCTTCCTGGACATGACGGGGGCGGTTTTGGTGGTCATT	Amplification of 3′ flanking region
	rkp-274-D	ACTACACCAATACGTGGTATTCGG	
rkp 282	rkp-282-A	TTGGAGAAGGGAACTGGCAA	Amplification of 5′ flanking region
	rkp-282-C	CTGTGGACTCGTATGCTGTTGCGCAGGTGTTCCACAGATCGGCCAA	
	rkp-282-B	CTGACCTATCTTCCTGGACATGACGGTTCCATTGTTGCGTCTGGGT	Amplification of 3′ flanking region
	rkp-282-D	ATCGACTGGCTGGAGCAAGA	
rkp 288	rkp-288-A	TCATTGTCTCCGCCGTAGTC	Amplification of 5′ flanking region
	rkp-288-C	CTGTGGACTCGTATGCTGTTGCGCAGCGATGGCTAATTCAACGCTT	
	rkp-288-B	CTGACCTATCTTCCTGGACATGACGGCAGTACCCCGTTAGTAACCC	Amplification of 3′ flanking region
	rkp-288-D	AAACCGCGGGATATGCACTT	
rkp 302	rkp-302-A	TTGCAGAGCAAATCCGTGAA	Amplification of 5′ flanking region
	rkp-302-C	CTGTGGACTCGTATGCTGTTGCGCAGCGGCAAGAATCCTCCTAGCA	
	rkp-302-B	CTGACCTATCTTCCTGGACATGACGGGGTTATGGAAGCTTTTGCCT	Amplification of 3′ flanking region
	rkp-302-D	ATCCTGGATGGAGCCGTACT	
rkp 308	rkp-308-A	TGTCGTGCATGTCACGCTTT	Amplification of 5′ flanking region
	rkp-308-C	CTGTGGACTCGTATGCTGTTGCGCAGTTCAATAAGCCCGTTAGGCA	
	rkp-308-B	CTGACCTATCTTCCTGGACATGACGGTCCTACAAGGAGCGGAACAC	Amplification of 3′ flanking region
	rkp-308-D	TGGTAGTTCAAGCCATCGCA	
rkp 316	rkp-316-A	TCACAGGGTCATGCCACTCA	Amplification of 5′ flanking region
	rkp-316-C	CTGTGGACTCGTATGCTGTTGCGCAGGCTGATCATGCGGCACGACT	
	rkp-316-B	CTGACCTATCTTCCTGGACATGACGGTGATGGTCCCAGGTCTGCTT	Amplification of 3′ flanking region
	rkp-316-D	AGGGCTTCCTCCACGCTTTC	
rkp 317	rkp-317-A	CCATGAAGCTGTATCCGCCA	Amplification of 5′ flanking region
	rkp-317-C	CTGTGGACTCGTATGCTGTTGCGCAGGAAGGTGCCGAACCATGGTC	
	rkp-317-B	CTGACCTATCTTCCTGGACATGACGGCCTTGATGTCAGCGTCCCTT	Amplification of 3′ flanking region
	rkp-317-D	AGCAATTGCGGGGATAGCTC	
rkp 318	rkp-318-A	TGGGACTCTCTGTCATAATCGG	Amplification of 5′ flanking region
	rkp-318-C	CTGTGGACTCGTATGCTGTTGCGCAGGCTTAGACGATGCGATTGTCC	
	rkp-318-B	CTGACCTATCTTCCTGGACATGACGGTACTCCGTGGATCATCAGCA	Amplification of 3′ flanking region
	rkp-318-D	AGTCTCCCAGCATCGAACAC	
rkp 320	rkp-320-A	TCGTCTGATCGTGTTTACGG	Amplification of 5′ flanking region
	rkp-320-C	CTGTGGACTCGTATGCTGTTGCGCAGCAAATGCCCCTTGCGTGTTC	
	rkp-320-B	CTGACCTATCTTCCTGGACATGACGGGTGGCCGATCCGAATGAGAC	Amplification of 3′ flanking region
	rkp-320-D	TTGAAGTCCGTTGGGCAACT	
rkp 326	rkp-326-A	GTTACTCGGGTGTCGGACTT	Amplification of 5′ flanking region
	rkp-326-C	CTGTGGACTCGTATGCTGTTGCGCAGATTGAGCGTTCATTTTATGGCTTTG	
	rkp-326-B	CTGACCTATCTTCCTGGACATGACGGGATGTCCACCCACTTACGCA	Amplification of 3′ flanking region
	rkp-326-D	AGCGCCGACATTTTACGTCT	
rkp 327	rkp-327-A	TGGAGAAAGTTACGCTCACCA	Amplification of 5′ flanking region
	rkp-327-C	CTGTGGACTCGTATGCTGTTGCGCAGCAAGATGTCTCCCGGGAATAGG	
	rkp-327-B	CTGACCTATCTTCCTGGACATGACGGGGGGCAAAGAGTCTCGGAAA	Amplification of 3′ flanking region
	rkp-327-D	CATGCCTGATGCAGACCAGA	
rkp 328	rkp-328-A	CAGGGGAAGCTTGGCAGTAA	Amplification of 5′ flanking region
	rkp-328-C	CTGTGGACTCGTATGCTGTTGCGCAGCGCCACCGTATGCCGTTATC	
	rkp-328-B	CTGACCTATCTTCCTGGACATGACGGTGGTGGGGAAATCCTTAGTCC	Amplification of 3′ flanking region
	rkp-328-D	GGAGCTTGTGGCTCATAGGG	
rkp 333	rkp-333-A	ACTTTCGACGTGGCCGAACT	Amplification of 5′ flanking region
	rkp-333-C	CTGTGGACTCGTATGCTGTTGCGCAGTGGTTCATGGGGTCTGGAGT	
	rkp-333-B	CTGACCTATCTTCCTGGACATGACGGGTGGCAAGGCTAGCACGATG	Amplification of 3′ flanking region
	rkp-333-D	ATTCGTCTTCCCCAACCCTT	
rkp 334	rkp-334-A	TGGCGTTTCGAGTCCCATAC	Amplification of 5′ flanking region
	rkp-334-C	CTGTGGACTCGTATGCTGTTGCGCAGCTGGACAATGCTTTGAAGACGTT	
	rkp-334-B	CTGACCTATCTTCCTGGACATGACGGCTGTGAGATTGACCGCCTACT	Amplification of 3′ flanking region
	rkp-334-D	GGACTGCTGATGCGATCGAG	
rkp 344	rkp-344-A	TAAGGTGGTATGCCACGACA	Amplification of 5′ flanking region
	rkp-344-C	CTGTGGACTCGTATGCTGTTGCGCAGTGGCAAGAAGCTCGTAGGAC	
	rkp-344-B	CTGACCTATCTTCCTGGACATGACGGACACCTCACCACATCGATCA	Amplification of 3′ flanking region
	rkp-344-D	GAGCCTAAACAGGGAACCCA	
rkp 346	rkp-346-A	AGGGGTGTCGAGGTCTTCAG	Amplification of 5′ flanking region
	rkp-346-C	CTGTGGACTCGTATGCTGTTGCGCAGTATTACCCCAGGGAAGCGCA	
	rkp-346-B	CTGACCTATCTTCCTGGACATGACGGAGATTGCCCGTTTGGGGAAC	Amplification of 3′ flanking region
	rkp-346-D	CTGCCTCTCCTGTCGGATCT	
rkp 352	rkp-352-A	GCGATGAAGCTATTAGCACA	Amplification of 5′ flanking region
	rkp-352-C	CTGTGGACTCGTATGCTGTTGCGCAGGCACAAACTCCACTAAGCGA	
	rkp-352-B	CTGACCTATCTTCCTGGACATGACGGTAGCACTCAACTGTCTACCT	Amplification of 3′ flanking region
	rkp-352-D	AAAGTGGCGATCGACCAAAC	
adeA cassette	adeA-F	CCGTCATGTCCAGGAAGATAGGTCAG	Amplification of *adeA* cassette
	adeA-R	CTGCGCAACAGCATACGAGTCCACAG	
